# Theoretical Study of Hydroxylation of α- and β-Pinene by a Cytochrome P450 Monooxygenase Model

**DOI:** 10.3390/ijms24065150

**Published:** 2023-03-08

**Authors:** Janah Shaya, Lujain Aloum, Chung-Shin Lu, Peter R. Corridon, Abdulrahman Aoudi, Abeer Shunnar, Eman Alefishat, Georg Petroianu

**Affiliations:** 1 Department of Chemistry, College of Arts and Sciences, Khalifa University of Science and Technology, Abu Dhabi 127788, United Arab Emirates; 2 Department of Pharmacology, College of Medicine and Health Sciences, Khalifa University of Science and Technology, Abu Dhabi 127788, United Arab Emirates; 3 Department of General Education, National Taichung University of Science and Technology, Taichung 404, Taiwan, China; 4 Department of Immunology and Physiology, College of Medicine and Health Sciences, Khalifa University of Science and Technology, Abu Dhabi 127788, United Arab Emirates; 5 Biomedical Engineering and Healthcare Engineering Innovation Center, Khalifa University, Abu Dhabi 127788, United Arab Emirates; 6 Center for Biotechnology, Khalifa University of Science and Technology, Abu Dhabi 127788, United Arab Emirates; 7 Department of Biopharmaceutics and Clinical Pharmacy, School of Pharmacy, The University of Jordan, Amman 11972, Jordan

**Keywords:** pinene, CYP, modelling, DFT, catalysis, hydroxylation

## Abstract

Previous studies on biocatalytic transformations of pinenes by cytochrome P450 (CYP) enzymes reveal the formation of different oxygenated products from a single substrate due to the multistate reactivity of CYP and the many reactive sites in the pinene scaffold. Up until now, the detailed mechanism of these biocatalytic transformations of pinenes have not been reported. Hereby, we report a systematic theoretical study of the plausible hydrogen abstraction and hydroxylation reactions of α- and β-pinenes by CYP using the density functional theory (DFT) method. All DFT calculations in this study were based on B3LYP/LAN computational methodology using the Gaussian09 software. We used the B3LYP functional with corrections for dispersive forces, BSSE, and anharmonicity to study the mechanism and thermodynamic properties of these reactions using a bare model (without CYP) and a pinene-CYP model. According to the potential energy surface and Boltzmann distribution for radical conformers, the major reaction products of CYP-catalyzed hydrogen abstraction from β-pinene are the doublet trans (53.4%) and doublet cis (46.1%) radical conformer at delta site. The formation of doublet cis/trans hydroxylated products released a total Gibbs free energy of about 48 kcal/mol. As for alpha pinene, the most stable radicals were trans-doublet (86.4%) and cis-doublet (13.6%) at epsilon sites, and their hydroxylation products released a total of ~50 kcal/mol Gibbs free energy. Our results highlight the likely C-H abstraction and oxygen rebounding sites accounting for the multi-state of CYP (doublet, quartet, and sextet spin states) and the formation of different conformers due to the presence of cis/trans allylic hydrogen in α-pinene and β-pinene molecules.

## 1. Introduction

**Figure 1:**
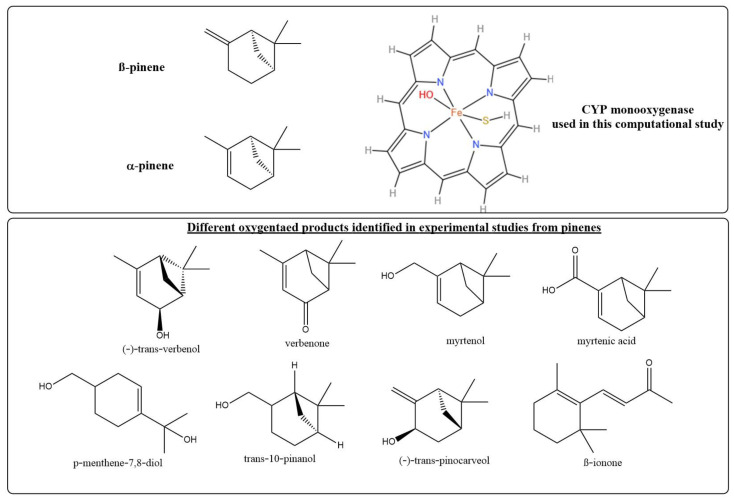
Structures of α-pinene, β-pinene, the CYP monooxygenase model, and the different oxygenated products from pinenes reported in the literature.

Cytochrome P450 (CYP) enzymes represent a notable family of biocatalysts involved in the metabolism of both exogenous and endogenous compounds using thiolate-ligated heme scaffolds (Figure 1) [1,2]. For instance, cytochrome P450 2E1 (CYP2E1) is one of the major human hepatic CYP enzymes with direct relevance in alcoholism [3]. CYPs are responsible for the metabolism of about 90% of administered drugs in men [4]. CYP enzymes were reported to catalyze a wide array of important chemical reactions such as arene oxidation, activation of inert C–H bonds, C–C coupling, epoxidation, hydroxylation, and N-dealkylation reactions [1,2,5,6,7,8,9,10]. In particular, hydroxylation reactions by CYP enzymes are among the most vital reactions in drug metabolism [3,11,12]. Most members of the CYP family are monooxygenases that activate molecular O_2_, forming a highly reactive oxidant species that can metabolize several substrates and generate various products from a single reactant [4,13,14]. The issues of the regio- and stereo-selectivity of such reactive enzymes are still hampering in spite of their versatile and important roles in the pharmaceutical industry.

Biocatalytic transformation of monoterpenes, such as those catalysed by CYPs, is an active area of research. Monoterpenes and their oxygenated products are important raw materials for a variety of value-added products such as pharmaceuticals, flavors, and fragrances [7,15,16]. Bicyclic monoterpenes such as α-pinene and β-pinene are major components of turpentine oil, the distillate of certain pine trees [17]. Important pharmacological effects [18] of α- and β-pinenes were demonstrated in several studies such as their antitumor [17,19], antimicrobial [20], and anticonvulsant [21] activities. In addition, monoterpenes represent around 11% of the volatile organic compounds emitted into the atmosphere, with α- and β-pinenes being among the most abundant terpenes in the troposphere. Monoterpenes thus play considerable roles in atmospheric chemistry where oxidation of these products can take place by atmospheric oxidants such as nitrate radicals and ozone molecules [22,23,24].

Previous studies on biocatalytic transformations of pinenes have demonstrated the formation of different oxygenated products from a single substrate due to the multistate reactivity of the enzymes and the many reactive sites in the pinene scaffold (Figure 1) [25]. For instance, in rabbits, (−)-trans-verbenol, myrtenol, and myrtenic acid were the urinary metabolites from α-pinene, while (−)-1-p-Menthene-7,8-diol and (−)-trans-10-pinanol were the major products from β-pinene [26]. Similar metabolites were found in brushtail possum fed with α- and β-pinenes [27]. Studies on bark beetle showed verbenols, verbenone, and 4-methyl-2-pentanol in α-pinene post-treatment [28,29], and trans-pinocarveol and pinocarvone in β-pinene experiments [28]. Two additional reports showed the biocatalytic conversion of α-pinene into the mountain pine beetle aggregation pheromone trans-verbenol by cytochromes P450 CYP6DE1 [30] and CYP6DE3 [31]. Likewise, CYP345E2, an antennae-specific P450 from the mountain pine beetle, was reported to epoxidize α-pinene into α-pinene oxide and hydroxylate β-pinene into (*E*)-pinocarveol [32]. More recently, cis- and trans-verbenol, myrtenol, and other metabolites such as myrtenic acid were found in α-pinene urinary metabolites in post-oral administration in humans [33]. Other examples of recent reports show the possible metabolic conversion of β-pinene to β-ionone with violet scent by CYP [34,35,36].

Several mechanistic studies on biocatalytic transformations by CYP enzymes revealed multi-state reactivity in many cases with different spin states and reaction routes that are close in energy. These results explain the generation of various products from the same initial substrate [2,37,38]. A study on CYP125 enzyme presents an interesting example of these multi-transformations caused by the competition among various possible catalytic routes. CYP125 sequentially oxidizes the terminal methyl of cholest-4-en-3-one to its alcohol, aldehyde, and lastly cholest-4en-3-one-26-acid, following the consensus catalytic cycle of most oxidizing CYPs [39]. Five additional metabolites were also identified in this reaction produced by the deformylation of the aldehyde via peroxohemiacetal formation [39]. Other studies showed that CYP-catalyzed epoxidation reaction generates one product in the doublet state and a mixture of products in the quartet state [4,40].

Thus, regarding the reactivity of CYP, both theoretical and experimental studies show no clear-cut mechanism. In this study, we focus on the hydrogen-abstraction and hydroxylation steps of pinenes catalyzed by CYP. Understanding H-abstraction reactions has a key role in deciphering complex physical organic concepts and reaction systems such as oxidation of biological systems, autoignition, and combustion of hydrocarbons, as well as oxidations in atmospheric chemistry. On the other hand, hydroxylation reactions and rebound mechanisms by CYP are vital steps in drug metabolism [4,41,42,43,44]. A detailed investigation of the possible mechanisms and interactions of the CYP/pinene system is important for predicting the various possible products observed experimentally. Up to the present, such mechanistic studies have not been reported. Herein, we present a systematic mechanistic study on the different possible hydrogen-abstraction and hydroxylation reactions of CYP on α- and β-pinenes in multi-state reactivity (different spin states) on the basis of density functional theory (DFT).

## 2. Results and Discussion

### 2.1. Alpha- and Beta-Pinene Bare Systems

We first started by computing the relative energies of the radicals and the hydroxylated products of the α-pinene and β-pinene without a CYP450 model using the B3LYP/LAN methodology. We arbitrarily named the carbon atoms in these compounds with Greek letters to identify the sites from which hydrogen atoms can be abstracted. The structures in Table 1 and Table 2 illustrate the employed nomenclature. The nu (ɳ) site was employed to differentiate the reaction pathways of the cis hydrogen (same side) and trans hydrogen (opposite site) on the epsilon C. Starting with α-pinene (Table 1), our calculations showed that the most stable radical was formed with the removal of the hydrogen atom from the epsilon site. All other radicals, with the exception of the one formed at the gamma site (~2 kcal/mol), had considerably higher energies (17–29 kcal/mol). The steric strain of the bridgehead alpha radical might have contributed to making the alpha radical considerably less stable (21.7 kcal/mol) with lack of resonance [41,42]. The order of stability of the allyl radicals was as follows: epsilon > gamma > alpha. Considering the computed energies of the hydroxylated structures, the products formed at the zeta, delta, and alpha sites were found to be more stable (0–2 kcal/mol) than the other hydroxylated ones.

With respect to β-pinene (Table 2), our geometry optimizations showed that the most stable radical was formed at the delta allylic site. Similar to the α-pinene, all the other radicals were far above this structure in the potential energy surface, with the gamma radical being the least stable (~31 kcal/mol). Also in line with the α-pinene, the tertiary bridgehead alpha radical was considerably less stable (21.9 kcal/mol) than the other allylic radical at delta carbon. The most stable hydroxylated products were also found to be the tertiary hydroxylated carbons at zeta and alpha sites.

### 2.2. CYP Monooxygenase Model

Modelling biochemical reactions is demanding, particularly with diverse enzyme families such as CYP. Several model systems have been used for CYP monooxygenases. In this study, we employed one model (Figure 1) of CYP that showed good efficiency in previous DFT calculation studies. It is important to mention that this model is representative of the CYP family but does not account for all CYP classes. The chosen Fe^4+^O^2−^(C_20_N_4_H_12_)^−^(SH)^−^ model [3,4] is composed of an unsubstituted porphyrin ring instead of a porphyrin IX ring and an SH– group instead of SMe or protein side chains as axial Cys_437_ [45].

### 2.3. R and S Enantiomers Cis/Trans Reaction

**Figure 2:**
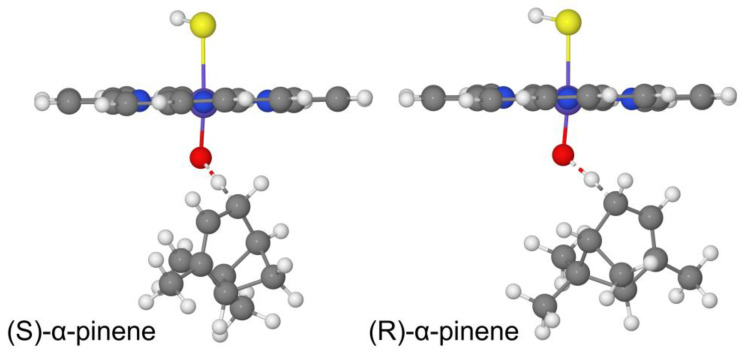
DFT/LAN level-optimized transition state structures for the hydrogen transfer to CYP through the cis reaction path in the doublet state for the R and S α-pinene enantiomers.

We first computed the cis/trans hydrogen transfer to the CYP reaction using both R and S enantiomers of α-pinene (Figure 2 and Table 3) and β-pinene (Figure S1 and Table S1, Supplementary Materials). The results show that there are no significant structural differences and consequently no significant energy differences between the two enantiomers. This result can be explained by the absence of any electronic or steric clashes differences introduced in the hydrogen transfer reaction mechanism by the R or S pinene isomers, as expected.

### 2.4. CYP-Catalyzed Hydroxylation of β-Pinene

Based on the preliminary results confirming the stability of the allyl radical from the delta site of the β-pinene and the interesting high energy of the allyl radical generated at the alpha site, we focused on studying the catalytic interactions of the CYP model with allyl-type hydrogens only. The higher stability of allyl radicals versus other non-delocalized radicals can be rationalized in terms of the delocalization of the paired electrons of the olefin bond and the non-paired electron [41,42,46].

**Figure 3:**
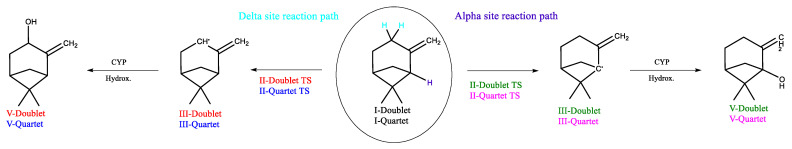
CYP-catalyzed hydroxylation of allylic sites of β-pinene.

**Figure 4:**
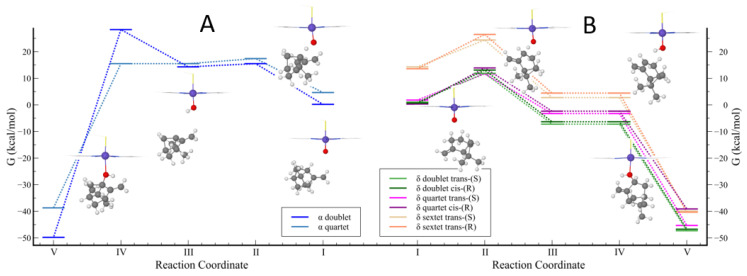
Reaction coordinates for the Gibbs free energies from the hydrogen abstraction and rebound mechanism of the β-pinene catalyzed by the CYP enzyme; (**A**) alpha and (**B**) delta. Data are provided in kcal/mol and relative to the alpha site reactant, the most stable structure in coordinate 1. The optimized structures of doublet alpha and delta trans-(S) paths are displayed in this figure.

First, we studied the catalytic C-H abstraction followed by hydroxylation of the delta site of β-pinene by CYP in the doublet, quartet, and sextet spin states since transition metal complexes (and CYP) can react through multistate reactivity patterns with several close-lying spin states [2,37,40,47,48]. Figure 3 and Figure 4 illustrate the computed mechanisms using the B3LYP/LAN methodology. Table 4 presents absolute energies, kinetic, and thermodynamic calculated parameters (see Tables S2–S10, S13 and Figure S2 in the Supplementary Materials for the coordinates of the energy profile and the calculations in detail). Since there are two hydrogen atoms in the delta carbon, the hydrogen abstraction can occur in a cis/trans fashion with respect to the η site of β-pinene. We set up to use one configuration for cis (R-cis) and the other for trans (S-trans) since our preliminary calculations of the R and S structures of the pinene system showed similar electronic energies (vide supra). The sextet spin state was found to be considerably high in energy (Figure 4B), in line with previous studies in the literature [49], so it was excluded in further calculations.

We observed that all cis transition state structures have slightly more elevated energies than the trans structures. Analysis of the structures suggests that this could be due to the steric clashes between the oxygen atom in the CYP and the β-pinene scaffold. According to our calculations, the doublet and quartet reaction paths (coordinate I in Figure 4B) have similar energies, while the paths of the sextet spin state were found to be high in energy, as mentioned. For the doublet and quartet reaction paths, the hydrogen transfer reactions are characterized by exothermicity and relatively low electronic barriers. As can be observed in Table 4, the general trend in the application of vibrational corrections to the electronic energies led to a decrease in the enthalpy of activation and in the enthalpy of reaction (except to sextet delta trans and alpha quartet structures, in which the effect is nearly null). For the entropy corrections, a less clear trend was observed. Except for the sextet high-spin states and quartet delta trans conformer, the entropic factor led to increasing the activation energy and reaction Gibbs free energy. It is largely known that the two main DFT sources of error are the missing dispersion forces and the basis set superposition error [50]. Thus, as expected, the effects of corrections added to our DFT/LAN method led to lower barriers and more favorable thermodynamics (again, except for the high-spin thermodynamic parameters) for the hydrogen transfer reaction. We observed that the addition of empirical corrections for the dispersive forces lead to a great decrease in the CYP-catalyzed reaction barriers (Figure S3). The same trend was observed by Mulholland and colleagues [51]. These lower reaction barriers are reasonable in a context of catalyzed biochemical reactions where barriers are expected to be no greater than 18 kcal/mol [52]. Moreover, we observed that the stability order of the radical products is marginally affected by these corrections, in contrast to the stability of the hydroxylated products. Therefore, the applications of these corrections are essential for more accurate predictions of the selectivity and reaction barriers.

The calculation of the allylic alpha hydrogen abstraction by CYP showed the unexpected instability of the alpha radical that was observed in the calculations using the bare β-pinene system (Figure 4A). It appears that alpha-H abstraction is destabilizing the bicyclic ring. At reaction coordinate I, the alpha site CYP + β-pinene complex in the doublet state was found to be the most stable structure (0 kcal/mol) over the potential energy surface. Nevertheless, the energy differences among other optimized structures in coordinate I were very small, excluding the sextet state. We are able to identify several other conformers in the doublet and quartet with slightly more elevated energies, as the β-pinene can be freely rotated/translated around the CYP due to its weak interaction with the enzyme. To the alpha site, our calculations showed that the activation barriers are more elevated at the doublet state than the quartet state. However, the hydrogen abstraction from this site is unlikely due to the unfavorable thermodynamics for the alpha radical formation.

We then investigated the rebound mechanism from the β-pinene radical after the hydrogen abstraction by CYP. The transition states connecting the radical and the hydroxylated product for the low and high spin states could not be found in an exhaustive potential energy surfaces search. In the quartet state, we were successful in locating these transition states structures only at a lower theoretical level (B3LYP/6-31G). Searches in the potential energy surface through scan calculations suggested a small electronics barrier of ~3.0 kcal/mol. However, at a higher theoretical level, the potential energy surface turns flat and all attempts to optimize these transition states at B3LYP/LAN level were not fruitful. Therefore, in line with previous studies, we consider that the hydroxylation rebound mechanism is barrierless at our theoretical level, with the hydrogen-abstraction by the CYP enzyme as the rate-limiting step [12].

**Figure 5:**
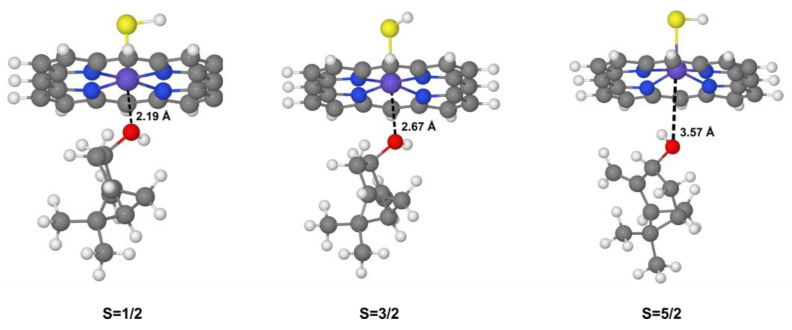
Optimized structures for delta site cis-(R) hydroxylation products of β-pinene in doublet, quartet, and sextet states. Interaction distances are provided in angstroms.

The bare system alpha-delta trend in the relative stability of hydroxylated product repeats in the CYP + β-pinene system. The doublet alpha hydroxylated product is about ~3 kcal/mol more stable than the delta hydroxylated product. We observed that high-spin sextet structures (Figure 5) do not give classical rebounded hydroxylated products. The increase of electronic repulsion around the iron atom in higher spin states repels the ligands above and below the plane occupied by the porphyrin system, evidenced by the increased interaction distance between Fe and O and the slight distortion of the Fe coordination sphere.

With these results, we can reason a dynamic equilibrium scenario for the most probable reaction path in a theoretical mixture. Table 5 shows the computed Boltzmann distribution based on the free energies of the reactant structures in the first reaction coordinate. The alpha site reactant in the doublet potential energy surface is the most stable structure, responding by about half of the conformation states in the distribution. The other half is mainly composed of delta cis/trans doublet and delta cis quartet conformers. Due to the complex electronic nature of the transition metal-containing systems, the reaction can proceed through multiple potential energy surfaces. As the structures in doublet and quartet reaction paths are very similar, nearly as degenerate states or with very close energies, we can assume that spin crossover can occur through these spin states. Indeed, experimental and theoretical evidence suggests facile interconversion between these spin states in iron porphyrin compounds [12,53].

Lastly, the calculated Boltzmann distribution for the product conformers of the H-abstraction (coordinate III) predicts approximately a 50:50 distribution around cis/trans conformers in the doublet state (Table 6). Using the Eyring equation, we can estimate the reaction through the delta trans doublet path as three and two times faster than the delta cis quartet and doublet paths, respectively. Despite the initially lower concentration in the reaction coordinate I for the trans doublet conformer, the lower activation barrier suggests greater kinetic favor to the reaction through this path. Thus, it is expected that β-pinene CYP-catalyzed hydrogen abstraction led to the formation of the doublet trans radical conformer as the major reaction product.

After the formation of the cis/trans doublet radical, the rebound mechanism can form the highly stable hydroxylated product. The formation of these structures is accompanied by the release of substantial amount of energy. The total Gibbs free energies released by the formation of doublet cis and trans hydroxylated products can be estimated as approximately −48 kcal/mol. Reversal of the hydroxylation reaction demands overcoming barriers as large as 40 kcal/mol. Thus, our results suggest that the hydroxylated product formation acts as a “thermodynamic trap” for the reaction system since the reverse reaction is kinetically hindered.

### 2.5. CYP-Catalyzed Hydroxylation of α-Pinene

**Figure 6:**
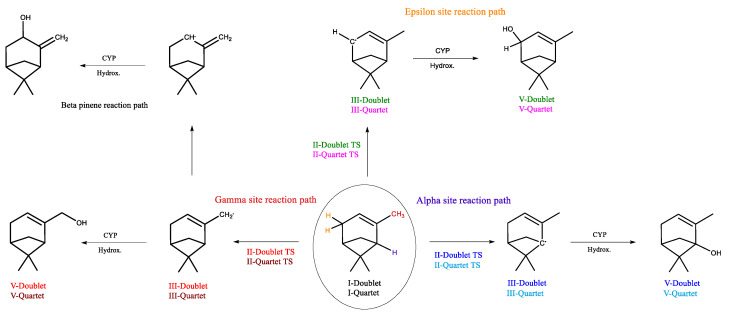
CYP-catalyzed hydroxylation of allylic sites of α-pinene.

**Figure 7:**
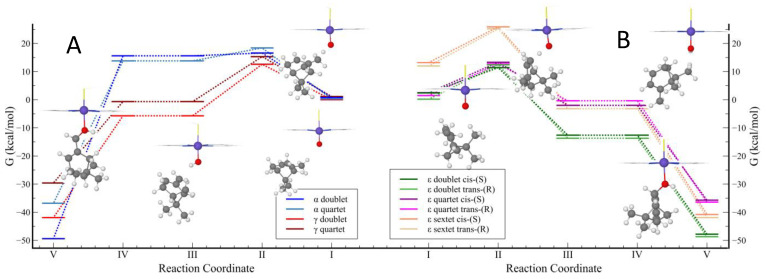
Reaction coordinates for the Gibbs free energies from the hydrogen abstraction and rebound mechanism of the α-pinene catalyzed by the CYP enzyme; (**A**) alpha and gamma, and (**B**) epsilon. Data are provided in kcal/mol and relative to the gamma site reactant, the most stable structure in coordinate 1. Optimized structures for the gamma doublet path in the left and epsilon cis-(S) doublet path in the right.

Considering the α-pinene molecule, we studied the CYP-catalyzed hydrogen abstraction followed by hydroxylation of the epsilon, alpha, and gamma allylic sites in the doublet, quartet, and sextet spin (for epsilon only) states. Figure 6 and Figure 7 (and Figure S3 and S4) show the calculated reaction coordinates. Table 7 (and Table S11, S12 and S14) presents the calculated absolute energies and the kinetic and thermodynamic parameters. As in the case of the β-pinene delta-site, the epsilon-site has two cis/trans hydrogen atoms (regarding the η site), liable for being abstracted by the CYP. The R and S configurations also had similar electronic energies. The cis transition states of hydrogen abstraction (coordinate II) had slightly higher energies than the trans transition states, except for the doublet state. The sextet state potential energy surface (PES) lies above the doublet and quartet PES. We thus excluded sextet PES from calculations at alpha and gamma sites. The reactions through the epsilon and gamma sites had favorable thermodynamic profiles and low activation barriers, whereas the reaction at the alpha site was found to be thermodynamically unfavorable. As the β-pinene, the application of vibrational corrections to the electronic energy led to a decrease in the enthalpic activation energies (Table 7) for the α-pinene reaction. The effect in the reaction enthalpy is less accentuated, but trends to an increase in the liberated energy. Except for the doublet epsilon cis conformation, the entropic effect led to an increase in the activation energy values regarding the enthalpy of activation. The results of the empirical corrections added in our DFT/LAN methodology over the pure DFT repeat the trend observed in the β-pinene, resulting in better thermodynamic and kinetic profiles for the CYP-catalyzed hydrogen transfer reaction.

Our calculations at B3LYP/LAN level showed that the most stable products were formed in the doublet state by hydrogen abstraction at the epsilon site in agreement with the calculations of the bare system. The trans product is slightly more stable; however, the reaction through the cis path has a lower activation barrier (9.00 vs. 12.2 kcal/mol) and lower Gibbs free energy (−15 vs. −13.8 kcal/mol).

As in the case of β-pinene, the transition state for the rebound mechanism was not identified. So, we consider that this mechanism is barrierless at our theoretical level with the hydrogen abstraction by the CYP enzyme as the rate-limiting step. The most stable hydroxylated rebounded product was formed at the alpha site followed by the epsilon trans product, which was only 0.7 kcal/mol less stable. The same order of the allylic products (alpha > epsilon > gamma) was obtained as in the bare system. However, the difference between alpha and epsilon is only 1 kcal/mol rather than those 5–6 kcal/mol obtained from the bare system. This indicates that the presence of CYP450 and the rebounded structure formation linked to the iron center slightly shifted the equilibrium in relation to the bare model.

With this data, we can reason the possible paths that the reaction can proceed in this theoretical scenario. The Boltzmann distribution (Table 8) related to the structures in coordinate I shows the existence of a mixture of conformers in the initial reaction mass. There is no hydrogen transfer reaction through the sextet spin state and the reaction through the alpha site is thermodynamically unfavorable. The reactions through the remaining sites have relatively low barriers and are thermodynamically feasible. However, our calculations (Table 9) showed that the most stable radicals are formed at the epsilon cis and trans path in the doublet state. The Boltzmann distribution indicates that the equilibrium is totally shifted to the formation of these products. From these structures, the hydroxylation reaction of α-pinene can proceed fast with a substantial release of ~50 kcal/mol, a little more than the ones computed for β-pinene.

To get more insight into the hydrogen transfer to the CYP model reaction, we conducted an electronic analysis of the α-pinene. We analyzed the Mulliken and NPA chargers, as well as the Mulliken and NBO spin population in doublet and quartet states for epsilon cis and trans paths (Table S12). The orbital analysis shows that the lowest orbitals shown are the bonding type orbitals along the Fe–O bond and include the σ*z*^2^ for the overlap of 3d*z*^2^ on iron with 2p*z* on oxygen and the degenerate pair of π*xz* and π*yz* molecular orbitals for the bonding interaction between the 3d*xz* (or 3d*yz*) on iron with the 2p*x* (or 2p*y*) atomic orbital on oxygen [45,54]. The antibonding combination of this pair of orbital π*xz reflects the antibonding interactions of the 3dxz orbitals on Fe with the 2py orbitals on O along the Fe–O bond [54]. The δ*x*^2^–*y*^2^ orbital is non-bonding and located in the plane of the heme/porphyrin. The two σ* antibonding orbitals are high in energy and virtual—one along the O–Fe–S axis (the *z*-axis) [2,55], namely σ*z2, is seen in Figure S4.

## 3. Materials and Methods 

All DFT calculations were conducted in the Gaussian09 software (dftd4 version 3.2.0 from GitHub) [56] with the well-known three-parameter hybrid functional of Becke (B3) [57,58] in combination with the Lee-Yang-Parr (LYP) [59,60] correlation functional. The B3LYP hybrid functional with unrestricted formalism has been largely used in the theoretical studies for modeling active sites and analyzing reaction mechanisms in many systems, particularly CYP [3,61] and Heme [62] models of enzymes, where it was shown to give correct spin-state energetics for these species [2,4,38]. Studies have shown that this functional can provide accurate geometries and reasonable agreement with Coupled Cluster Singles and Doubles Theory (CCSD(T)) calculations of the relative spin states energies for porphyrin [63] complexes and heme-related [62,64] models. For geometry optimizations, vibrational analyses, and Intrinsic Reaction Coordinate (IRC) calculations, we employed the Pople double-zeta polarized 6-31G(d) basis set [65,66] for all atoms, except iron. In this atom, the orbitals are expanded with the LANL2DZ [67,68,69] ECP basis set. The default Gaussian algorithm (Berny algorithm using GEDIIS) was adopted to minimize energy in the minimum and maximum stationary points. In the optimization process, we consider that structural convergence occurs only when the maximum force and its root mean square (RMS) fall below 4.5 × 10^−4^ and 3 × 10^−4^ hartree/bohr. We fix a threshold of 1.8 × 10^−3^ and 4 × 10^−3^ bohr for the maximum displacement, and its RMS with the default grid for the numerical integration step in all calculations. Low-lying harmonic vibrational mode’s contribution to entropy frequencies obtained from calculations were treated using a free-rotor approximation [70] (quasi-rigid-rotor-harmonic-oscillator) implemented in the GoodVibes software (GoodVibes version 3.1.1 from GitHub) [49]. To address the basis set superposition error (BSSE), we added Grimme geometrical counterpoise gCP [71,72] correction for all computed energies. Dispersion effects may play a relevant role in CYP-like systems [73,74]. Therefore, to assure the correct dispersion computation, we added the D4 Grimme’s empirical corrections [75,76] for all computed energies via external software (Gcp version 2.3.1 from GitHub). The Polarizable Continuum Model (PCM) [77,78] was used to simulate the water effect in this molecular system. This computational methodology will be referred to as B3LYP/LAN in the manuscript.

## 4. Conclusions

To conclude, we report a systematic mechanistic study of biocatalytic hydrogen abstraction and hydroxylation of α- and β-pinenes by multi-state cytochrome P450 enzymes (CYP) based on the density functional theory (DFT) method using the Gaussian09 software and B3LYP/LAN computational methodology. CYPs are an interesting class of biocatalysts as they can generate several products from the same substrate due to the presence of multi-states (different spin states). In our DFT study, we started by studying the hydrogen abstraction and hydroxylation of α-pinene and β-pinene without a CYP450 model using the B3LYP/LAN methodology (bare system) as a reference to the subsequent CYP-catalyzed hydroxylation of β-pinene and CYP-catalyzed hydroxylation of α-pinene accounting for the doublet, quartet, and sextet spin states of CYP. According to the potential energy surface and Boltzmann distribution for radical conformers, the major reaction products of CYP-catalyzed hydrogen abstraction from β-pinene were the doublet trans (53.4%) and doublet cis (46.1%) radical conformers at the delta site, and the formation of doublet cis/trans hydroxylated products released a total Gibbs free energy of ~48 kcal/mol. As for α-pinene, the most stable radicals were trans-doublet (86.4%) and cis-doublet (13.6%) at epsilon sites, and their hydroxylation products released a total of ~50 kcal/mol Gibbs free energy. The transition state connecting the radical (from hydrogen abstraction) and the hydroxylated product (from oxygen rebound) was not located. The rebound mechanism is considered barrierless at our mentioned theoretical level, in line with previous studies in the literature, and the hydrogen abstraction by CYP is the rate-limiting step. 

## Figures and Tables

**Table 1:** Computed relative energies of the radicals and hydroxylated products of α-pinene. The term “cor” refers to the dispersion, BSSE, and Grimme quasi-harmonic corrections to the calculated absolute Gibbs free energies (in Hartree). Energies in kcal/mol were calculated with reference to the most stable conformation for the hydroxylated radical and product.

	Radical	G_cor_(Eh)	G_cor(ref)_ (kcal/mol)	Hydroxylated Product	G_cor_(Eh)	G_cor(ref)_ (kcal/mol)
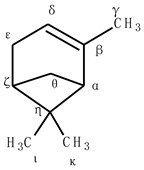	Alpha	−389.788789	21.7	Alpha	−465.658506	1.4
	Gamma	−389.819706	2.27	Gamma	−465.645667	9.45
	Delta	−389.777607	28.7	Delta	−465.660286	0.28
	Epsilon	−389.823321	0	Epsilon cis	−465.651306	5.91
				Epsilon trans	−465.652273	5.31
	Zeta	−389.790994	20.3	Zeta	−465.660730	0.00
	Theta	−389.794206	18.3	Theta	−465.654240	4.07
	Iota	−389.795916	17.2	Iota	−465.646394	9
	Kappa	−389.796285	17	Kappa	−465.646120	9.17

**Table 2:** Computed relative energies of the radicals and hydroxylated products of β-pinene. The term “cor” refers to the dispersion, BSSE, and Grimme quasi-harmonic corrections to the calculated absolute Gibbs free energies (in Hartree). Energies in kcal/mol were calculated with reference to the most stable conformation for the hydroxylated radical and product.

	Radical	G_cor_ (Eh)	G_cor(ref)_ (kcal/mol)	Hydroxylated Product	G_cor_ (Eh)	G_cor(ref)_ (kcal/mol)
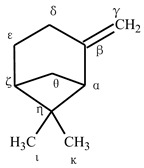	Alpha	−389.784833	21.9	Alpha	−465.653831	0.57
	Gamma	−389.770224	31.1	Gamma	−465.649621	3.21
	Delta	−389.819707	0	Delta cis	−465.645132	6.03
				Delta trans	−465.644727	6.28
	Epsilon	−389.797584	13.9	Epsilon cis	−465.6451796	6.00
				Epsilon trans	−465.6473512	4.63
	Zeta	−389.786313	20.9	Zeta	−465.6547380	0
	Theta	−389.788711	19.4	Theta	−465.6488230	3.71
	Iota	−389.789920	18.7	Iota	−465.6375598	10.78
	Kappa	−389.790510	18.3	Kappa	−465.6399752	9.26

**Table 3:** Gibbs free energy coordinates of the reaction R/S and cis/trans paths for the α-pinene. Data are provided in kcal/mol and relative to the most stable structure formed in reaction coordinate 1, the gamma conformer. RC is reaction coordinate.

RC	Site	Spin	G_ref_ (kcal/mol)	RC	Site	Spin	G_ref_ (kcal/mol)
1	Epsilon cis-(S)	S = 1/2	2.41	1	Epsilon cis-(R)	S = 1/2	2.40
2			11.4	2			11.4
3			−12.6	3			−12.6
1	Epsilon trans-(S)	S = 1/2	0.184	1	Epsilon trans-(R)	S = 1/2	0.184
2			12.4	2			12.4
3			−13.7	3			−13.7

**Table 4:** Thermodynamic and kinetic data computed for the hydrogen capture reaction by CYP in β-pinene. The terms Δ_r_E, Δ_r_H, and Δ_r_G refer to electronic, enthalpic, and Gibbs free energy variation in the reaction, respectively. The terms ΔE^‡^, ΔH^‡^, and ΔG^‡^ refer to the electronic, enthalpic, and Gibbs free energy reaction barriers, respectively, and the term “cor” refers to the dispersion, BSSE, and Grimme quasi-harmonic corrections to the calculated absolute Gibbs free energies. ^Hyd^Δ_r_G_cor_ refers to the hydroxylation free energies computed as the difference between the hydroxylated product and the reactant in coordinate 1. Data are displayed in kcal/mol.

Site	Spin	Δ_r_E	ΔE^‡^	Δ_r_H	ΔH^‡^	Δ_r_G	ΔG^‡^	Δ_r_G_cor_	ΔG^‡^_cor_	^Hyd^Δ_r_G_cor_
Delta cis-(R)	S = 1/2	−4.11	17.4	−6.13	12.8	−3.82	17.6	−7.05	12.4	−47.4
Delta trans-(S)	S = 1/2	−4.64	15.4	−5.56	10.9	−5.10	15.4	−8.30	10.8	−48.4
Delta cis-(R)	S = 3/2	−0.183	18.7	−1.59	13.8	−0.548	18.8	−2.64	13.6	−45.6
Delta trans-(S)	S = 3/2	−1.03	16.5	−1.16	13.0	−3.67	13.4	−5.08	9.87	−47.3
Delta cis-(R)	S = 5/2	−6.52	19.6	−7.19	15.9	−13.3	15.8	−9.18	12.8	−54.0
Delta trans-(S)	S = 5/2	−7.36	17.3	−7.35	14.4	−15.1	12.5	−11.5	10.1	−54.2
Alpha	S = 1/2	+17.4	21.5	+15.4	16.8	+18.9	22.2	+17.4	15.3	−50.0
Alpha	S = 3/2	+13.0	18.8	+13.6	15.3	+14.3	18.5	+10.8	12.8	−43.4

**Table 5:** Boltzmann distribution for conformers present in reaction coordinate I for the β-pinene.

Reactant	G (hartree)	Mole Fraction	Population %	Δ_r_G	ΔG^‡^
alpha-doublet	−1976.037587	0.49	48.77	28.1	15.3
alpha-quartet	−1976.030510	0	0.03	10.8	12.8
delta-trans-doublet	−1976.035861	0.08	7.83	−8.3	10.8
delta-cis-doublet	−1976.036419	0.14	14.14	−7.05	12.4
delta-cis-quartet	−1976.037104	0.29	29.23	−2.64	13.6
delta-trans-quartet	−1976.014863	0	0	−5.08	9.87
delta-cis-sextet	−1976.015837	0	0	−9.18	12.8
delta-trans-sextet	−1976.014863	0	0	−11.5	10.1

**Table 6:** Boltzmann distribution for radical conformers present in reaction coordinate III for the β-pinene.

Reactant	G (hartree)	Mole Fraction	Population %	Δ_r_G	ΔG^‡^
alpha-doublet	−1975.973971	0	0	28.1	15.3
alpha-quartet	−1975.973075	0	0	10.8	12.8
delta-trans-doublet	−1976.009716	0.534	53.4	−8.3	10.8
delta-cis-doublet	−1976.009578	0.461	46.1	−7.05	12.4
delta-cis-quartet	−1976.004151	0.00147	0.147	−2.64	13.6
delta-trans-quartet	−1976.004891	0.00321	0.321	−5.08	9.87
delta-cis-sextet	−1976.000132	0	0	−9.18	12.8
delta-trans-sextet	−1976.001255	0	0	−11.5	10.1

**Table 7:** Thermodynamic and kinetic data computed for the hydrogen capture reaction by CYP in α-pinene. The terms Δ_r_E, Δ_r_H, and Δ_r_G refer to electronic, enthalpic, and Gibbs free energy variation in the reaction, respectively. The terms ΔE^‡^, ΔH^‡^, and ΔG^‡^ refer to the electronic, enthalpic, and Gibbs free energy reaction barriers, respectively, and the term “cor” refers to the dispersion, BSSE, and Grimme quasi-harmonic corrections to the calculated absolute Gibbs free energies. ^Hyd^Δ_r_G_cor_ refers to the hydroxylation free energies computed as the difference between the hydroxylated product and the reactant in coordinate 1. Data are displayed in kcal/mol.

Site	Spin	Δ_r_E	ΔE^‡^	Δ_r_H	ΔH^‡^	Δ_r_G	ΔG^‡^	Δ_r_G_cor_	ΔG^‡^_cor_	^Hyd^Δ_r_G_cor_
Epsilon cis-(S)	S = 1/2	−13.6	15.9	−12.9	12.9	−13.4	12.5	−15.0	9.00	−50.2
Epsilon trans-(R)	S = 1/2	−14.8	14.6	−14.8	11.0	−10.5	16.6	−13.8	12.2	−48.9
Epsilon cis-(S)	S = 3/2	−0.1	18.0	−0.215	14.1	−0.981	16.4	−3.06	12.3	−36.8
Epsilon trans-(R)	S = 3/2	+0.814	16.7	+0.413	12.7	−1.10	15.0	−1.87	11.2	−37.9
Epsilon cis-(S)	S = 5/2	−16.0	18.7	−15.8	14.7	−18.6	15.5	−18.7	12.7	−53.9
Epsilon trans-(R)	S = 5/2	−15.7	17.5	−16.8	13.0	−14.1	17.3	−15.2	13.2	−53.9
Alpha	S = 1/2	+18.4	22.8	+18.0	18.7	+18.3	21.4	+14.8	15.9	−50.2
Alpha	S = 3/2	+16.7	24.9	+15.9	20.6	+15.7	23.1	+13.5	18.1	−37.1
Gamma	S = 1/2	−3.18	16.1	−4.05	12.5	−0.516	16.9	−5.73	12.6	−41.9
Gamma	S = 3/2	+1.09	18.0	+0.612	14.0	+0.437	15.8	−1.80	14.2	−30.8

**Table 8:** Boltzmann distribution for conformers present in reaction coordinate I for the α-pinene.

Reactant	G (hartree)	Mole Fraction	Population %	Δ_r_G	ΔG^‡^
alpha-doublet	−1976.041937	0.0871	8.71	14.8	17.6
alpha-quartet	−1976.024096	0.209	20.9	13.5	18.1
epsilon-trans-doublet	−1976.042897	0.241	24.1	−13.8	12.2
epsilon-cis-doublet	−1976.039343	0.00557	0.557	−15.0	8.99
epsilon-trans-quartet	−1976.040800	0.0261	2.61	−1.87	11.2
epsilon-cis-quartet	−1976.041490	0.0542	5.42	−3.06	12.3
epsilon-trans-sextet	−1976.024055	0	0	−15.2	13.2
epsilon-cis-sextet	−1976.022164	0	0	−18.7	12.7
gamma-doublet	−1976.043191	0.329	32.9	−5.73	12.6
gamma-quartet	−1976.041393	0.0489	4.89	−1.80	14.2

**Table 9:** Boltzmann distribution for radical conformers present in reaction coordinate III for the α-pinene.

Reactant	G (hartree)	Mole Fraction	Population %	Δ_r_G	ΔG^‡^
alpha-doublet	−1976.018408	0	0	14.8	17.6
alpha-quartet	−1976.021248	0	0	13.5	18.1
epsilon-trans-doublet	−1976.064967	0.864	86.4	−13.8	12.2
epsilon-cis-doublet	−1976.063223	0.136	13.6	−15.0	8.99
epsilon-trans-quartet	−1976.043787	0	0	−1.87	11.2
epsilon-cis-quartet	−1976.046371	0	0	−3.06	12.3
epsilon-trans-sextet	−1976.048297	0	0	−15.2	13.2
epsilon-cis-sextet	−1976.051903	0	0	−18.7	12.7
gamma-doublet	−1976.052329	0	0	−5.73	12.6
gamma-quartet	−1976.044266	0	0	−1.80	14.2

## Data Availability

The raw and processed data required to reproduce the above findings are available in the manuscript and the Supplementary Materials.

## Supplementary Materials

The following supporting information can be downloaded at: https://www.mdpi.com/article/10.3390/ijms24065150/s1.
