# Supplementary material for: Theoretical Study of Hydroxylation of α- and β-Pinene by a Cytochrome P450 Monooxygenase Model
Source: Int J Mol Sci. 2023 Mar 8;24(6):5150. doi: 10.3390/ijms24065150 (PMC10048887; doi:10.3390/ijms24065150)
Supplement: Supplementary file 1 [file ijms-24-05150-s001.zip › ijms-2195006-supplementary.pdf]

Biocatalytic hydroxylation of α- and β-Pinene by Cytochrome P450: A systematic DFT study

Janah Shaya,* Lujain Aloum, Chungshin Lu, Peter Corridon, Abdulrahman Aoudi, Abeer Shunnar, Eman Alefishet, Georg Petroianu*

Table of contents

Figure S1: DFT/LAN level optimized transition state structures for the hydrogen transfer to CYP through trans reaction path in the doublet state for the R and S β-pinene enantiomers.

Table S1: Gibbs free energy coordinates of the reaction R/S and cis/trans paths for the β -pinene. Data are provided in kcal/mol.

Table S2: Coordinates of the reaction pathways displayed in Figure 4 (β-pinene). Data are provided in kcal/mol.

Table S3-10. Absolute energies, kinetic, and thermodynamic calculated parameters of the hydrogen abstraction and rebound mechanism of the β-pinene catalyzed by CYP enzyme

Figure S2: Gibbs free energy for the β-pinene reaction coordinated. DFT/LAN refers to our standard method whereas B3LYP to the results obtained with the B3LYP functional and mixed basis set approach (LANL2DZ on iron atom and 6-31G(d) in all remaining atoms) without D4 and BSSE.

Table S11: Coordinates to the reaction paths displayed in Figure 7 (α-pinene). Data are provided in kcal/mol.

Figure S3: Reaction coordinate for the Gibbs free energies from the hydrogen abstraction and rebound mechanism of the α-pinene catalyzed by CYP enzyme.

Table S12 and Figure S4: electronic analysis of α-pinene: Mulliken and NPA chargers and spin population in doublet and quartet states for epsilon cis and trans paths

Table S13: Coordinates Beta-Pinene

Table S14: Coordinates Alpha-Pinene

β -pinene

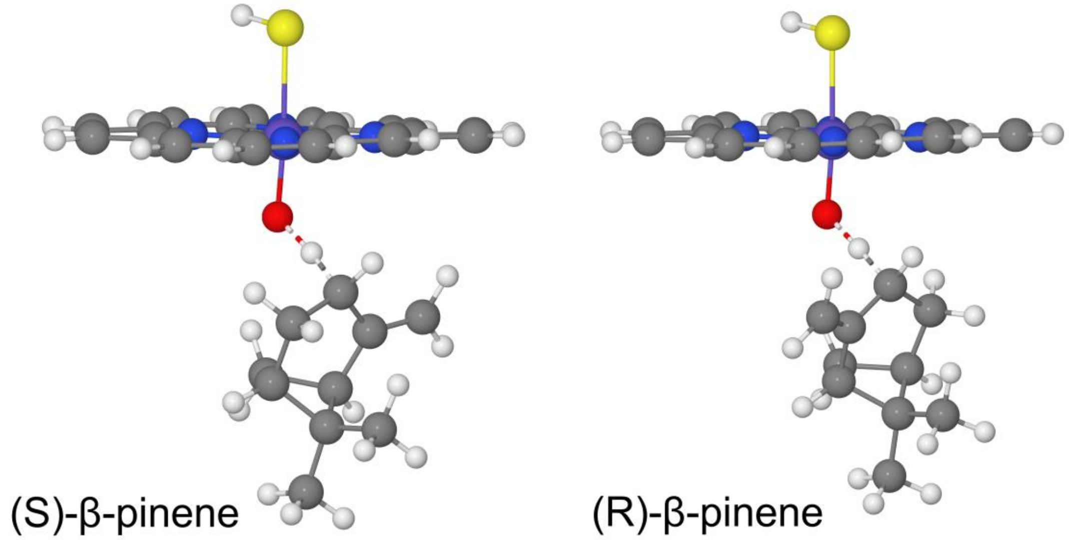

Figure S1: DFT/LAN level optimized transition state structures for the hydrogen transfer to CYP through trans reaction path in the doublet state for the R and S β-pinene enantiomers.

Table S1: Gibbs free energy coordinates of the reaction R/S and cis/trans paths for the β - pinene. Data are provided in kcal/mol.

RC	Site	Spin	G_{ref} (kcal/mol)		RC	Site	Spin	G_{ref} (kcal/mol)
1	Delta cis-(S)	S=1/2	0.733		1	Delta cis-(R)	S=1/2	0.733
2			13.1		2			13.1
3			-6.57		3			-6.32
1	Delta trans-(S)	S=1/2	1.08		1	delta trans-(R)	S=1/2	1.07
2			11.9		2			11.9
3			-7.22		3			-7.26

Table S2: Coordinates of the reaction pathways displayed in Figure 4 (β-pinene). Data are provided in kcal/mol.

RC	Site	Spin	G_{ref} (kcal/mol)		RC	Site	Spin	G_{ref} (kcal/mol)
1	Delta cis-(R)	S=1/2	0.733		1	Delta trans-(S)	S=1/2	1.08
2			13.1		2			11.9
3			-6.32		3			-7.22
5			-46.7		5			-47.3
1	Delta cis-(R)	S=3/2	0.303		1	Delta trans-(S)	S=3/2	1.83
2			13.9		2			11.7
3			-2.34		3			-3.25
5			-43.0		5			-45.3
1	Delta cis-(R)	S=5/2	13.6		1	Delta trans-(S)	S=5/2	14.3
2			26.5		2			24.4
3			4.46		3			2.76
5			-40.3		5			-39.9
1	Alpha	S=1/2	0		1	Alpha	S=3/2	4.44
2			15.3		2			17.2
3			14.0		3			15.3
5			-50.0		5			-38.9

Table S3-10. Absolute energies, kinetic, and thermodynamic calculated parameters of the hydrogen abstraction and rebound mechanism of the β-pinene catalyzed by CYP enzyme

Table S3

<table>
<tr><th>RC</th><th>Site</th><th>Spin</th><th colspan="2">E (hartree)</th><th colspan="2">H (hartree)</th><th colspan="2">G (hartree)</th><th colspan="2">G_{corr} (hartree)</th><th>G_{ref} (kcal/mol)</th></tr>
<tr><td>1</td><td rowspan="7">Delta cis-(R)</td><td rowspan="7">S=1/2</td><td colspan="2">-1976.464644</td><td colspan="2">-1975.905984</td><td colspan="2">-1976.010839</td><td colspan="2">-1976.036419</td><td>0.733</td></tr>
<tr><td>2</td><td colspan="2">-1976.436944</td><td colspan="2">-1975.885510</td><td colspan="2">r-1975.982825</td><td colspan="2">-1976.016663</td><td>13.1</td></tr>
<tr><td>3</td><td colspan="2">-1976.471199</td><td colspan="2">-1975.915749</td><td colspan="2">-1976.016923</td><td colspan="2">-1976.047659</td><td>-6.32</td></tr>
<tr><td>4</td><td colspan="2">-1976.542622</td><td colspan="2">-1975.981161</td><td colspan="2">-1976.077844</td><td colspan="2">-1976.047659</td><td>-46.7</td></tr>
<tr><td></td><td>$\Delta_r E$</td><td>$\Delta E^{\ddagger}$</td><td>$\Delta_r H$</td><td>$\Delta H^{\ddagger}$</td><td>$\Delta_r G$</td><td>$\Delta G^{\ddagger}$</td><td>$\Delta_r G$</td><td>$\Delta G^{\ddagger}$</td><td></td></tr>
<tr><td></td><td>-6.13</td><td>14.3</td><td>-8.14</td><td>9.80</td><td>-5.83</td><td>14.5</td><td>-7.05</td><td>12.4</td><td></td></tr>
<tr><td>5</td><td colspan="2">-1976.542622</td><td colspan="2">-1975.981161</td><td colspan="2">-1976.077844</td><td colspan="2">-1976.047659</td><td>-46.7</td></tr>
</table>

Table S4

<table>
<tr><th>RC</th><th>Site</th><th>Spin</th><th colspan="2">E (hartree)</th><th colspan="2">H (hartree)</th><th colspan="2">G (hartree)</th><th colspan="2">G_{corr} (hartree)</th><th>G_{ref} (kcal/mol)</th></tr>
<tr><td>1</td><td rowspan="6">Delta trans-(S)</td><td rowspan="6">S=1/2</td><td colspan="2">-1976.464943</td><td colspan="2">-1975.907286</td><td colspan="2">-1976.008524</td><td colspan="2">-1976.035861</td><td>1.08</td></tr>
<tr><td>2</td><td colspan="2">-1976.440476</td><td colspan="2">-1975.88986</td><td colspan="2">-1975.983947</td><td colspan="2">-1976.018613</td><td>11.9</td></tr>
<tr><td>3</td><td colspan="2">-1976.472341</td><td colspan="2">-1975.916147</td><td colspan="2">-1976.01666</td><td colspan="2">-1976.049090</td><td>-7.22</td></tr>
<tr><td></td><td>$\Delta_r E$</td><td>$\Delta E^{\ddagger}$</td><td>$\Delta_r H$</td><td>$\Delta H^{\ddagger}$</td><td>$\Delta_r G$</td><td>$\Delta G^{\ddagger}$</td><td>$\Delta_r G$</td><td>$\Delta G^{\ddagger}$</td><td></td></tr>
<tr><td></td><td>-7.15</td><td>12.7</td><td>-8.07</td><td>8.28</td><td>-7.61</td><td>12.8</td><td>-8.30</td><td>10.8</td><td></td></tr>
<tr><td>5</td><td colspan="2">-1976.543869</td><td colspan="2">-1975.982237</td><td colspan="2">-1976.077783</td><td colspan="2">-1976.113000</td><td>-47.3</td></tr>
</table>

Table S5

<table>
<tr><th>RC</th><th>Site</th><th>Spin</th><th colspan="2">E (hartree)</th><th colspan="2">H (hartree)</th><th colspan="2">G (hartree)</th><th colspan="2">G_{corr} (hartree)</th><th>G_{ref} (kcal/mol)</th></tr>
<tr><td>1</td><td rowspan="6">Delta cis-(R)</td><td rowspan="6">S=3/2</td><td colspan="2">-1976.46457</td><td colspan="2">-1975.905903</td><td colspan="2">-1976.011667</td><td colspan="2">-1976.037104</td><td>0.303</td></tr>
<tr><td>2</td><td colspan="2">-1976.434772</td><td colspan="2">-1975.883835</td><td colspan="2">-1975.981656</td><td colspan="2">-1976.015384</td><td>13.9</td></tr>
<tr><td>3</td><td colspan="2">-1976.464861</td><td colspan="2">-1975.908434</td><td colspan="2">-1976.012541</td><td colspan="2">-1976.411318</td><td>-2.34</td></tr>
<tr><td></td><td>$\Delta_r E$</td><td>$\Delta E^{\ddagger}$</td><td>$\Delta_r H$</td><td>$\Delta H^{\ddagger}$</td><td>$\Delta_r G$</td><td>$\Delta G^{\ddagger}$</td><td>$\Delta_r G$</td><td>$\Delta G^{\ddagger}$</td><td></td></tr>
<tr><td></td><td>-1.56</td><td>15.8</td><td>-2.97</td><td>11.0</td><td>-1.93</td><td>16.0</td><td>-2.64</td><td>13.6</td><td></td></tr>
<tr><td>5</td><td colspan="2">-1976.541342</td><td colspan="2">-1975.9804</td><td colspan="2">-1976.083168</td><td colspan="2">-1976.106051</td><td>-43.0</td></tr>
</table>

Table S6

<table>
<tr><th>RC</th><th>Site</th><th>Spin</th><th colspan="2">E (hartree)</th><th colspan="2">H (hartree)</th><th colspan="2">G (hartree)</th><th colspan="2">G_{corr} (hartree)</th><th>G_{ref} (kcal/mol)</th></tr>
<tr><td>1</td><td rowspan="4">Delta trans-(S)</td><td rowspan="4">S=3/2</td><td colspan="2">-1976.464849</td><td colspan="2">-1975.908104</td><td colspan="2">-1976.006718</td><td colspan="2">-1976.014863</td><td>1.83</td></tr>
<tr><td>2</td><td colspan="2">-1976.438523</td><td colspan="2">-1975.887415</td><td colspan="2">-1975.985353</td><td colspan="2">-1976.998706</td><td>11.7</td></tr>
<tr><td>3</td><td colspan="2">-1976.466488</td><td colspan="2">-1975.909948</td><td colspan="2">-1976.01256</td><td colspan="2">-1976.033183</td><td>-3.25</td></tr>
<tr><td></td><td>$\Delta_r E$</td><td>$\Delta E^{\ddagger}$</td><td>$\Delta_r H$</td><td>$\Delta H^{\ddagger}$</td><td>$\Delta_r G$</td><td>$\Delta G^{\ddagger}$</td><td>$\Delta_r G$</td><td>$\Delta G^{\ddagger}$</td><td></td></tr>
</table>

			-2.80	13.7	-2.93	10.1	-5.44	10.6	-5.08	9.87	
5			-1976.541093		-1975.980155		-1976.081889		-1976.109765		-45.3

Table S7

**RC**	**Site**	**Spin**	**E (hartree)**		**H (hartree)**		**G (hartree)**		**G_{corr} (hartree)**		**G_{ref} (kcal/mol)**
1	Delta cis-(R)	S=5/2	-1976.443747		-1975.888701		-1975.988506		-1976.015838		13.6
2			-1976.412542		-1975.863375		-1975.963336		-1975.995419		26.5
3			-1976.454139		-1975.900156		-1976.009698		-1976.030473		4.46
			**Δ_rE**	**ΔE‡**	**Δ_rH**	**ΔH‡**	**Δ_rG**	**ΔG‡**	**Δ_rG**	**ΔG‡**	
			-4.05	17.1	-4.71	13.4	-10.8	13.3	-9.18	12.8	
5			-1976.536271		-1975.976652		-1976.079552		-1976.071875		-40.3

Table S8

**RC**	**Site**	**Spin**	**E (hartree)**		**H (hartree)**		**G (hartree)**		**G_{corr} (hartree)**		**G_{ref} (kcal/mol)**
1	Delta trans-(S)	S=5/2	-1976.444039		-1975.889953		-1975.986953		-1976.014863		14.3
2			-1976.41644		-1975.866951		-1975.967057		-1975.998707		24.4
3			-1976.455762		-1975.901662		-1976.01097		-1976.033183		2.76
			**Δ_rE**	**ΔE‡**	**Δ_rH**	**ΔH‡**	**Δ_rG**	**ΔG‡**	**Δ_rG**	**ΔG‡**	
			-6.04	14.8	-6.03	11.9	-13.8	10.0	-11.5	10.1	
5			-1976.536637		-1975.976788		-1976.080699		-1976.101882		-39.9

Table S9

**RC**	**Site**	**Spin**	**E (hartree)**		**H (hartree)**		**G (hartree)**		**G_{corr} (hartree)**		**G_{ref} (kcal/mol)**
1	Alpha	S=1/2	-1976.46467		-1975.906005		-1976.010884		-1976.037587		0.00
2			-1976.430406		-1975.879146		-1975.975541		-1976.013197		15.3
3											
			**Δ_rE**	**ΔE‡**	**Δ_rH**	**ΔH‡**	**Δ_rG**	**ΔG‡**	**Δ_rG**	**ΔG‡**	
			28.2	17.2	27.0	12.5	29.3	17.9	28.1	15.3	
5			-1976.544394		-1975.983319		-1976.079385		-1976.117318		-50.0

Table S10

**RC**	**Site**	**Spin**	**E (hartree)**	**H (hartree)**	**G (hartree)**	**G_{corr} (hartree)**	**G_{ref} (kcal/mol)**
1	Alpha	S=3/2	-1976.456701	-1975.900247	-1976.003103	-1976.030510	4.44
2			-1976.426693	-1975.87586	-1975.973519	-1976.010128	17.2
3			-1976.43598	-1975.878618	-1975.980278	-1976.013237	15.3

			**Δ_rE**	**ΔE‡**	**Δ_rH**	**ΔH‡**	**Δ_rG**	**ΔG‡**	**Δ_rG**	**ΔG‡**	
			9.89	14.4	10.5	10.9	11.2	14.2	10.8	12.8	
5			-1976.527012		-1975.967299		-1976.070319		-1976.099648		-38.9

Figure S2: Gibbs free energy for the β-pinene reaction coordinated. DFT/LAN refers to our standard method whereas B3LYP to the results obtained with the B3LYP functional and mixed basis set approach (LANL2DZ on iron atom and 6-31G(d) in all remaining atoms) without D4 and BSSE.

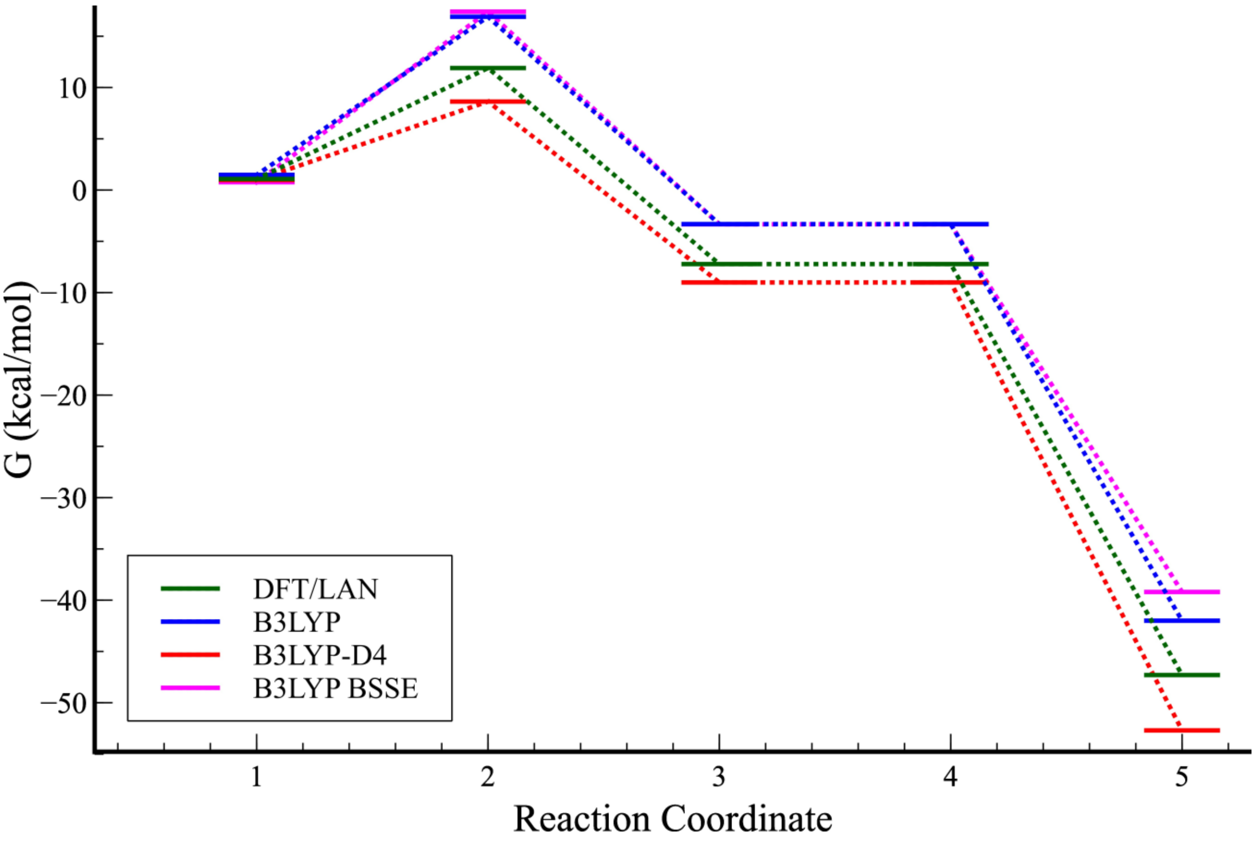

α-pinene

Table S11: Coordinates to the reaction paths displayed in Figure 7 (α-pinene). Data are provided in kcal/mol.

RC	Site	Spin	G_{ref} (kcal/mol)		RC	Site	Spin	G_{ref} (kcal/mol)
1	Epsilon cis-(S)	S=1/2	2.41		1	Epsilon trans-(R)	S=1/2	0.184
2			11.4		2			12.4
3			-12.6		3			-13.7
5			-47.8		5			-48.7
1	Epsilon cis-(S)	S=3/2	1.07		1	Epsilon trans-(R)	S=3/2	1.50
2			13.3		2			12.7
3			-2.00		3			-0.374
5			-35.7		5			-36.4
1	Epsilon cis-(S)	S=5/2	13.2		1	Epsilon trans-(R)	S=5/2	12.0
2			25.9		2			25.2
3			-5.47		3			-3.20
5			-40.8		5			-41.9
1	Alpha	S=1/2	0.787		1	Alpha	S=3/2	2.0
2			16.6		2			18.4
3			15.6		3			13.8
5			-49.4		5			-36.8
1	Gamma	S=1/2	0		1	Gamma	S=1/2	1.13
2			12.6		2			15.3
3			-5.73		3			-0.675
5			-41.9		5			-29.6

Figure S3: Reaction coordinate for the Gibbs free energies from the hydrogen abstraction and rebound mechanism of the α-pinene catalyzed by CYP enzyme.

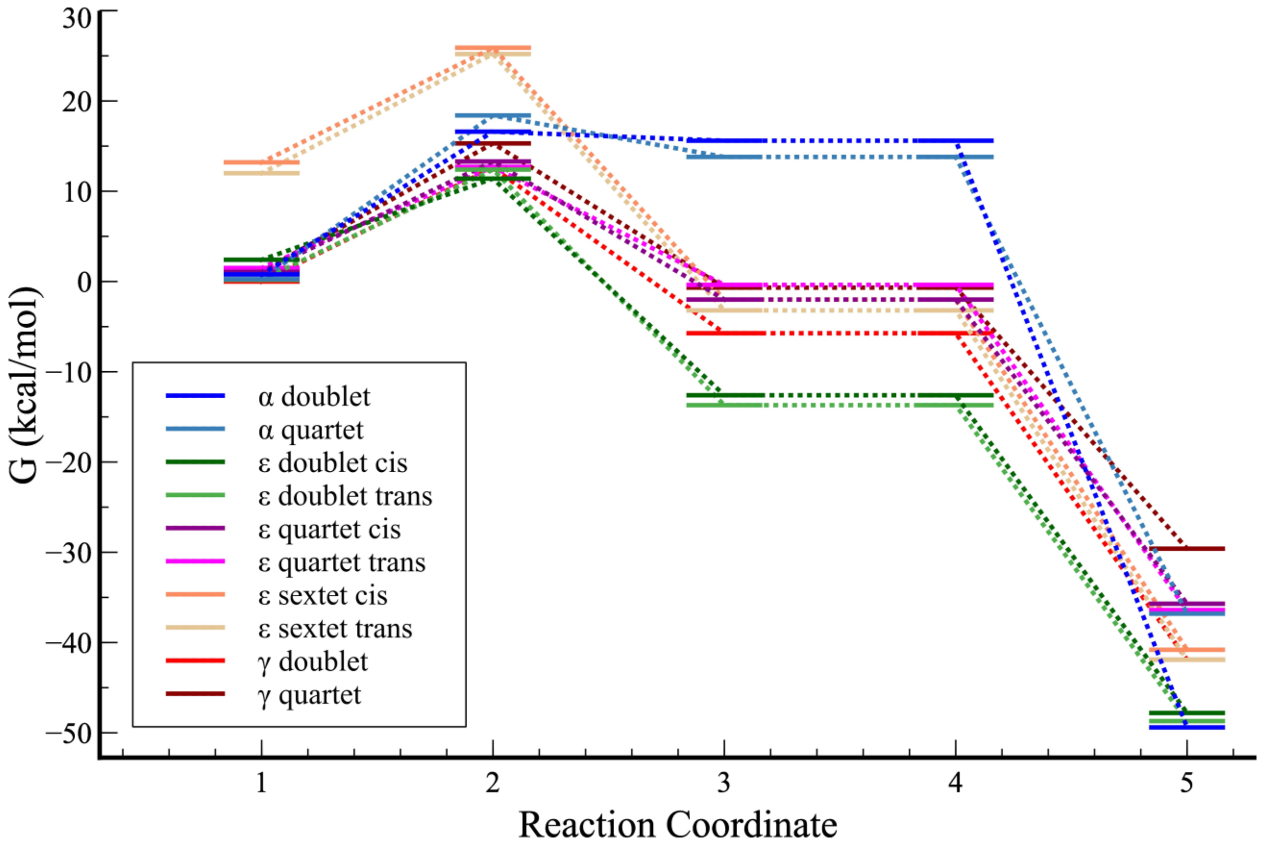

Table S12 and Figure S4: electronic analysis of α-pinene: Mulliken and NPA chargers and spin population in doublet and quartet states for epsilon cis and trans paths

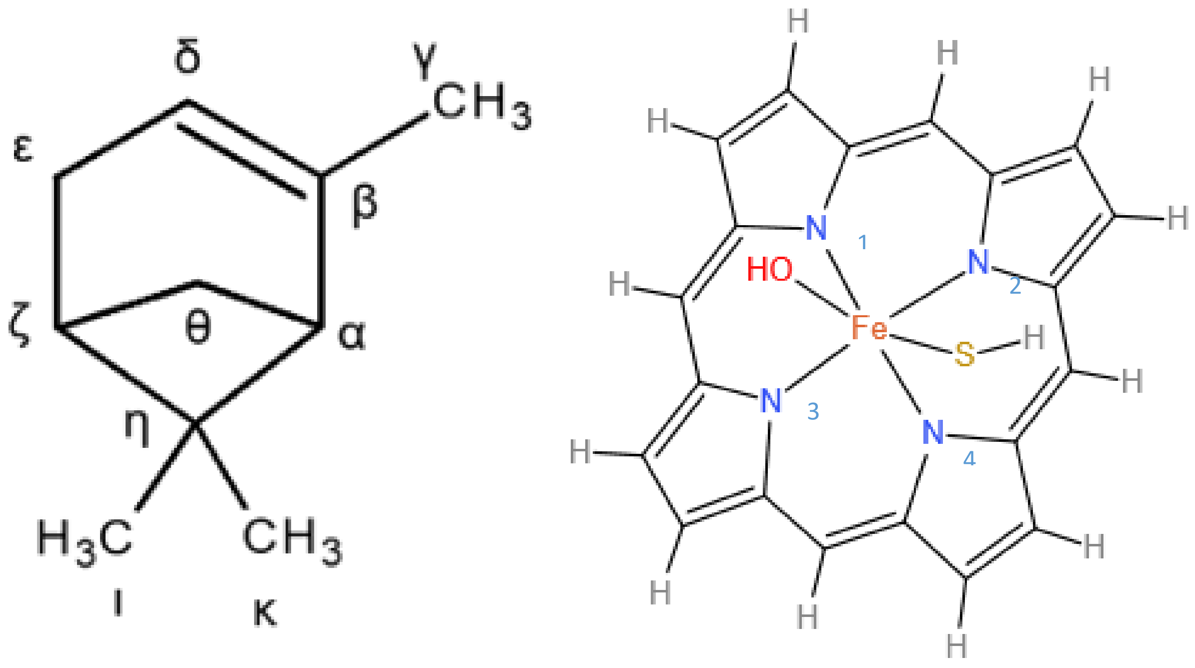

- Epsilon cis (doublet)

	Reactants				TS				Products			
	Mulliken	NPA	NBO spin Population	Mulliken spin Population	Mulliken	NPA	NBO spin Population	Mulliken spin Population	Mulliken	NPA	NBO spin Population	Mulliken spin Population
CYP												
Fe	0.445	25.695	1.194	1.295	0.370	25.690	1.028	1.093	0.318	25.630	0.960	0.991
N1	-0.516	7.394	-0.132	-0.138	-0.515	7.407	-0.108	-0.113	-0.519	7.451	-0.015	-0.015
N2	-0.513	7.394	-0.140	-0.147	-0.498	7.405	-0.114	-0.119	-0.514	7.440	-0.014	-0.016
N4	-0.515	7.395	-0.138	-0.144	-0.500	7.404	-0.112	-0.118	-0.515	7.439	-0.015	-0.015

N3	-0.513	7.393	-0.132	-0.138	-0.521	7.407	-0.012	-0.120	-0.526	7.456	-0.014	-0.016
S	-0.375	16.480	-0.253	-0.268	-0.344	16.453	-0.166	-0.178	-0.469	16.581	0.006	-0.001
O	-0.465	8.305	0.893	0.821	-0.565	8.459	0.596	0.561	-0.743	8.811	0.091	0.078
Alpha-pinene												
C - ε	-0.311	6.489	0.001	0.001	-0.335	6.398	0.182	0.209	0.013	5.922	0.000	0.000
C - ζ	-0.124	6.236	0.000	0.000	-0.129	6.246	-0.006	-0.009	-0.186	6.307	0.000	0.000
C - θ	-0.281	6.441	0.000	0.000	-0.280	6.424	0.018	0.019	-0.280	6.396	0.000	0.000
C - α	-0.191	6.262	0.000	0.000	-0.193	6.270	-0.005	-0.007	-0.209	6.297	0.000	0.000
C - η	0.141	6.044	0.000	0.000	0.135	6.036	0.005	0.005	0.113	5.975	0.000	0.000
C - β	0.219	6.024	0.000	0.000	0.223	5.958	0.071	0.082	0.280	5.709	0.000	0.000
C - δ	-0.226	6.241	0.000	0.000	-0.187	6.237	-0.051	-0.063	-0.175	6.321	0.000	0.000
H - δ	0.120	0.773	0.000	0.000	0.146	0.760	0.002	0.003	0.209	0.720	0.000	0.000
C - Y	-0.532	6.686	0.000	0.000	-0.534	6.695	-0.003	-0.006	-0.545	6.728	0.000	0.000
H3 – Y	0.154	0.763	0.000	0.000	0.165	0.757	0.000	0.000	0.209	0.731	0.000	0.000
H3 – Y	0.157	0.761	0.000	0.000	0.172	0.750	0.004	0.004	0.233	0.702	0.000	0.000
H3 – Y	0.156	0.762	0.000	0.000	0.170	0.752	0.002	0.003	0.228	0.706	0.000	0.000
H3 – Y ($\bar{x}$)	0.156	0.762	0.000	0.000	0.169	0.753	0.002	0.002	0.223	0.713	0.000	0.000
H - α	0.111	0.758	0.000	0.000	0.126	0.750	0.000	0.000	0.185	0.717	0.000	0.000
H - ζ	0.113	0.761	0.000	0.000	0.136	0.751	0.001	0.001	0.187	0.717	0.000	0.000
H trans - ε	0.140	0.757	0.000	0.000	0.179	0.736	-0.005	-0.005	0.227	0.724	0.000	0.000
H cis - ε	0.172	0.752	0.000	0.000	0.335	0.654	-0.028	-0.051	0.386	0.543	-0.002	-0.003
C - I	-0.481	6.678	0.000	0.000	-0.472	6.677	0.000	0.000	-0.503	6.700	0.000	0.000
H3 - I	0.146	0.770	0.000	0.000	0.151	0.766	0.000	0.000	0.175	0.748	0.000	0.000
H3 - I	0.137	0.770	0.000	0.000	0.138	0.770	0.000	0.000	0.172	0.750	0.000	0.000
H3 - I	0.157	0.763	0.000	0.000	0.160	0.762	0.000	0.000	0.185	0.750	0.000	0.001
H3 - I ($\bar{x}$)	0.147	0.768	0.000	0.000	0.150	0.766	0.000	0.000	0.177	0.749	0.000	0.000
C - K	-0.472	6.669	0.000	0.000	-0.473	6.670	0.000	0.000	-0.475	6.682	0.000	0.000
H3 - K	0.142	0.769	0.000	0.000	0.148	0.765	0.000	0.000	0.177	0.745	0.000	0.000
H3 - K	0.146	0.770	0.000	0.000	0.149	0.769	0.000	0.000	0.170	0.757	0.000	0.000
H3 - K	0.141	0.769	0.000	0.000	0.147	0.766	0.000	0.000	0.177	0.745	0.000	0.000

H3 - K ($\bar{x}$)	0.143	0.769	0.000	0.000	0.148	0.766	0.000	0.000	0.175	0.749	0.000	0.000
H2 - θ	0.139	0.766	0.000	0.000	0.152	0.759	0.000	0.000	0.198	0.733	0.000	0.000
H2 - θ	0.129	0.771	0.000	0.000	0.143	0.764	-0.001	-0.001	0.190	0.741	0.000	0.000
H2 - θ ($\bar{x}$)	0.134	0.768	0.000	0.000	0.148	0.762	-0.001	-0.001	0.194	0.737	0.000	0.000

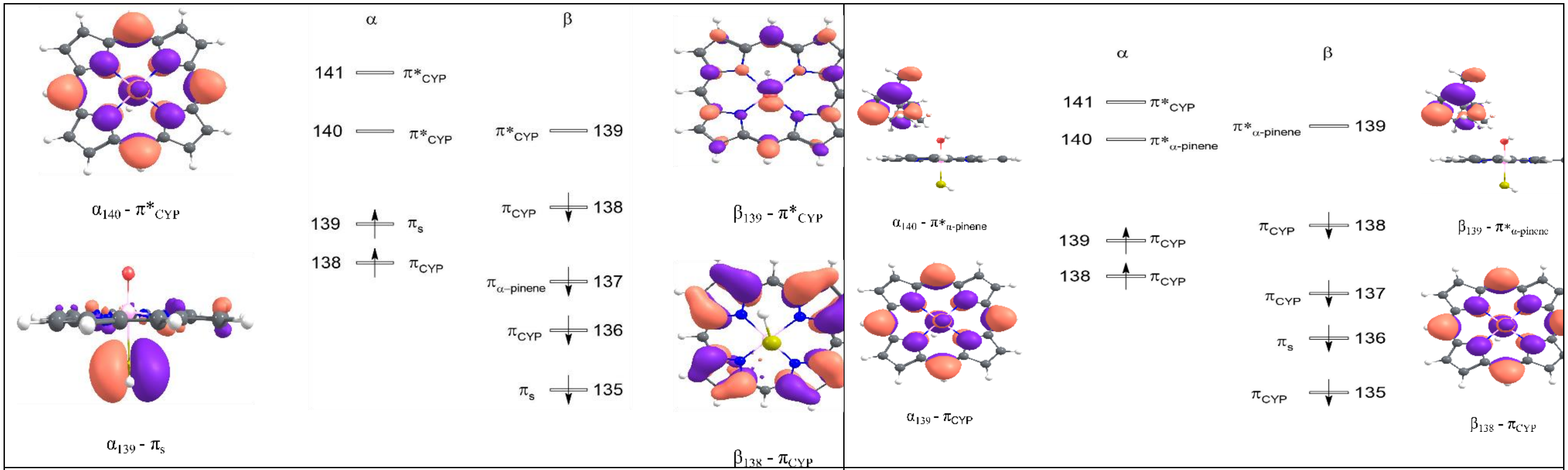

Reagent Complex	Product Complex

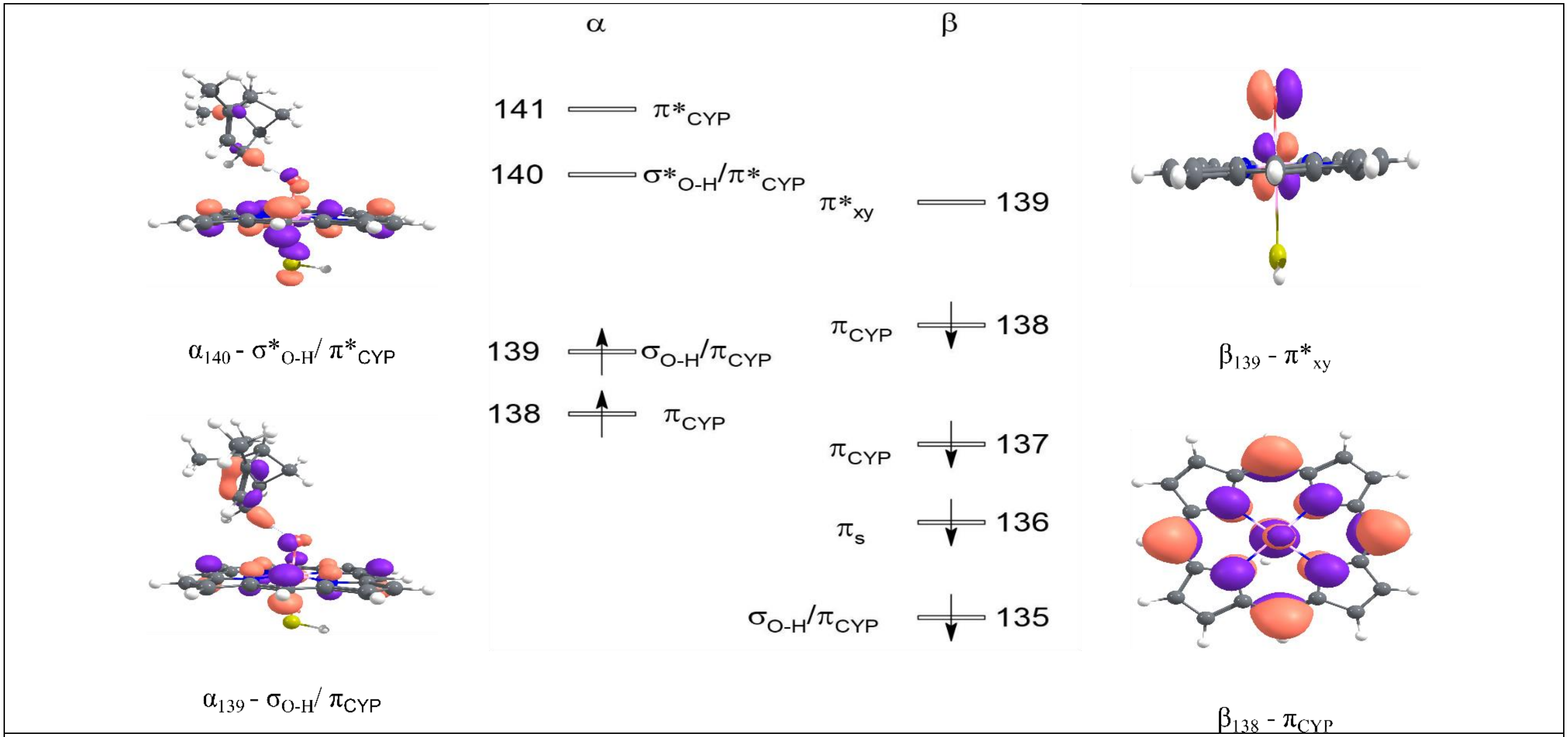

Transition State

- Epsilon cis (quartet)

	Reactants				TS				Products			
	Mulliken	NPA	NBO spin Population	Mulliken spin Population	Mulliken	NPA	NBO spin Population	Mulliken spin Population	Mulliken	NPA	NBO spin Population	Mulliken spin Population
CYP												
Fe	0.6163	25.2531	3.0360	3.2061	0.5229	25.4690	1.8600	1.9221	0.4247	25.5443	1.8770	1.9388

N1	-0.5659	7.4843	-0.0340	-0.0654	-0.5565	7.4434	-0.0250	-0.0251	-0.5631	7.4412	-0.0270	-0.0280
N2	-0.5544	7.4788	-0.0460	-0.0782	-0.5578	7.4368	-0.0250	-0.0254	-0.5494	7.4325	-0.0330	-0.0353
N4	-0.5686	7.4863	-0.0380	-0.0701	-0.5592	7.4392	-0.0290	-0.0294	-0.5629	7.4433	-0.0380	-0.0310
N3	-0.5548	7.4792	-0.0350	-0.0669	-0.5558	7.4421	-0.0230	-0.0227	-0.5612	7.4562	-0.0300	-0.0395
S	-0.3192	16.4458	-0.3060	-0.3266	-0.3315	16.4618	-0.0430	-0.0432	-0.1919	16.3053	-0.0060	-0.0182
O	-0.4936	8.3926	0.7220	0.6694	-0.5722	8.4842	0.6680	0.6681	-0.7063	8.7041	0.2460	0.2140
Alpha-pinene												
C - ε	-0.3116	6.4890	0.0010	0.0009	-0.3373	6.3847	0.3960	0.3961	-0.1468	6.2100	0.5460	0.6005
C - ζ	-0.1249	6.2363	0.0000	0.0000	-0.1351	6.2518	0.0180	-0.0176	-0.1649	6.2823	-0.1760	-0.0524
C - θ	-0.2816	6.4406	0.0000	0.0001	-0.2797	6.4226	0.0550	0.0554	-0.2774	6.4253	0.0610	0.0654
C - α	-0.1912	6.2625	0.0000	0.0000	-0.1936	6.2709	-0.0200	-0.0199	-0.1954	6.2751	-0.0290	-0.0510
C - η	0.1415	6.0437	0.0000	-0.0001	0.1346	6.0357	0.0220	0.0215	0.1397	6.0286	0.0600	0.0708
C - β	0.2197	6.0242	0.0000	0.0000	0.2265	5.9484	0.2670	0.2673	0.1830	5.9628	0.5240	0.5885
C - δ	-0.2269	6.2411	0.0000	-0.0001	-0.1770	6.2365	-0.1020	-0.1019	-0.1958	6.3053	-0.1760	-0.2421
H - δ	0.1211	0.7728	0.0000	-0.0001	0.1491	0.7575	0.0040	0.0039	0.1377	0.7584	0.0050	0.0088
C - Υ	-0.5317	6.6862	0.0000	0.0000	-0.5339	6.6970	-0.0200	-0.0200	-0.5339	6.7002	0.0270	-0.0505
H3 – Υ	0.1541	0.7628	0.0000	0.0000	0.1660	0.7559	0.0000	0.0003	0.1603	0.7594	0.0010	0.0011
H3 – Υ	0.1570	0.7610	0.0000	0.0000	0.1740	0.7480	0.0150	0.0153	0.1640	0.7561	0.0250	0.0278
H3 – Υ	0.1558	0.7620	0.0000	0.0000	0.1719	0.7503	0.0110	0.0107	0.1628	0.7568	0.0250	0.0283
H3 – Υ ($\bar{x}$)	0.1556	0.7619	0.0000	0.0000	0.1706	0.7514	0.0087	0.0088	0.1624	0.7575	0.0170	0.0190
H - α	0.1108	0.7582	0.0000	0.0000	0.1272	0.7494	0.0000	0.0000	0.1198	0.7532	0.0010	0.0008
H - ζ	0.1132	0.7608	0.0000	0.0000	0.1408	0.7489	0.0010	0.0010	0.1240	0.7509	0.0010	0.0009
H trans - ε	0.1409	0.7572	0.0000	0.0000	0.1875	0.7326	0.0010	-0.0020	0.1536	0.7646	-0.0170	-0.0303
H cis - ε	0.1709	0.7523	0.0000	0.0000	0.3622	0.6257	-0.0300	-0.0302	0.4360	0.5066	0.0050	0.0152
H2 - θ	0.1289	0.7706	0.0000	0.0000	0.1447	0.7632	-0.0020	-0.0026	0.1374	0.7658	-0.0020	-0.0034
H2 - θ	0.1395	0.7654	0.0000	0.0000	0.1538	0.7579	-0.0030	-0.0021	0.1480	0.7622	-0.0020	-0.0030
H2 - θ ($\bar{x}$)	0.1342	0.7680	0.0000	0.0000	0.1492	0.7606	-0.0025	-0.0023	0.1427	0.7640	-0.0020	-0.0032
C - I	-0.4719	6.6686	0.0000	0.0000	-0.4742	6.6702	-0.0010	-0.0010	-0.4722	6.6712	0.0030	-0.0054
H3 - I	0.1414	0.7692	0.0000	0.0000	0.1476	0.7650	0.0000	0.0004	0.1463	0.7660	0.0000	0.0028
H3 - I	0.1419	0.7688	0.0000	0.0000	0.1502	0.7648	0.0000	0.0011	0.1471	0.7654	0.0000	0.0028

H3 - I	0.1457	0.7704	0.0000	0.0000	0.1497	0.7685	0.0000	0.0000	0.1490	0.7687	0.0000	0.0001
H3 - I ($\bar{x}$)	0.1430	0.7694	0.0000	0.0000	0.1492	0.7661	0.0000	0.0005	0.1475	0.7667	0.0000	0.0019
C - K	-0.4775	6.6781	0.0000	0.0000	-0.4799	6.6792	-0.0010	-0.0010	-0.4808	6.6780	-0.0020	-0.0026
H3 - K	0.1431	0.7701	0.0000	0.0000	0.1554	0.7661	0.0000	-0.0001	0.1519	0.7674	0.0000	0.0000
H3 - K	0.1370	0.7702	0.0000	0.0000	0.1402	0.7682	0.0000	0.0004	0.1412	0.7682	0.0000	-0.0001
H3 - K	0.1547	0.7635	0.0000	0.0000	0.1601	0.7611	0.0000	-0.0007	0.1557	0.7654	0.0000	-0.0008
H3 - K ($\bar{x}$)	0.1449	0.7679	0.0000	0.0000	0.1519	0.7651	0.0000	-0.0001	0.1496	0.7670	0.0000	-0.0003

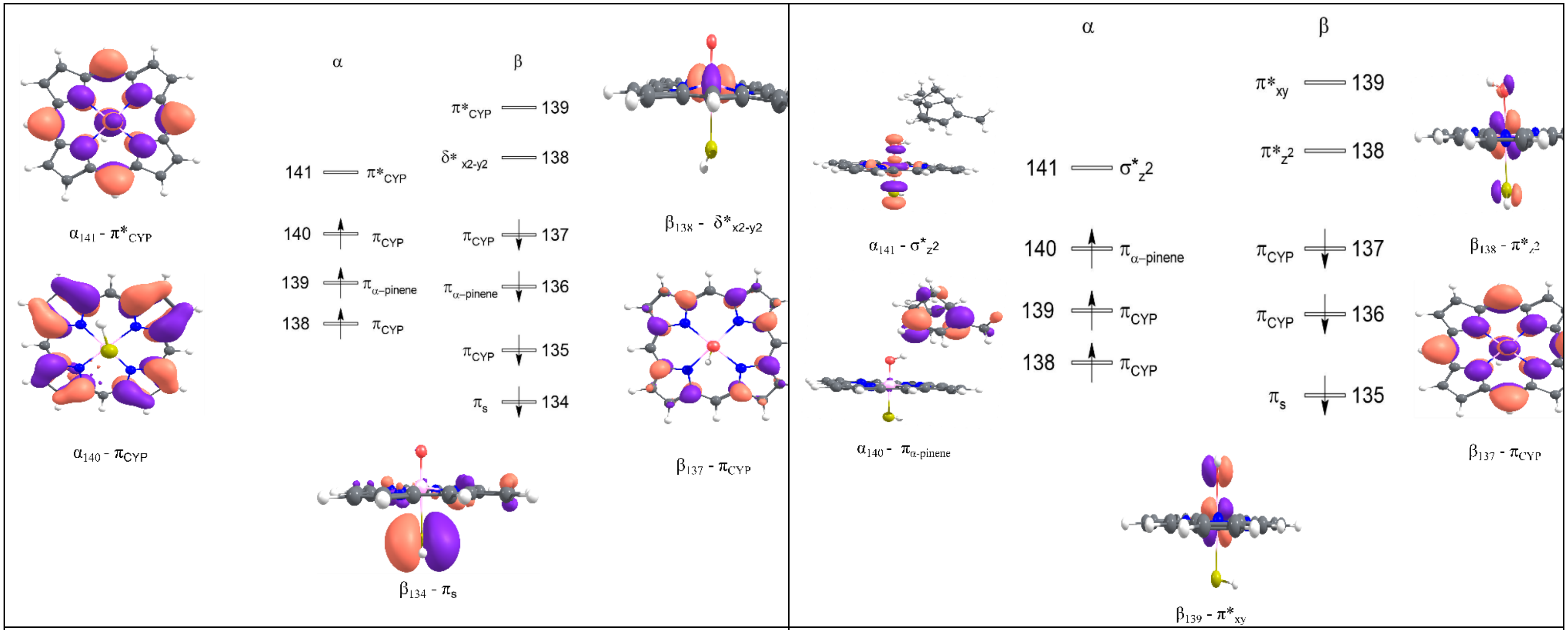

Reagent Complex	Product Complex

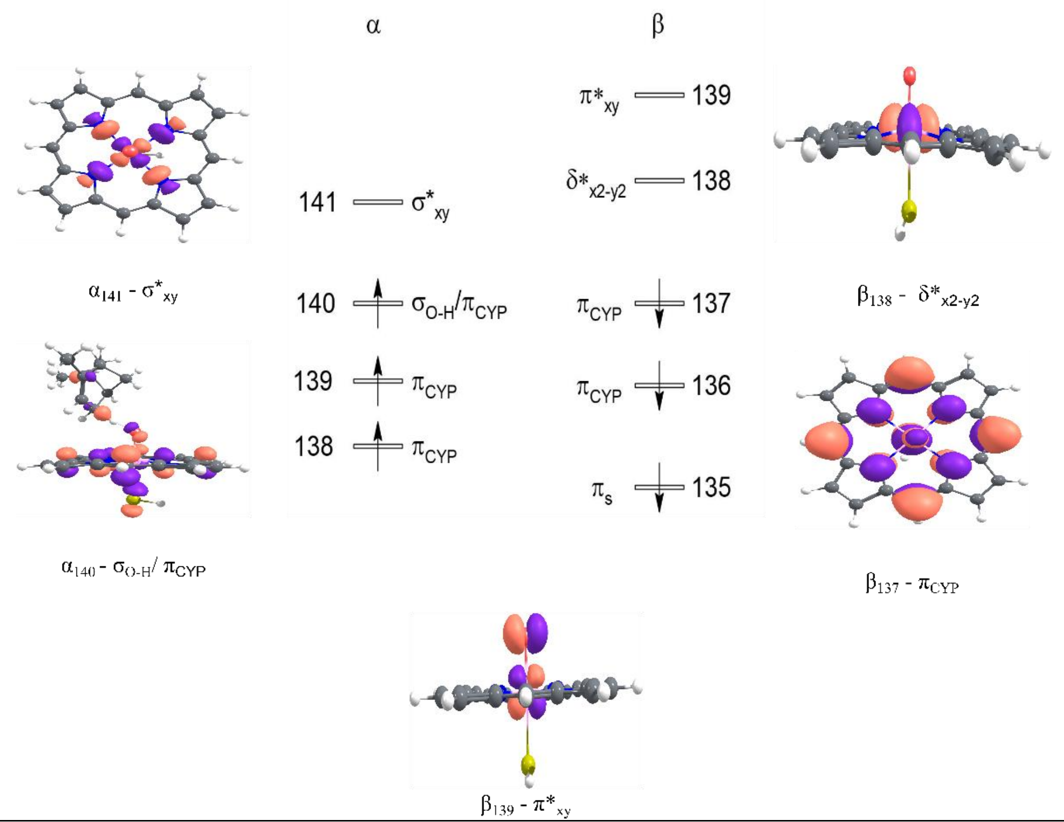

Transition State

- Epsilon trans (doublet)

	Reactants				TS				Products			
	Mulliken	NPA	NBO spin Population	Mulliken spin Population	Mulliken	NPA	NBO spin Population	Mulliken spin Population	Mulliken	NPA	NBO spin Population	Mulliken spin Population
CYP												
Fe	0.451	25.696	1.194	1.294	0.433	25.641	1.452	1.545	0.343	25.623	0.969	1.000

N1	-0.517	7.395	-0.137	-0.143	-0.539	7.427	-0.044	-0.047	-0.523	7.454	-0.015	-0.017
N2	-0.512	7.393	-0.141	-0.148	-0.511	7.419	-0.065	-0.068	-0.516	7.441	-0.015	-0.016
N4	-0.516	7.394	-0.132	-0.139	-0.531	7.426	-0.048	-0.051	-0.515	7.440	-0.015	-0.015
N3	-0.513	7.393	-0.131	-0.138	-0.543	7.426	-0.052	-0.054	-0.524	7.454	-0.015	-0.017
S	-0.376	16.478	-0.252	-0.266	-0.328	16.443	-0.131	-0.143	-0.466	16.574	0.005	-0.003
O	-0.467	8.306	0.893	0.820	-0.551	8.415	0.367	0.312	-0.737	8.819	0.084	0.072
Alpha-pinene												
C - ε	-0.314	6.489	0.001	0.002	-0.361	6.439	-0.137	-0.140	0.013	5.928	0.000	0.000
C - ζ	-0.130	6.236	0.000	0.000	-0.122	6.242	0.001	0.005	-0.191	6.308	0.000	0.000
C - η	0.142	6.044	0.000	0.000	0.134	6.022	-0.037	-0.043	0.117	5.974	0.000	0.000
C - α	-0.192	6.262	0.000	0.000	-0.196	6.273	0.004	0.013	-0.211	6.299	0.000	0.000
C - θ	-0.285	6.441	0.000	0.000	-0.291	6.433	-0.016	-0.017	-0.314	6.399	0.001	0.001
H2 - θ	0.135	0.771	0.000	0.000	0.158	0.762	0.000	0.001	0.180	0.749	0.000	0.000
H2 - θ	0.140	0.765	0.000	0.000	0.158	0.755	0.001	0.000	0.232	0.718	0.000	0.001
H2 - θ ($\bar{x}$)	0.137	0.768	0.000	0.000	0.105	0.505	0.000	0.000	0.206	0.733	0.000	0.000
C - β	0.220	6.024	0.000	0.000	0.231	5.924	-0.189	-0.211	0.278	5.710	0.000	0.000
C - δ	-0.220	6.241	0.000	0.000	-0.163	6.198	-0.023	-0.009	-0.165	6.320	0.000	0.000
H - δ	0.118	0.773	0.000	0.000	0.160	0.753	0.001	0.001	0.205	0.719	0.000	0.000
C - Y	-0.532	6.686	0.000	0.000	-0.534	6.701	0.006	0.015	-0.543	6.728	0.000	0.000
H3 – Y	0.154	0.763	0.000	0.000	0.172	0.753	-0.009	0.000	0.205	0.732	0.000	0.000
H3 – Y	0.156	0.762	0.000	0.000	0.181	0.743	-0.011	-0.014	0.228	0.707	0.000	0.000
H3 – Y	0.157	0.761	0.000	0.000	0.180	0.744	0.000	-0.010	0.230	0.704	0.000	0.000
H3 – Y ($\bar{x}$)	0.156	0.762	0.000	0.000	0.178	0.747	-0.007	-0.008	0.221	0.714	0.000	0.000
H - α	0.111	0.758	0.000	0.000	0.135	0.745	0.000	0.000	0.181	0.721	0.000	0.000
C - I	-0.472	6.669	0.000	0.000	-0.473	6.673	0.001	0.003	-0.512	6.694	0.000	0.000
H3 - I	0.141	0.769	0.000	0.000	0.152	0.762	0.000	-0.002	0.167	0.752	0.000	0.000
H3 - I	0.142	0.769	0.000	0.000	0.153	0.761	0.000	-0.002	0.170	0.751	0.000	0.000
H3 - I	0.146	0.770	0.000	0.000	0.154	0.766	0.001	0.000	0.214	0.738	0.000	-0.001
H3 - I ($\bar{x}$)	0.143	0.770	0.000	0.000	0.153	0.763	0.000	-0.002	0.184	0.747	0.000	0.000
C - K	-0.471	6.678	0.000	0.000	-0.473	6.681	0.001	0.002	-0.475	6.691	0.000	0.000

H3 - K	0.138	0.770	0.000	0.000	0.150	0.762	0.002	0.000	0.173	0.747	0.000	0.000
H3 - K	0.137	0.770	0.000	0.000	0.149	0.763	0.000	-0.001	0.172	0.748	0.000	0.000
H3 - K	0.148	0.763	0.000	0.000	0.153	0.763	-0.002	0.000	0.159	0.760	0.000	0.000
H3 - K ($\bar{x}$)	0.141	0.768	0.000	0.000	0.151	0.763	0.000	0.000	0.168	0.752	0.000	0.000
H - ζ	0.116	0.761	0.000	0.000	0.143	0.747	0.000	0.000	0.187	0.718	0.000	0.000
H cis - ε	0.172	0.749	0.000	-0.001	0.188	0.726	-0.007	-0.007	0.228	0.724	0.000	0.000
H trans - ε	0.142	0.760	0.001	0.000	0.330	0.650	-0.020	-0.024	0.391	0.537	-0.002	-0.004

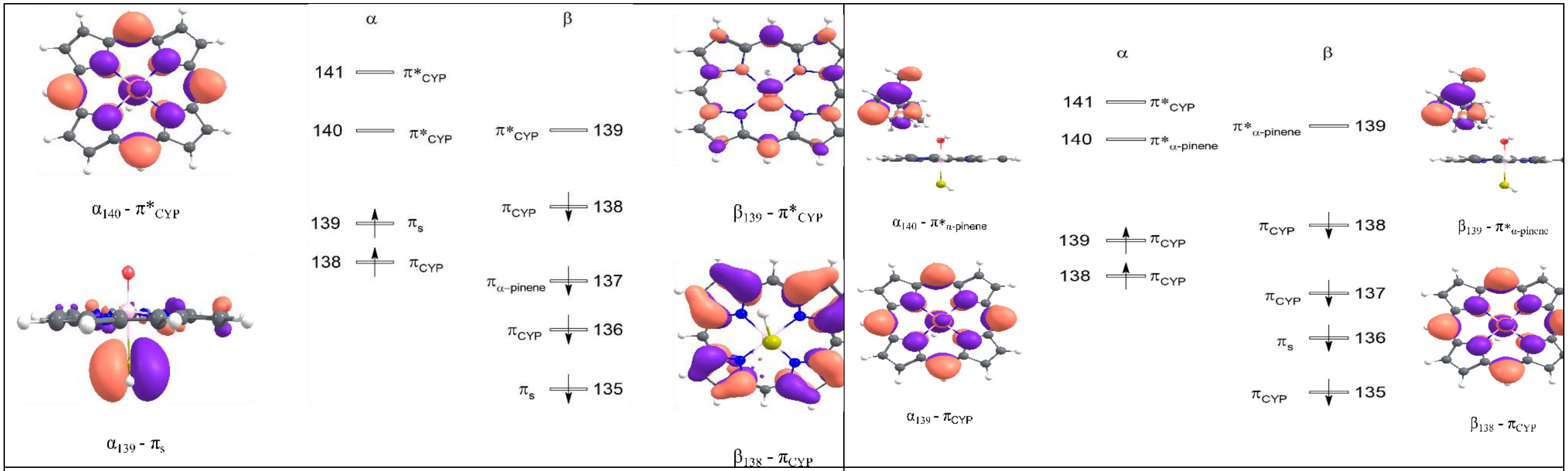

Reagent Complex | Product Complex

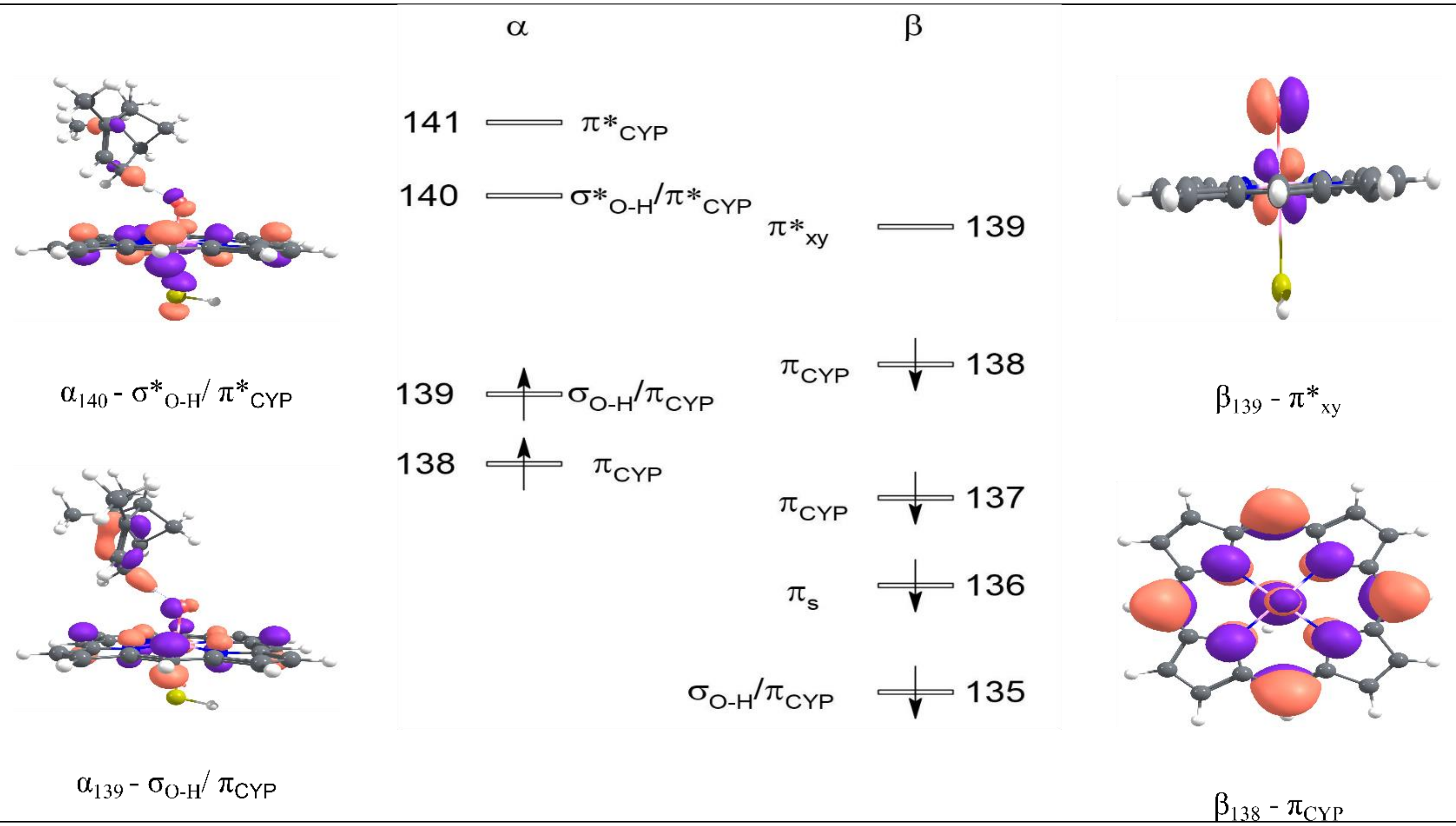

Transition State

- Epsilon trans (quartet)

	Reactants				TS				Products			
	Mulliken	NPA	NBO spin Population	Mulliken spin Population	Mulliken	NPA	NBO spin Population	Mulliken spin Population	Mulliken	NPA	NBO spin Population	Mulliken spin Population
CYP												
Fe	0.6197	25.2538	3.0360	3.2058	0.5178	25.4718	1.8600	1.9213	0.4340	25.5623	1.8350	1.8956

N1	-0.5723	7.4883	-0.0380	-0.0694	-0.5557	7.4422	-0.0210	-0.0239	-0.5665	7.4400	-0.0250	-0.0269
N2	-0.5506	7.4766	-0.0470	-0.0785	-0.5594	7.4390	0.0300	-0.0292	-0.5518	7.4294	-0.0310	-0.0325
N4	-0.5699	7.4864	-0.0310	-0.0632	-0.5590	7.4371	-0.0260	-0.0265	-0.5651	7.4422	-0.0360	-0.0304
N3	-0.5515	7.4778	-0.0380	-0.0697	-0.5587	7.4433	-0.0230	-0.0251	-0.5659	7.4592	-0.0390	-0.0401
S	-0.3234	16.4464	-0.3250	-0.3247	-0.3331	16.4602	-0.0370	-0.0447	-0.1649	16.2713	0.0070	-0.0056
O	-0.4932	8.3935	0.6690	0.6691	-0.5684	8.4811	0.7100	0.6770	-0.6962	8.7065	0.2370	0.2075
Alpha-pinene												
C - ε	-0.3131	6.4893	0.0010	0.0010	-0.3408	6.3902	0.3560	0.3896	-0.1282	6.1944	0.5780	0.6438
C - ζ	-0.1304	6.2364	0.0000	0.0000	-0.1238	6.2506	-0.0120	-0.0200	-0.1867	6.2853	-0.0330	-0.0549
C - η	0.1422	6.0439	0.0000	0.0000	0.1376	6.0255	0.0520	0.0602	0.1439	6.0291	0.065	0.0768
C - α	-0.1915	6.2624	0.0000	0.0000	-0.1941	6.2707	-0.0100	-0.0198	-0.1961	6.2737	-0.030	-0.0527
C - θ	-0.2846	6.4411	0.0000	0.0000	-0.2902	6.4344	0.0190	0.0199	-0.2759	6.4275	0.0610	0.0652
H2 - θ	0.1349	0.7706	0.0000	0.0000	0.1544	0.7633	-0.0010	-0.0011	0.1326	0.7681	-0.0020	-0.0034
H2 - θ	0.1397	0.7654	0.0000	0.0000	0.1528	0.7578	0.0000	-0.0005	0.1441	0.7648	-0.0020	-0.0031
H2 - θ ($\bar{x}$)	0.1373	0.7680	0.0000	0.0000	0.1536	0.7605	-0.0005	-0.0008	0.1383	0.7665	-0.0020	-0.0032
C - β	0.2195	6.0240	0.0000	0.0002	0.2270	5.9478	0.2300	0.2615	0.1788	5.9785	0.5380	0.6048
C - δ	-0.2205	6.2415	0.0000	-0.0001	-0.1797	6.2362	-0.0650	-0.0940	-0.1905	6.3065	-0.1870	-0.2570
H - δ	0.1177	0.7727	0.0000	0.0000	0.1490	0.7570	0.0020	0.0032	0.1229	0.7633	0.0050	0.0093
C - Y	-0.5316	6.6862	0.0000	0.0000	-0.5338	6.6970	-0.0090	-0.0196	-0.5321	6.6982	-0.0280	-0.0523
H3 – Y	0.1541	0.7627	0.0000	0.0000	0.1663	0.7560	0.0000	0.0003	0.1566	0.7612	0.0010	0.0011
H3 – Y	0.1562	0.7618	0.0000	0.0000	0.1736	0.7485	0.0130	0.0152	0.1587	0.7602	0.0260	0.0292
H3 – Y	0.1569	0.7610	0.0000	0.0000	0.1730	0.7494	0.0090	0.0105	0.1595	0.7594	0.0250	0.0284
H3 – Y ($\bar{x}$)	0.1557	0.7618	0.0000	0.0000	0.1710	0.7513	0.0073	0.0087	0.1582	0.7602	0.0173	0.0196
H - α	0.1108	0.7582	0.0000	0.0000	0.1281	0.7487	0.0000	0.0002	0.1148	0.7561	-0.0300	0.0009
C - I	-0.4719	6.6686	0.0000	0.0000	-0.4729	6.6717	-0.0020	-0.0037	-0.4770	6.6714	-0.0020	-0.0060
H3 - I	0.1414	0.7692	0.0000	0.0000	0.1500	0.7632	0.0030	0.0036	0.1436	0.7674	0.0000	0.0031
H3 - I	0.1415	0.7690	0.0000	0.0000	0.1503	0.7632	0.0020	0.0022	0.1503	0.7674	0.0000	0.0030
H3 - I	0.1456	0.7704	0.0000	0.0000	0.1518	0.7670	0.0000	0.0000	0.1483	0.7694	0.0000	0.0001
H3 - I ($\bar{x}$)	0.1428	0.7695	0.0000	0.0000	0.1507	0.7645	0.0025	0.0019	0.1474	0.7681	0.0000	0.0021
C - K	-0.4713	6.6775	0.0000	0.0000	-0.4721	6.6796	-0.0020	-0.0033	-0.4732	6.6769	-0.0040	-0.0032

H3 - K	0.1381	0.7701	0.0000	0.0000	0.1467	0.7646	0.0000	0.0002	0.1413	0.7694	0.0030	0.0000
H3 - K	0.1370	0.7701	0.0000	0.0000	0.1455	0.7648	0.0010	0.0004	0.1361	0.7711	0.0030	-0.0001
H3 - K	0.1479	0.7635	0.0000	0.0000	0.1516	0.7629	0.0000	-0.0004	0.1480	0.7618	0.0000	-0.0010
H3 - K ($\bar{x}$)	0.1410	0.7679	0.0000	0.0000	0.1479	0.7641	0.0003	0.0000	0.1418	0.7675	0.0020	-0.0004
H - ζ	0.1169	0.7607	0.0000	0.0000	0.1385	0.7491	0.0020	0.0017	0.1343	0.7548	0.0010	0.0011
H cis - ε	0.1712	0.7488	0.0000	-0.0001	0.1842	0.7345	-0.0010	-0.0025	0.1347	0.7714	-0.0180	-0.0327
H trans - ε	0.1421	0.7601	0.0000	0.0000	0.3589	0.6276	-0.0160	-0.0332	0.4369	0.5050	0.0010	0.0034

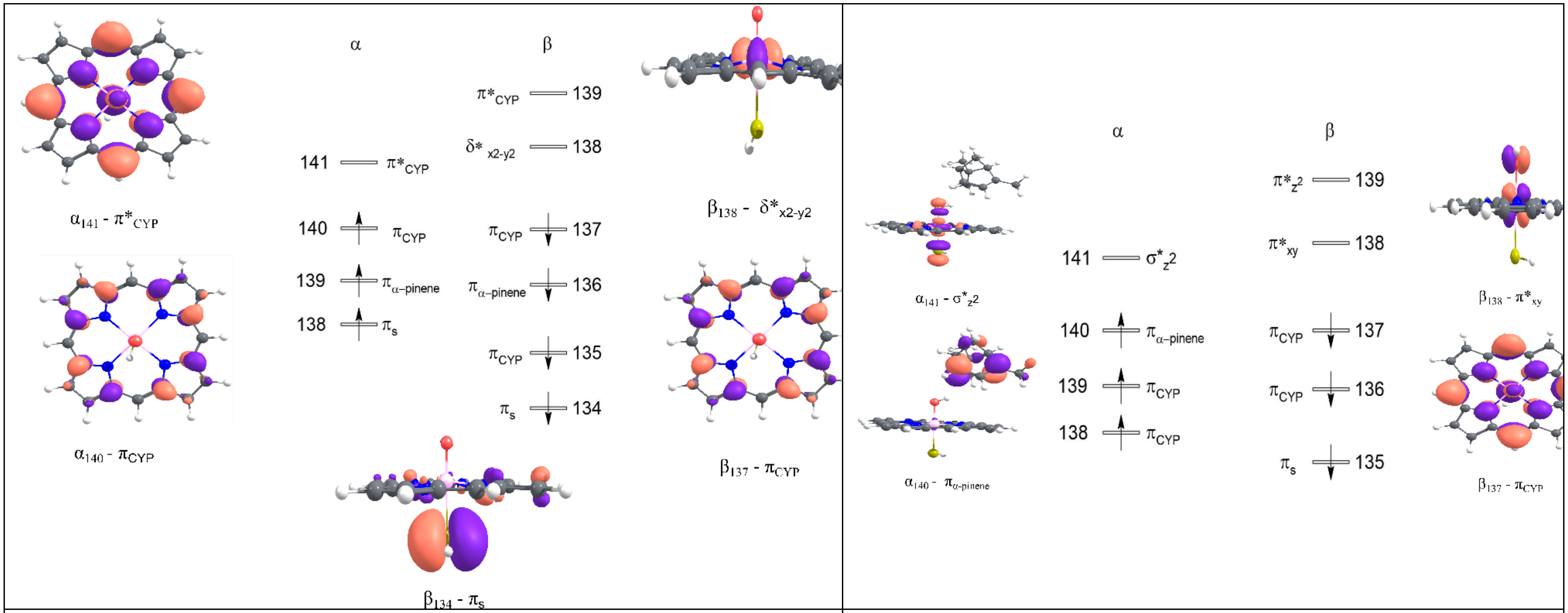

Reagent Complex	Product Complex

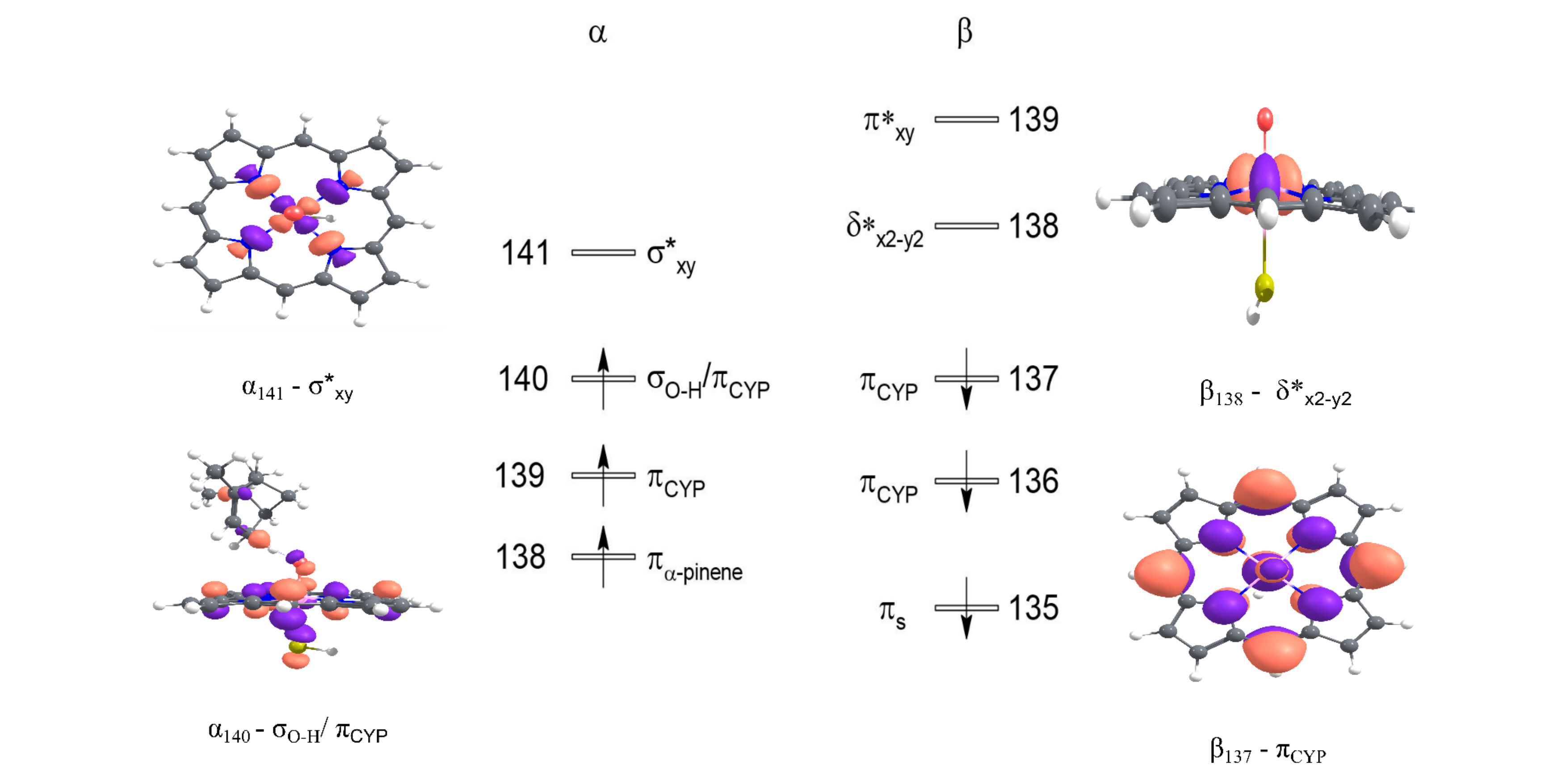

Transition State

Table S13: Coordinates Beta-Pinene

Alpha site – Doublet state

Coordinate 1

Fe	1.45571400	0.01158500	0.17518400
N	1.90242500	1.91962000	-0.30792600
C	2.75612700	2.34091300	-1.29634500
C	2.73440400	3.77923900	-1.40491400
C	1.84654300	4.22696200	-0.47475400
C	1.33157600	3.06016600	0.19916600
C	0.39212600	3.09372700	1.22481200
C	-0.10145900	1.98604700	1.90657100
C	-1.05138700	2.04949900	2.99103400
C	-1.26799700	0.76671200	3.39406800
C	-0.45087000	-0.07377100	2.55229200
N	0.25742000	0.68600800	1.65587200
C	-0.39276700	-1.46019200	2.64503400
C	0.39010400	-2.29568400	1.85447500
C	0.43390100	-3.73172000	1.98321900
C	1.30211500	-4.18133900	1.03535700
C	1.78432000	-3.01725400	0.33307700
N	1.22224700	-1.87680500	0.84759900
C	2.69794000	-3.05294800	-0.71595300
C	3.19388500	-1.94457700	-1.39512400
C	4.16326200	-2.00693700	-2.46231000
C	4.39922100	-0.72281900	-2.84831600
C	3.57231900	0.11633900	-2.01490600
C	3.53049800	1.50462200	-2.09387400
H	4.15975600	1.97716900	-2.84144500

N	2.84644700	-0.64428000	-1.13493100
H	5.06684200	-0.35847500	-3.61797600
H	4.59661200	-2.92062300	-2.84722000
H	3.06155700	-4.02800000	-1.02448400
H	1.60164000	-5.19836600	0.81916600
H	-0.13041000	-4.30171900	2.70947300
H	-1.01010900	-1.93191400	3.40296600
H	-1.91094500	0.40498600	4.18563500
H	-1.47870800	2.96343400	3.38195600
H	0.02170200	4.06834000	1.52647100
H	1.55649200	5.24489700	-0.25011000
H	3.32688400	4.35193900	-2.10617300
O	0.23481000	-0.09143900	-0.91030400
S	3.35180400	0.16476500	1.87892800
H	3.20019800	1.46428500	2.21678600
C	-6.33194400	-0.74861600	-0.80700900
C	-5.55464300	0.30665800	-0.00092100
C	-4.37526800	-0.38387500	0.74022300
C	-3.57367500	-0.22365800	-0.58920200
C	-4.48745900	1.03143000	-0.89566800
C	-4.82553500	1.35056800	-2.35440800
H	-5.60334300	2.12360100	-2.40853400
H	-3.93956300	1.73942200	-2.87314800
H	-5.17537100	0.48186900	-2.91788400
C	-3.94527800	2.30904800	-0.23531700
H	-4.69882000	3.10648100	-0.27450400
H	-3.66657500	2.16891400	0.81331800
H	-3.05546900	2.66774600	-0.76861700
C	-3.93247500	-1.34957400	-1.52723800
C	-5.37608300	-1.82809200	-1.39574900
H	-5.74681300	-2.20262800	-2.35628400

H	-5.37698300	-2.69307000	-0.71900200
C	-3.05989900	-1.88618400	-2.38795500
H	-3.34609200	-2.67866800	-3.07700000
H	-2.02513000	-1.55223600	-2.42617200
H	-2.49214500	-0.05595600	-0.54253100
H	-4.53673100	-1.40056700	1.11447500
H	-3.96131500	0.22096900	1.54924000
H	-6.23205000	0.94617800	0.57853900
H	-6.90112000	-0.25800700	-1.60544800
H	-7.06926200	-1.23588700	-0.15719100

Coordinate II – TS (-1655.11)

Fe	-1.08984500	0.02570200	0.34743800
N	-0.64993500	-1.81826200	1.01892300
C	-1.12433600	-3.00239600	0.50720900
C	-0.56675600	-4.11945600	1.23267200
C	0.24515300	-3.59772800	2.19419000
C	0.18664800	-2.16290400	2.04879000
C	0.88279700	-1.25425600	2.84193400
C	0.84574400	0.12852100	2.69931700
C	1.56915400	1.05817000	3.53349000
C	1.27267900	2.30549200	3.07540400
C	0.36203000	2.13619100	1.96750600
N	0.12051300	0.80101400	1.74405500
C	-0.20599400	3.18694500	1.25503700
C	-1.14585600	3.06916100	0.23369900
C	-1.74312000	4.18556800	-0.46195900
C	-2.61620100	3.65828100	-1.36471100
C	-2.54352600	2.22270300	-1.21993600
N	-1.64640600	1.88653400	-0.23909200
C	-3.27500400	1.31607100	-1.98211600

C	-3.19720100	-0.07024000	-1.88706300
C	-3.93910500	-0.99604900	-2.71328300
C	-3.57795700	-2.24701700	-2.31555300
C	-2.62155600	-2.07960000	-1.24388900
C	-2.02444500	-3.12632700	-0.54604100
H	-2.29791800	-4.13492100	-0.84088900
N	-2.40339200	-0.75025100	-1.00099500
H	-3.91788200	-3.20170800	-2.69570900
H	-4.63641000	-0.70807600	-3.48936400
H	-3.95174600	1.72615200	-2.72548000
H	-3.25295000	4.17518500	-2.07096800
H	-1.51401700	5.22585600	-0.27023100
H	0.09239500	4.19178600	1.53781600
H	1.61984600	3.26144000	3.44565400
H	2.21181500	0.77531100	4.35708100
H	1.50413300	-1.65770400	3.63534900
H	0.83467500	-4.11887400	2.93725200
H	-0.78310000	-5.15894800	1.02256300
O	0.07385700	-0.03434300	-0.96990600
S	-2.96511200	0.03854100	1.91399000
H	-2.26354200	-0.14671900	3.05461800
C	5.31383900	0.21360500	-1.03746000
C	4.39691900	-0.97984400	-0.71731900
C	3.38211000	-0.56697300	0.39439500
C	2.57131200	0.00152500	-0.78136600
C	3.18550300	-1.08571800	-1.73060200
C	3.39802100	-0.73385900	-3.20177900
H	4.02630400	-1.49285100	-3.68450900
H	2.43592200	-0.71866300	-3.72878800
H	3.86694200	0.24127800	-3.35075100
C	2.49414200	-2.45455700	-1.64244800

H	3.11889500	-3.21725800	-2.12335900
H	2.30517900	-2.77885600	-0.61558500
H	1.53229200	-2.42723600	-2.16620900
C	3.03525600	1.37368100	-1.16347800
C	4.53727900	1.55542600	-0.92141200
H	4.93846200	2.31219200	-1.60366200
H	4.67371800	1.95332400	0.09228300
C	2.25314000	2.32166100	-1.69179500
H	2.65966500	3.28311100	-1.99757200
H	1.18650900	2.16490100	-1.81661000
H	3.71307900	0.13164300	1.16613400
H	2.91289600	-1.42845800	0.87078200
H	4.95527300	-1.91125700	-0.55972500
H	5.73997000	0.09532900	-2.04000200
H	6.15762600	0.21991700	-0.33769400
H	1.26939900	-0.03576200	-0.72924100

Coordinate III

Fe	-1.23600300	0.04435300	0.30908100
N	-0.65529200	-1.74535500	1.05959000
C	-1.02106900	-2.97850600	0.57509600
C	-0.37642300	-4.02360000	1.33477700
C	0.38255400	-3.41115800	2.28454500
C	0.21110600	-1.98993200	2.09863100
C	0.85003400	-1.00587700	2.84303000
C	0.72778000	0.36632300	2.63860300
C	1.41074700	1.37565900	3.41200200
C	1.02396600	2.57967200	2.90723500
C	0.10189400	2.30101500	1.83190000
N	-0.05950000	0.94860200	1.67778200
C	-0.55258600	3.27711700	1.08635000

C	-1.48791000	3.05286900	0.08146200
C	-2.16319500	4.09920200	-0.65223100
C	-3.00161100	3.47996600	-1.52713400
C	-2.82945000	2.05937600	-1.32603100
N	-1.91028100	1.82323000	-0.34420100
C	-3.50115300	1.07428600	-2.04714800
C	-3.32259700	-0.29487600	-1.90127700
C	-3.98948300	-1.30456700	-2.69341400
C	-3.52025700	-2.50592400	-2.26365100
C	-2.57602900	-2.22707300	-1.20300600
C	-1.89163300	-3.20145300	-0.48437300
H	-2.07638200	-4.23620800	-0.75830100
N	-2.47308800	-0.87854400	-0.99947000
H	-3.78053900	-3.49667900	-2.61395200
H	-4.71337600	-1.10128200	-3.47224700
H	-4.20902200	1.40701400	-2.80073200
H	-3.67573700	3.92554300	-2.24751000
H	-2.00595200	5.15986400	-0.50292900
H	-0.32179800	4.31208500	1.32187400
H	1.31898100	3.57113700	3.22660000
H	2.08914400	1.17345500	4.23118100
H	1.50638700	-1.33350300	3.64410400
H	1.00712600	-3.86422700	3.04407400
H	-0.50361000	-5.08358900	1.15502400
O	0.05148100	0.01060000	-0.95656500
S	-3.08625200	-0.21895300	1.86404700
H	-2.37212500	-0.36833200	3.00296700
C	5.67120100	0.25558100	-1.16935400
C	4.80096900	-0.91748300	-0.66956400
C	3.93597100	-0.42746000	0.53754900
C	3.02912700	0.07893500	-0.57953800

C	3.46285000	-1.07375400	-1.51652600
C	3.48060300	-0.81793000	-3.02159200
H	4.00077900	-1.63239200	-3.54166300
H	2.45245700	-0.78038700	-3.40005800
H	3.95911800	0.12520100	-3.29436600
C	2.76177100	-2.41589400	-1.24830000
H	3.27790400	-3.22323300	-1.78306100
H	2.72824000	-2.68534500	-0.18915100
H	1.72871100	-2.36823100	-1.60879200
C	3.39870000	1.41881800	-1.09053700
C	4.92072100	1.61478600	-1.02829100
H	5.24436000	2.33139300	-1.79108100
H	5.17873600	2.06100400	-0.05894700
C	2.54013800	2.30479600	-1.60703000
H	2.88514200	3.24986400	-2.02160900
H	1.47291600	2.10376700	-1.62028600
H	4.36709600	0.30899900	1.22021600
H	3.51216800	-1.25144300	1.11450200
H	5.37895400	-1.84245500	-0.53127200
H	5.96283400	0.08073200	-2.21119900
H	6.60081800	0.28880900	-0.58828200
H	0.91765500	-0.05932900	-0.50244700

Coordinate V

Fe	-1.12882000	0.03680500	0.37771900
N	-1.94098600	-0.25740500	-1.44811800
C	-2.36510200	0.69135300	-2.35340800
C	-2.97964600	0.05679000	-3.49261400
C	-2.93908900	-1.28594300	-3.26374600
C	-2.29822200	-1.47391800	-1.98493300
C	-2.08506200	-2.71263300	-1.39400800

C	-1.47958100	-2.92414700	-0.16158000
C	-1.25680100	-4.22907500	0.41561600
C	-0.63512500	-4.03057800	1.61111000
C	-0.47882400	-2.60434000	1.76528900
N	-0.99617700	-1.94566800	0.67437500
C	0.10817900	-1.99151000	2.86484800
C	0.25640300	-0.61913200	3.01496600
C	0.85441000	0.01545400	4.16360100
C	0.79879200	1.35858400	3.94306900
C	0.16480700	1.54575800	2.65980800
N	-0.14842000	0.32805000	2.10158100
C	-0.08834200	2.78755400	2.09153900
C	-0.70044900	2.99981500	0.86306300
C	-0.94636300	4.30466900	0.29646200
C	-1.54807300	4.10380700	-0.90873000
C	-1.67075000	2.67626800	-1.07845200
C	-2.23770900	2.06452500	-2.18913800
H	-2.61403500	2.70448300	-2.98076000
N	-1.14573400	2.01793900	0.01043000
H	-1.88132700	4.83951400	-1.62955300
H	-0.68209400	5.24070600	0.77195200
H	0.21702800	3.66402600	2.65444800
H	1.14774200	2.16262300	4.57851200
H	1.25881200	-0.51345700	5.01720300
H	0.47243200	-2.62915800	3.66400800
H	-0.30428100	-4.76718400	2.33211800
H	-1.54159400	-5.16366800	-0.05077900
H	-2.41781300	-3.58875100	-1.94179100
H	-3.30468600	-2.08876600	-3.89135800
H	-3.38517200	0.58494300	-4.34614000
O	0.82827700	-0.11641700	-0.84216000

S	-3.16823700	0.14288900	1.37484000
H	-2.90794600	-0.65101200	2.43710900
C	5.05459500	0.17618600	-0.48952800
C	4.08836200	-1.00700900	-0.33154700
C	2.87535800	-0.58792500	0.55042900
C	2.25175600	-0.03454600	-0.75008600
C	3.09996100	-1.11318600	-1.54751300
C	3.59222500	-0.76100900	-2.95426100
H	4.31922400	-1.50690800	-3.29935700
H	2.75500900	-0.76826300	-3.66413100
H	4.06380600	0.22246500	-3.01721900
C	2.41076400	-2.48504600	-1.60304200
H	3.11658000	-3.23023300	-1.99022900
H	2.05996400	-2.83533800	-0.62901100
H	1.54764300	-2.46102100	-2.27823900
C	2.82199400	1.34382400	-1.03962100
C	4.26941500	1.51325500	-0.58216200
H	4.78580800	2.23472900	-1.22409400
H	4.24188800	1.96695600	0.41730400
C	2.13863100	2.32562900	-1.63493000
H	2.60948300	3.28067300	-1.85607000
H	1.09104600	2.23375500	-1.90369300
H	0.53699800	0.14715600	-1.73133400
H	3.04754200	0.11761600	1.36816700
H	2.31450200	-1.44155200	0.92983100
H	4.61181400	-1.92967900	-0.05880000
H	5.67960500	0.02784900	-1.37763200
H	5.73703000	0.21441700	0.36779800

Alpha site – Quartet state

Coordinate I

Fe	1.46165200	0.01802400	0.19463400
N	1.85111000	2.01056800	-0.23522400
C	2.67895600	2.46206700	-1.21949500
C	2.59124900	3.92593900	-1.28640800
C	1.70214600	4.31031900	-0.34060100
C	1.23086900	3.08849200	0.32255700
C	0.29686600	3.04296400	1.36629700
C	-0.17096500	1.89419700	2.01848400
C	-1.14367900	1.87914900	3.11852900
C	-1.31905500	0.58299300	3.46941400
C	-0.45733400	-0.21686100	2.58932000
N	0.21079200	0.61612400	1.74280100
C	-0.34423600	-1.61298100	2.62167000
C	0.46105800	-2.41011100	1.79727300
C	0.54414300	-3.87435600	1.86056700
C	1.41892100	-4.26028700	0.90213900
C	1.88668100	-3.03866000	0.23595000
N	1.28660400	-1.95883900	0.81084200
C	2.80864200	-2.99442800	-0.81844200
C	3.27624600	-1.84542700	-1.46971700
C	4.25449700	-1.82994000	-2.56482300
C	4.44366400	-0.53305800	-2.90327600
C	3.58388800	0.26685300	-2.02127500
C	3.48360800	1.66427500	-2.04364300
H	4.09025100	2.18320800	-2.77857000
N	2.90730600	-0.56622300	-1.18212200
H	5.09247600	-0.12459900	-3.66699100
H	4.71541900	-2.71117300	-2.99149100
H	3.20183700	-3.94509900	-1.16322300
H	1.73768000	-5.26230500	0.64641700

H	-0.00673400	-4.49291900	2.55689300
H	-0.94586800	-2.13212400	3.36055300
H	-1.95706600	0.17592400	4.24287600
H	-1.60707500	2.75992400	3.54340100
H	-0.10393900	3.99301100	1.70388800
H	1.37584500	5.31157200	-0.09148400
H	3.14794900	4.54518100	-1.97747600
O	0.22993000	-0.08721600	-0.90079300
S	3.33506500	0.14847000	1.84911000
H	3.13268400	1.41633200	2.27087000
C	-6.34563800	-0.71580200	-0.91454500
C	-5.58015200	0.31576700	-0.06741200
C	-4.44838700	-0.40639400	0.71676700
C	-3.58828000	-0.25394300	-0.57651400
C	-4.46066100	1.02339000	-0.91037800
C	-4.72973800	1.36107600	-2.37925100
H	-5.48926700	2.14985100	-2.45948000
H	-3.81497300	1.73648400	-2.85626400
H	-5.07199900	0.50405900	-2.96485200
C	-3.91927100	2.28369500	-0.21687400
H	-4.65077700	3.09941300	-0.28570900
H	-3.69413800	2.13080900	0.84280500
H	-2.99687300	2.62374800	-0.70507700
C	-3.93170500	-1.36450300	-1.53826000
C	-5.38965100	-1.81195000	-1.47137900
H	-5.72766100	-2.17127000	-2.44964000
H	-5.43803900	-2.68154000	-0.80229300
C	-3.03519500	-1.91389400	-2.36563900
H	-3.30906800	-2.69484300	-3.07265600
H	-1.99284600	-1.60216500	-2.35709900
H	-2.50636200	-0.11023800	-0.48405500

H	-4.64754300	-1.42197400	1.07549800
H	-4.05599500	0.18320300	1.54758000
H	-6.26725800	0.96574300	0.48850900
H	-6.86902300	-0.20633200	-1.73226900
H	-7.12068500	-1.19120000	-0.30085400

Coordinate II – TS (-1756.48)

Fe	-1.12266300	0.02568200	0.30660800
N	-2.19933700	-1.36748900	-0.67905200
C	-3.14005900	-1.16871000	-1.65483100
C	-3.64632500	-2.43281200	-2.13812800
C	-2.99194800	-3.40360400	-1.44335100
C	-2.09446700	-2.72741800	-0.53428900
C	-1.26015700	-3.37256800	0.37415500
C	-0.42272600	-2.75894400	1.30549100
C	0.36831300	-3.46988400	2.28243000
C	1.00476200	-2.52485000	3.03023900
C	0.60778700	-1.24348400	2.49730000
N	-0.26149800	-1.40602200	1.44939100
C	1.05917300	-0.01298900	2.97021500
C	0.70996900	1.23163000	2.45251000
C	1.20180300	2.49574200	2.94689000
C	0.62423400	3.46247100	2.18050300
C	-0.22256600	2.78264700	1.22801200
N	-0.15413400	1.42672800	1.40488400
C	-1.02379200	3.42858700	0.28613400
C	-1.90369300	2.81918600	-0.60361100
C	-2.75516900	3.53227500	-1.52832300
C	-3.47461700	2.59145200	-2.19980700
C	-3.05469100	1.30807300	-1.68584600
C	-3.54325900	0.07916500	-2.12460500

H	-4.29796300	0.09535300	-2.90489600
N	-2.10150900	1.46620700	-0.71532600
H	-4.21950700	2.73359000	-2.97188400
H	-2.78694800	4.60905000	-1.63246000
H	-0.97324500	4.51294300	0.26098100
H	0.74210400	4.53630400	2.24511600
H	1.89448800	2.60955100	3.77054300
H	1.74980300	-0.02645000	3.80774400
H	1.68154100	-2.66500300	3.86299100
H	0.41118800	-4.54716700	2.37604900
H	-1.28281200	-4.45816100	0.37651200
H	-3.09698700	-4.47794000	-1.52104100
H	-4.40012600	-2.54265700	-2.90686300
O	0.11834200	-0.04650700	-0.95358500
S	-2.94899400	0.10887200	1.96590500
H	-2.20179200	0.15793700	3.09061800
C	5.39913900	0.16058800	-1.01501200
C	4.46242200	-1.01881600	-0.69846100
C	3.45912200	-0.59408600	0.41606700
C	2.64222000	-0.00036800	-0.75105800
C	3.24615300	-1.09357400	-1.70449700
C	3.46555000	-0.74327300	-3.17616800
H	4.08211100	-1.51083600	-3.66113700
H	2.50424600	-0.71133000	-3.70437300
H	3.95006900	0.22453200	-3.32376000
C	2.53076900	-2.45166100	-1.62512000
H	3.14318200	-3.22349600	-2.10830300
H	2.33441100	-2.77723500	-0.60001700
H	1.56971000	-2.40714200	-2.14929000
C	3.14036000	1.36221000	-1.13152000
C	4.64545700	1.51503200	-0.89146300

H	5.06303800	2.26926500	-1.56702600
H	4.78982700	1.90276400	0.12542900
C	2.38495800	2.32935400	-1.66454100
H	2.81640100	3.28035200	-1.96999600
H	1.31594300	2.20033900	-1.80118700
H	3.81071900	0.09147900	1.19177500
H	2.97678500	-1.44862400	0.89074200
H	5.01175200	-1.95790200	-0.55074800
H	5.82161200	0.04048700	-2.01917800
H	6.24544100	0.15032400	-0.31783700
H	1.27443600	-0.02214700	-0.70128000

Coordinate III

Fe	-1.28387100	0.02142100	0.29029600
N	-1.18842500	-1.96223800	0.62654600
C	-1.81605600	-2.94739700	-0.09825700
C	-1.44325300	-4.24331000	0.40948400
C	-0.57506100	-4.03156300	1.43834700
C	-0.41116400	-2.60522100	1.56226600
C	0.43509700	-1.99366000	2.47617800
C	0.63671400	-0.62449900	2.57543700
C	1.48683000	0.00329400	3.55532300
C	1.38674700	1.34891500	3.36324800
C	0.47199700	1.54877300	2.26781100
N	0.04366200	0.33373500	1.78730100
C	0.05841100	2.79151200	1.81005300
C	-0.87500100	2.99409100	0.80429000
C	-1.27895900	4.29157300	0.32455700
C	-2.17525400	4.08188700	-0.67989800
C	-2.32134000	2.65504600	-0.82055100
N	-1.53638200	2.00952200	0.10547600

C	-3.11576700	2.04268600	-1.77927100
C	-3.22705300	0.67149100	-1.95260200
C	-4.06835700	0.04337600	-2.94045600
C	-3.93805100	-1.30250200	-2.77373900
C	-3.01857300	-1.50267300	-1.68168500
C	-2.66677700	-2.74472200	-1.17590200
H	-3.09155000	-3.62151200	-1.65301400
N	-2.58385900	-0.28737000	-1.20634400
H	-4.41778300	-2.10141200	-3.32431600
H	-4.67663700	0.58021900	-3.65690300
H	-3.68535800	2.68282100	-2.44451800
H	-2.69150600	4.81268700	-1.28893400
H	-0.90640400	5.23061800	0.71302700
H	0.48935000	3.66939400	2.27952100
H	1.87062600	2.14753600	3.91053700
H	2.07093500	-0.53264200	4.29222900
H	0.97907700	-2.63328300	3.16302400
H	-0.07380400	-4.76146700	2.06086100
H	-1.80364700	-5.18325700	0.01195300
O	0.05344200	-0.01216600	-0.90066800
H	0.93818700	0.02309900	-0.46747100
S	-3.03231900	-0.02665900	1.88483100
H	-3.29882500	1.29672700	1.92830000
C	5.78378000	0.18119500	-1.01242900
C	4.85907100	-0.97658800	-0.58609000
C	3.93237500	-0.49024100	0.57414100
C	3.09260200	0.05556400	-0.58331800
C	3.57666400	-1.08657900	-1.51710300
C	3.70708100	-0.80694000	-3.01325700
H	4.27262800	-1.61039000	-3.50145500
H	2.71377900	-0.77344200	-3.47799900

H	4.20213800	0.14126700	-3.23459800
C	2.84534500	-2.42521900	-1.32284900
H	3.41010100	-3.23379100	-1.80305200
H	2.70593900	-2.69319700	-0.27208300
H	1.85451400	-2.38232400	-1.78977400
C	3.53253600	1.39810100	-1.03990100
C	5.05483800	1.55305800	-0.90436200
H	5.43162200	2.26717000	-1.64423400
H	5.26876800	1.98875600	0.07995900
C	2.73076100	2.34066100	-1.54963300
H	3.12595700	3.28740600	-1.91136400
H	1.65579000	2.19567000	-1.61144700
H	4.33700500	0.22633900	1.29298500
H	3.46512300	-1.31565600	1.11298500
H	5.40523600	-1.91698500	-0.43102100
H	6.14188400	0.01137900	-2.03399400
H	6.67098800	0.18900800	-0.36871100

Coordinate V

Fe	1.35681500	-0.02094000	0.21469500
N	1.52175100	0.37172700	-1.81878900
C	1.72256200	-0.55209700	-2.81604300
C	1.95320400	0.12249500	-4.07004300
C	1.91988400	1.46232600	-3.81069800
C	1.66830200	1.60892100	-2.39779800
C	1.63923700	2.81409300	-1.70385900
C	1.43372300	2.95541600	-0.33516600
C	1.34093700	4.22771700	0.33935500
C	1.04425800	3.96977600	1.64314200
C	0.95314100	2.53630600	1.78251900
N	1.21517300	1.93723200	0.56880300

C	0.58265700	1.88948800	2.95735600
C	0.42722700	0.51546300	3.10795200
C	0.06337600	-0.15650400	4.33240400
C	0.09673200	-1.49579600	4.07283200
C	0.48112500	-1.64403200	2.68952500
N	0.65267800	-0.40796300	2.11806000
C	0.70273000	-2.85378000	2.04027500
C	1.09123100	-2.99901500	0.71234400
C	1.25704700	-4.27211400	0.05365400
C	1.55222700	-4.01322400	-1.25025700
C	1.57028100	-2.57860200	-1.40474500
C	1.75679000	-1.92913700	-2.62121100
H	1.93838400	-2.55318100	-3.49025500
N	1.30499200	-1.98008600	-0.19102400
H	1.72478900	-4.71613100	-2.05497300
H	1.13748300	-5.23143500	0.54031600
H	0.55243100	-3.76381600	2.61198800
H	-0.10731400	-2.31692900	4.74802700
H	-0.17390500	0.34223300	5.26338400
H	0.39109900	2.51363200	3.82415200
H	0.87726700	4.67333400	2.44847700
H	1.46751300	5.18663500	-0.14636400
H	1.78098800	3.72487700	-2.27656600
H	2.06280200	2.28387000	-4.50089400
H	2.12855500	-0.37491200	-5.01530300
O	-1.18524100	0.14962000	-0.59793700
S	3.70808900	-0.13210000	0.69809600
H	3.91162800	1.16854100	1.00444800
C	-5.30484000	-0.42788300	0.23707000
C	-4.35919300	0.75597600	0.48947500
C	-3.02320700	0.24378900	1.10405100

C	-2.57208100	-0.02077900	-0.35244900
C	-3.55744600	1.13703300	-0.80510000
C	-4.23526900	1.02678000	-2.17406500
H	-5.02138900	1.78608800	-2.27281400
H	-3.50662700	1.20572700	-2.97536900
H	-4.68758700	0.04921900	-2.35818500
C	-2.91283400	2.52710700	-0.69166900
H	-3.68155600	3.29862100	-0.82375200
H	-2.42783000	2.70360100	0.27204000
H	-2.15800700	2.67159900	-1.47345200
C	-3.13536400	-1.35567700	-0.81048100
C	-4.50870200	-1.68022200	-0.22470400
H	-5.08952100	-2.28055800	-0.93312900
H	-4.34360300	-2.32545100	0.64814000
C	-2.48891100	-2.18742400	-1.63289500
H	-2.94188100	-3.12057600	-1.95993400
H	-1.48541300	-1.98588500	-1.99619700
H	-1.01553800	0.12148800	-1.55437800
H	-3.06209700	-0.61163700	1.78549900
H	-2.43410500	1.03852300	1.56369500
H	-4.86575900	1.58084400	1.00235000
H	-6.05686500	-0.14668400	-0.50915400
H	-5.85372900	-0.66534200	1.15611300

Delta Site – Doublet state

Coordinate I (cis)

Fe	-1.46281500	0.00757500	0.13266300
N	-0.83911300	-1.16040200	1.65742000
C	-0.75057300	-2.52864000	1.67289400

C	-0.17121800	-2.98520900	2.91307400
C	0.09182800	-1.87184000	3.65224400
C	-0.32600500	-0.74190000	2.85805400
C	-0.22005600	0.58487800	3.26338200
C	-0.61518500	1.69462500	2.52411500
C	-0.48586800	3.06014100	2.97153700
C	-0.96150000	3.84609500	1.96673000
C	-1.38267300	2.95681200	0.91154900
N	-1.17080700	1.64944400	1.27020300
C	-1.93960100	3.37222700	-0.29392800
C	-2.38742900	2.53362900	-1.30967000
C	-2.99709500	2.98772000	-2.53612900
C	-3.30413300	1.87258700	-3.25427200
C	-2.87754900	0.74262700	-2.46476400
N	-2.32757100	1.16321900	-1.28008900
C	-3.00384800	-0.58473600	-2.86019000
C	-2.59914300	-1.69441100	-2.12474900
C	-2.74697100	-3.06057700	-2.56435100
C	-2.22724600	-3.84454800	-1.57975500
C	-1.76667600	-2.95285700	-0.54331400
C	-1.17586000	-3.36662100	0.64659200
H	-1.04634600	-4.43452500	0.79137300
N	-2.00425600	-1.64751300	-0.89001100
H	-2.15720600	-4.92355300	-1.53923200
H	-3.19277800	-3.35966800	-3.50379300
H	-3.45819900	-0.77154000	-3.82808400
H	-3.77427500	1.80124900	-4.22626600
H	-3.16275100	4.02562400	-2.79333000
H	-2.04744500	4.44119100	-0.44852100
H	-1.03207100	4.92507300	1.92665000
H	-0.08214600	3.35740200	3.93038600

H	0.20927400	0.77024400	4.24289000
H	0.52367200	-1.80246200	4.64196000
H	-0.00256000	-4.02304000	3.16858600
S	-3.77575300	-0.11545600	1.20173300
H	-4.07627100	1.20086900	1.14935000
O	0.01799800	0.08992100	-0.55949500
C	3.75567400	-0.67043700	-2.16271200
C	5.10037900	0.06516400	-2.03748100
C	5.10187100	1.04035300	-0.80719000
C	5.45523200	-0.24671600	0.04188900
C	6.10001900	-0.83061200	-1.25358300
H	6.03742700	-1.91356100	-1.40492000
H	7.14028400	-0.52273300	-1.37584800
C	4.19278600	-1.00961900	0.35716500
C	3.43044300	-1.48799600	-0.87697600
H	3.71030800	-2.53531800	-1.05318300
C	3.78622300	-1.26073500	1.60679700
H	2.86159200	-1.79561900	1.81565000
H	4.37238500	-0.94648400	2.46798500
H	6.08324400	-0.10310300	0.92861300
C	6.27329700	2.03422900	-0.85432600
H	6.38758500	2.53935900	0.11326700
H	6.08504500	2.80658500	-1.61128600
H	7.23020600	1.56218600	-1.09643900
C	3.82639600	1.81503200	-0.46388100
H	3.61976100	2.57167000	-1.23230300
H	3.94864200	2.34275900	0.49109100
H	2.94225300	1.17892700	-0.37385000
H	5.41983400	0.48261300	-3.00036500
H	3.79033900	-1.34972600	-3.02338300
H	2.95762300	0.05177500	-2.37152300

H	2.35292400	-1.49045400	-0.68165000

Coordinate II (cis) – TS (-1697.84)

Fe	-1.22461100	-0.00001900	0.22356500
N	-2.16691100	0.37210900	-1.53619000
C	-2.32467400	1.59953500	-2.12213700
C	-2.97629000	1.46815900	-3.40562000
C	-3.21008200	0.13939000	-3.58838800
C	-2.69411000	-0.53457700	-2.41904800
C	-2.71541000	-1.91296200	-2.22409800
C	-2.18903400	-2.58346800	-1.12307200
C	-2.18367600	-4.01894200	-0.96014700
C	-1.55768600	-4.27360500	0.22199000
C	-1.19016100	-2.99170100	0.77845800
N	-1.58749400	-1.97703000	-0.05078800
C	-0.53652400	-2.81803500	1.99635500
C	-0.19960400	-1.60112300	2.58365800
C	0.40043400	-1.46985100	3.89071200
C	0.53171500	-0.13531900	4.12813600
C	0.02097500	0.54262900	2.96074400
N	-0.41656000	-0.36485600	2.02830900
C	-0.01186700	1.92450800	2.79697200
C	-0.48048300	2.58903900	1.66620600
C	-0.48024800	4.02181300	1.49478600
C	-1.00275600	4.26550000	0.26056000
C	-1.33000800	2.98066600	-0.31208700
C	-1.92730700	2.80955700	-1.55819400
H	-2.12191200	3.70838400	-2.13541700
N	-0.99803300	1.97072600	0.55641700
H	-1.16691200	5.21706200	-0.22808500
H	-0.12282600	4.73104600	2.22982800

H	0.36693900	2.53293600	3.61226400
H	0.93345000	0.36004200	5.00241700
H	0.66974000	-2.30010200	4.53048300
H	-0.28896900	-3.71642500	2.55359900
H	-1.35748400	-5.22887100	0.68951300
H	-2.60328300	-4.72150100	-1.66832500
H	-3.16545600	-2.51796400	-3.00517200
H	-3.68087100	-0.35535500	-4.42792800
H	-3.21641800	2.29391300	-4.06260100
S	-3.41852800	0.14982300	1.31974200
H	-4.15079100	-0.51676000	0.40060400
O	0.25317500	-0.17851900	-0.68134300
C	3.17492300	-0.62188000	0.81470900
C	4.51988900	-0.89423600	0.11824700
C	4.39155900	-0.84229800	-1.44620000
C	4.50232300	0.72035400	-1.26830100
C	5.31254400	0.43754200	0.03808800
H	5.18488400	1.14738400	0.86291300
H	6.37938400	0.30046700	-0.14764600
C	3.19250300	1.34373700	-0.84144300
C	2.47779400	0.63367700	0.25662000
H	2.10334000	1.31261400	1.02753200
C	2.73760100	2.49610200	-1.37187100
H	1.81674700	2.96097200	-1.02702500
H	3.27987800	3.00810200	-2.16277700
H	4.97969600	1.29024400	-2.07298600
C	5.63577400	-1.41834600	-2.14397800
H	5.62378400	-1.16907000	-3.21258000
H	5.64254000	-2.51250100	-2.05898000
H	6.57828100	-1.04969900	-1.72887800
C	3.15337200	-1.43227400	-2.12711100

H	3.16893400	-2.52810600	-2.05514600
H	3.15482100	-1.17802600	-3.19546100
H	2.20815800	-1.08481100	-1.70556400
H	5.01559500	-1.77126900	0.55177900
H	3.34814900	-0.49407500	1.89155400
H	2.51581100	-1.49438100	0.71631000
H	1.34980800	0.25905200	-0.20353900

Coordinate III (cis)

Fe	-1.44376200	0.04864600	0.25151800
N	-0.03826300	0.19745500	1.69678800
C	0.51316900	-0.80563100	2.45369300
C	1.47889200	-0.26827700	3.38223500
C	1.50635100	1.07880700	3.17694900
C	0.55083400	1.35719000	2.12995600
C	0.25066700	2.63555400	1.66008200
C	-0.70866700	2.96134700	0.70343800
C	-1.00578500	4.30823100	0.27075300
C	-1.99491900	4.20820700	-0.65978400
C	-2.29227400	2.80046500	-0.79372300
N	-1.50516000	2.06004400	0.04758000
C	-3.23604200	2.26785800	-1.67010900
C	-3.51455300	0.91272700	-1.83465700
C	-4.47614700	0.37694600	-2.77102400
C	-4.42969000	-0.97764900	-2.64090100
C	-3.44385700	-1.26069700	-1.62176400
N	-2.90251100	-0.09803500	-1.14188800
C	-3.10673800	-2.54104800	-1.18636700
C	-2.19055500	-2.86064400	-0.18568800
C	-1.92866900	-4.20303400	0.27893200
C	-1.01634100	-4.09701100	1.28496800

C	-0.71786200	-2.69135100	1.42264100
C	0.18768900	-2.15568900	2.33667700
H	0.69278300	-2.84917700	3.00194600
N	-1.43960300	-1.95442000	0.51809200
H	-0.57543300	-4.88345400	1.88347800
H	-2.39516400	-5.09499200	-0.11869100
H	-3.62414400	-3.37102800	-1.65813300
H	-5.00041700	-1.72780500	-3.17268600
H	-5.09138400	0.97219600	-3.43316100
H	-3.79277300	2.96782700	-2.28563400
H	-2.48412200	4.99831700	-1.21443400
H	-0.51371300	5.19792200	0.64168800
H	0.80422100	3.46024800	2.09880800
H	2.10155600	1.82714500	3.68386000
H	2.04904500	-0.85598300	4.08981000
S	-3.15391400	0.20780100	1.96136900
H	-3.98314400	1.07817000	1.34390300
O	-0.19374600	-0.03921100	-1.07240500
H	0.52653500	-0.61510000	-0.75807300
C	5.23545100	-0.29682400	-2.70762400
C	5.72214800	0.23441700	-1.34654700
C	4.52777100	0.81539300	-0.50334100
C	4.24476200	-0.69202600	-0.12452200
C	5.76618900	-0.95394100	-0.34583300
H	6.04433800	-1.93517900	-0.74532500
H	6.36787200	-0.74813900	0.54113700
C	3.49848400	-1.39184400	-1.24879100
C	4.03740900	-1.18669600	-2.52960100
H	3.58429100	-1.65717600	-3.39950000
C	2.38947600	-2.18194400	-1.01865100
H	1.89357500	-2.70497800	-1.83216000

H	2.02019300	-2.35991300	-0.01310800
H	3.80527900	-0.89799200	0.85691700
C	5.01472200	1.62589400	0.70891600
H	4.18313300	1.81264500	1.39992200
H	5.40143000	2.59967100	0.38260700
H	5.80848900	1.12838700	1.27355000
C	3.43857400	1.62579200	-1.21146500
H	3.84563000	2.58253000	-1.56343100
H	2.61964400	1.85220000	-0.51676800
H	3.00519200	1.10880400	-2.07160000
H	6.61451600	0.86096400	-1.45643400
H	6.04594100	-0.85471700	-3.20391000
H	4.99287600	0.53023400	-3.39379000

Coordinate V (cis)

Fe	1.31044600	0.06450600	0.27348000
N	1.31633200	2.02810600	-0.19860600
C	1.78428200	2.62529000	-1.34765900
C	1.67701200	4.06002600	-1.24418800
C	1.15009300	4.32719600	-0.01654400
C	0.93361600	3.05580200	0.63197000
C	0.41597700	2.91177200	1.91322300
C	0.20016100	1.70403800	2.56608100
C	-0.35315600	1.59103900	3.89497300
C	-0.42236500	0.26090400	4.18246500
C	0.09033200	-0.44028900	3.03070800
N	0.45983300	0.45507600	2.05319700
C	0.19628600	-1.82216300	2.93220800
C	0.69930000	-2.49701900	1.82673800
C	0.82484300	-3.93101900	1.73004000
C	1.36954400	-4.19550300	0.50976800

C	1.57736100	-2.92329000	-0.14098000
N	1.15603800	-1.89978000	0.67420800
C	2.12361600	-2.77846000	-1.41070000
C	2.33689400	-1.57155500	-2.06565800
C	2.90240900	-1.45818100	-3.39003500
C	2.95099000	-0.12949800	-3.68550300
C	2.41509600	0.56990400	-2.54223500
C	2.29665200	1.95102300	-2.44876000
H	2.63106300	2.54486000	-3.29336000
N	2.04288600	-0.32523800	-1.56723800
H	3.30933300	0.34994200	-4.58759600
H	3.21245300	-2.29753800	-3.99956200
H	2.40664900	-3.68540500	-1.93583100
H	1.61351200	-5.15575400	0.07328100
H	0.52905200	-4.62814100	2.50365000
H	-0.13159200	-2.41532600	3.77984700
H	-0.78428400	-0.21777200	5.08351100
H	-0.64776700	2.43110100	4.51112000
H	0.15659400	3.81926100	2.44952100
H	0.92207000	5.28894100	0.42523900
H	1.97127300	4.75626400	-2.01919500
S	3.41313000	0.18200300	1.13728400
H	3.20531800	-0.53187100	2.26583200
C	-2.71913000	0.74673500	0.43518600
C	-4.23467300	0.48160700	0.37900200
C	-4.73752100	0.34190100	-1.10165900
C	-4.21980600	-1.15066100	-0.99096800
C	-4.49014600	-1.04453300	0.54231400
H	-3.83708600	-1.60570700	1.21857400
H	-5.52537900	-1.27744800	0.79523700
C	-2.73144900	-1.18715300	-1.24359300

C	-1.92417600	-0.43260600	-0.18567700
H	-1.67532500	-1.13777900	0.61100600
C	-2.16442800	-1.81180200	-2.27979600
H	-1.09123400	-1.78837800	-2.44337600
H	-2.76489400	-2.36307900	-2.99938300
H	-4.75109400	-1.91167800	-1.57239000
C	-6.27021800	0.41348100	-1.20203300
H	-6.60462000	0.07440600	-2.19034100
H	-6.60771300	1.44994200	-1.07498100
H	-6.78385500	-0.19412800	-0.45161700
C	-4.14889500	1.24635200	-2.18711800
H	-4.43340400	2.29155500	-2.01096900
H	-4.53893700	0.95957700	-3.17188900
H	-3.05888600	1.20168100	-2.25177100
H	-4.78181200	1.17527800	1.02769800
H	-2.39435400	0.88419500	1.47128300
H	-2.47629100	1.67665200	-0.09431200
O	-0.62884500	-0.03284700	-0.67390500
H	-0.71634000	0.69274300	-1.31536500

Coordinate I (trans)

Fe	1.45284200	0.05421500	0.17468700
N	2.64324500	0.13839700	-1.45499200
C	3.18701700	-0.92294700	-2.13402400
C	3.89734000	-0.46490300	-3.30359000
C	3.77022800	0.89010500	-3.33114800
C	2.98401100	1.25427900	-2.17735200
C	2.63470700	2.55780500	-1.84100300
C	1.90802800	2.93678700	-0.71657000
C	1.58859500	4.30081700	-0.37157600
C	0.88222500	4.25257300	0.79149500

C	0.77078500	2.85940700	1.14949500
N	1.40766900	2.07188400	0.22362700
C	0.10005700	2.38822400	2.27321500
C	-0.03165600	1.05269100	2.64134900
C	-0.73374100	0.59671100	3.81649500
C	-0.64013500	-0.76179100	3.82304000
C	0.12099800	-1.12752900	2.65272000
N	0.48418500	-0.01036700	1.94561100
C	0.45363000	-2.43379100	2.30546400
C	1.20953000	-2.80928400	1.19953400
C	1.56412000	-4.17007600	0.87700400
C	2.30458500	-4.11876300	-0.26450300
C	2.39761800	-2.72712100	-0.63367900
C	3.07100200	-2.25609300	-1.75646400
H	3.54996400	-2.99473000	-2.39147900
N	1.72952900	-1.94300300	0.27194100
H	2.75514800	-4.93284500	-0.81697500
H	1.27841800	-5.03504400	1.46092600
H	0.10310000	-3.22658800	2.95864600
H	-1.03649100	-1.46317300	4.54522000
H	-1.22464600	1.24476600	4.53051900
H	-0.36098300	3.12803900	2.91987700
H	0.46740000	5.07010000	1.36642000
H	1.87826200	5.16653800	-0.95264100
H	2.96832200	3.34827900	-2.50556600
H	4.16700900	1.59168700	-4.05293500
H	4.41875200	-1.10976700	-3.99858700
S	3.61269400	0.21488900	1.52618900
H	4.14418600	1.28607000	0.89656600
O	0.07910100	-0.06101400	-0.70896700
C	-3.97807900	-0.76262500	0.84782700

C	-4.56547100	0.57274600	0.35815200
C	-3.61517800	1.18755900	-0.70893000
C	-4.34866100	0.27787300	-1.74335500
C	-5.61070700	0.34599600	-0.79161300
C	-3.75591100	-1.10904300	-1.69588400
C	-3.26442300	-1.52020200	-0.31073700
H	-3.34131800	-2.60584200	-0.18143000
C	-3.65886400	-1.89926200	-2.77178500
H	-3.25636300	-2.90860500	-2.70869400
H	-3.97557200	-1.56061100	-3.75603100
H	-4.47778700	0.63775400	-2.77061500
H	-4.88304200	1.20325100	1.19792700
H	-4.77271500	-1.37933800	1.28476900
H	-3.25644000	-0.57274200	1.65225300
H	-2.19315200	-1.28357000	-0.26586500
C	-6.54374200	-0.86584000	-0.72792800
H	-7.25810300	-0.75756800	0.09876000
H	-7.12513100	-0.94697600	-1.65557000
H	-6.01457500	-1.81251300	-0.59368700
C	-6.46273000	1.59733200	-1.05760800
H	-7.20427100	1.72759000	-0.25875300
H	-5.87342300	2.51748100	-1.11286400
H	-7.00960100	1.49599400	-2.00379200
H	-2.53834600	1.03581400	-0.58114800
H	-3.80187800	2.24864000	-0.88595900

Coordinate II (trans) – TS (-1661.71)

Fe	-1.27880700	0.00789600	0.20876200
N	-0.72563000	1.67336100	1.19205200
C	-0.15826500	1.77370300	2.43799100
C	0.09007200	3.15542100	2.77230000

C	-0.33186500	3.89426600	1.70837100
C	-0.84534800	2.96089300	0.73332800
C	-1.40861800	3.33356200	-0.48492100
C	-1.98724600	2.47919100	-1.42018900
C	-2.59874600	2.91090000	-2.65645200
C	-3.05520500	1.78819900	-3.27691500
C	-2.71384500	0.67593400	-2.42031400
N	-2.07211700	1.11816100	-1.29283400
C	-2.98502500	-0.65924400	-2.70873800
C	-2.62977300	-1.74726600	-1.91612300
C	-2.88350400	-3.12894000	-2.25363600
C	-2.35897100	-3.87793400	-1.24473400
C	-1.79362200	-2.94924800	-0.29253100
N	-1.97350100	-1.65958500	-0.71545200
C	-1.16567700	-3.31996600	0.89467400
C	-0.64263200	-2.45693800	1.85412800
C	-0.07529100	-2.88814500	3.11026400
C	0.27764400	-1.76043800	3.78711600
C	-0.06571000	-0.64584100	2.93722400
C	0.14524500	0.69211200	3.25974200
H	0.59118800	0.90847800	4.22549700
N	-0.62209000	-1.08728700	1.76345800
H	0.72852100	-1.67255300	4.76692600
H	0.02250700	-3.92059700	3.41957600
H	-1.09919600	-4.38339100	1.10366700
H	-2.34830100	-4.95471000	-1.13677400
H	-3.39216700	-3.46139600	-3.14913100
H	-3.50214200	-0.87101200	-3.63958200
H	-3.57119700	1.70135400	-4.22419100
H	-2.66280900	3.93957300	-2.98649900
H	-1.42012200	4.39467100	-0.71505800

H	-0.31411200	4.96891200	1.58207200
H	0.52910400	3.49576600	3.70101600
S	-3.45914100	0.17152100	1.33862800
H	-4.25646600	-0.09025400	0.27981100
O	0.17868600	-0.13614100	-0.72949700
C	3.06985900	-1.29417000	0.23158200
C	3.96283600	-1.46287000	-1.00974400
C	3.31093600	-0.70484400	-2.20054700
C	4.01607900	0.57056000	-1.64512300
C	5.11268100	-0.39374400	-1.03607000
C	3.17971900	1.14662900	-0.52633000
C	2.49286600	0.13058800	0.31019300
H	2.26426300	0.47083600	1.32362900
C	3.06342700	2.47239500	-0.30751700
H	2.47147700	2.86641800	0.51534100
H	3.56256000	3.19737200	-0.94579300
H	4.35427900	1.34075000	-2.34687300
H	4.25361500	-2.51052100	-1.15229600
H	3.63930800	-1.51749100	1.14423800
H	2.25059400	-2.02379800	0.19841300
H	1.32065200	0.04147600	-0.16778700
C	5.79470700	0.00508500	0.27546800
H	6.39040400	-0.83027400	0.66578100
H	6.47933800	0.84652200	0.10770800
H	5.09155600	0.30807300	1.05563900
C	6.20367600	-0.73928600	-2.06179600
H	6.82492600	-1.56424000	-1.69005900
H	5.80272400	-1.04079200	-3.03392600
H	6.86186500	0.12315500	-2.22660100
H	2.21789200	-0.68996800	-2.24791000
H	3.70642300	-1.00876800	-3.17148100

Coordinate III (trans)

Fe	-1.42204900	-0.01498900	0.23093400
N	-2.64649800	-0.63187200	-1.25349300
C	-3.29808300	0.17498000	-2.14794600
C	-4.06156900	-0.62133800	-3.08310200
C	-3.85914900	-1.92376200	-2.74083400
C	-2.97147100	-1.91609900	-1.59990300
C	-2.50752100	-3.05776600	-0.94914900
C	-1.64456900	-3.06969400	0.14320900
C	-1.15873300	-4.26428500	0.79603600
C	-0.33660400	-3.85048600	1.79967400
C	-0.32683400	-2.40476300	1.75635400
N	-1.13015500	-1.95159100	0.74599500
C	0.39601200	-1.59766400	2.63569400
C	0.42023400	-0.20387000	2.64571700
C	1.13961800	0.59257700	3.61314300
C	0.90443300	1.89824200	3.29836200
C	0.04523500	1.89380800	2.13755000
N	-0.23551700	0.60636100	1.75406100
C	-0.42836800	3.03401900	1.49247000
C	-1.26654600	3.04175100	0.37819600
C	-1.73929500	4.23417100	-0.28564700
C	-2.52575500	3.81805100	-1.31718700
C	-2.53276100	2.37362700	-1.27646000
C	-3.24257700	1.56722600	-2.16323400
H	-3.81382000	2.07287700	-2.93599600
N	-1.75928000	1.92173600	-0.23810000
H	-3.05940100	4.41678300	-2.04401200
H	-1.49217300	5.24531500	0.01089300
H	-0.12118500	3.99592000	1.89121000

H	1.26844800	2.78869500	3.79441900
H	1.73692500	0.19058400	4.42133100
H	0.97688000	-2.10354400	3.40106400
H	0.21713100	-4.45014000	2.51055400
H	-1.42072000	-5.27464600	0.50967000
H	-2.84203500	-4.01795400	-1.32989600
H	-4.26095100	-2.81536500	-3.20467800
H	-4.66477500	-0.21933500	-3.88694300
S	-3.30404200	0.05917100	1.74539600
H	-3.91505200	-1.08832600	1.37523500
O	0.00046400	-0.09842200	-0.92159400
C	3.65681500	-0.75767800	0.67179900
C	4.26153300	-1.36017800	-0.60980900
C	3.30969200	-1.02866500	-1.79326100
C	4.04163000	0.34770100	-1.86452800
C	5.30816600	-0.38017800	-1.25871300
C	3.41393900	1.25102300	-0.81809400
C	3.22028100	0.65146900	0.44250800
H	2.79431800	1.22111300	1.26428000
C	3.06469100	2.56123000	-1.05291900
H	2.62061000	3.17229000	-0.27209200
H	3.22131500	3.02554700	-2.02184000
H	4.14993400	0.84791200	-2.83194200
H	4.56924600	-2.40004200	-0.45567300
H	4.36263700	-0.79101400	1.51720400
H	2.79041400	-1.35457800	1.01047100
H	0.57737400	0.65192100	-0.70146200
C	6.26095800	0.40723200	-0.35451500
H	6.98785100	-0.26980300	0.11179700
H	6.82521900	1.14078500	-0.94434700
H	5.75338300	0.95247900	0.44597300

C	6.14418100	-1.06844300	-2.34946200
H	6.87903700	-1.74266500	-1.89196800
H	5.54449700	-1.65853700	-3.04804000
H	6.69686400	-0.32214000	-2.93355700
H	2.23657100	-1.00318800	-1.58003600
H	3.49033800	-1.64646400	-2.67441400

Coordinate V (trans)

Fe	-1.29276100	0.06239000	0.26233100
N	-0.54993800	1.06684400	1.83823100
C	-0.10644100	0.55537600	3.03601000
C	0.27011800	1.62542900	3.92791800
C	0.04082800	2.79084500	3.26119600
C	-0.47581000	2.43368000	1.96089300
C	-0.83457800	3.35485900	0.98415400
C	-1.33403600	3.04140300	-0.27411800
C	-1.68554600	4.02299700	-1.27363800
C	-2.12376700	3.33528300	-2.36444900
C	-2.04058600	1.93296500	-2.03278600
N	-1.55533600	1.77367000	-0.75603000
C	-2.40819300	0.90119700	-2.88754500
C	-2.33741500	-0.44959700	-2.57244500
C	-2.74906600	-1.51833900	-3.44785900
C	-2.54658100	-2.68345500	-2.77025100
C	-2.00981600	-2.32969500	-1.47889000
N	-1.87883400	-0.96191600	-1.37807900
C	-1.67241200	-3.24795700	-0.49229100
C	-1.15919100	-2.93333600	0.76084700
C	-0.81890400	-3.91422200	1.76438700
C	-0.35797600	-3.22787300	2.84721700
C	-0.41550300	-1.82742700	2.50676400
C	-0.03901700	-0.79460000	3.35624700

H	0.32868900	-1.06128600	4.34192500
N	-0.89919800	-1.66610300	1.22811900
H	-0.00684000	-3.61686500	3.79449300
H	-0.92472500	-4.98423900	1.63819700
H	-1.82332700	-4.29881200	-0.71831700
H	-2.73639000	-3.69709300	-3.09978900
H	-3.13973800	-1.37845900	-4.44770600
H	-2.78540900	1.17036200	-3.86895100
H	-2.47132000	3.72319500	-3.31351300
H	-1.59814200	5.09384000	-1.14050900
H	-0.71375200	4.40669400	1.22361300
H	0.20160100	3.80616300	3.60089200
H	0.65663800	1.48432000	4.92921000
S	-3.39979200	0.14708800	1.12170700
H	-4.05941600	0.44851800	-0.01870200
C	2.66287400	0.90798700	0.36872100
C	3.80295800	1.26814300	-0.59826500
C	4.85431700	0.10685000	-0.69976200
C	3.84647500	-0.52830500	-1.74165700
C	3.36740400	0.92410400	-2.04944900
H	2.31287300	1.06148500	-2.30412000
H	3.98180400	1.40985900	-2.80949700
C	2.75784500	-1.26172300	-1.00397700
C	1.94789800	-0.40624600	-0.02943400
C	2.47976600	-2.56118000	-1.16647100
H	1.70713000	-3.06024900	-0.58525200
H	3.01821900	-3.17039300	-1.88851300
H	4.27193600	-1.13361700	-2.54929900
C	6.15987700	0.56048400	-1.37119000
H	6.77420200	-0.30769000	-1.64082900
H	6.74487600	1.17909600	-0.67871600

H	5.99711100	1.14706000	-2.28005000
C	5.21171400	-0.69473700	0.55494800
H	5.76671500	-0.06763000	1.26437200
H	5.85560500	-1.54374700	0.29228400
H	4.34121300	-1.09960000	1.07866300
H	4.19006400	2.27293600	-0.39241000
H	1.91451900	1.70629800	0.38139800
H	3.04540200	0.81984000	1.39174100
O	0.68101400	-0.04487100	-0.68062100
H	1.68706000	-0.99064800	0.85584100
H	0.48346000	-0.72082400	-1.35405200

Delta Site – Quartet state

Coordinate I (cis)

Fe	-1.46470400	0.00608200	0.13290700
N	-1.25763400	1.70588900	1.20316500
C	-0.72061700	1.83791300	2.45812600
C	-0.67310900	3.22692800	2.84552700
C	-1.18681200	3.93858400	1.80450700
C	-1.54546100	2.98034100	0.78715300
C	-2.10649800	3.31273500	-0.44221000
C	-2.47400900	2.41388300	-1.43787600
C	-3.05965300	2.79126500	-2.70157700
C	-3.26446200	1.63828200	-3.39591000
C	-2.80453800	0.56155900	-2.55254600
N	-2.33102700	1.05118300	-1.36150700
C	-2.84941600	-0.78529700	-2.89836100
C	-2.43902400	-1.84198300	-2.09152300
C	-2.52114000	-3.23376200	-2.46278200
C	-2.02706700	-3.94644500	-1.41320600

C	-1.64378300	-2.98690500	-0.40618100
N	-1.90869400	-1.70960800	-0.83282300
C	-1.07750100	-3.31867600	0.82015100
C	-0.69960900	-2.41868700	1.81188500
C	-0.12585800	-2.79775400	3.08054400
C	0.09757900	-1.64273800	3.76692600
C	-0.34026800	-0.56495500	2.91290400
C	-0.28778300	0.78282300	3.25535700
H	0.11951600	1.03218300	4.23009000
N	-0.82448500	-1.05579700	1.72790700
H	0.51281600	-1.51355300	4.75768300
H	0.06799200	-3.81677600	3.38855900
H	-0.91950900	-4.37330400	1.02274600
H	-1.92400700	-5.01872900	-1.31145700
H	-2.91024400	-3.59719500	-3.40475700
H	-3.24936400	-1.03394100	-3.87632800
H	-3.68761100	1.50867100	-4.38326500
H	-3.27820600	3.80852700	-2.99871100
H	-2.27589200	4.36650900	-0.63991300
H	-1.32034500	5.00867700	1.71600200
H	-0.29620700	3.58895900	3.79299900
O	0.03121100	0.11091000	-0.52498100
S	-3.77511900	-0.14948600	1.15539800
H	-4.44635700	-0.53732800	0.04870000
C	3.76688500	-0.86435100	-2.01836600
C	5.09199800	-0.08375700	-2.00304900
C	5.06792200	1.05538400	-0.92309500
C	5.45415000	-0.08914800	0.09828700
C	6.11443900	-0.83354200	-1.10378900
H	6.08019000	-1.92824600	-1.10028600
H	7.14633300	-0.51923100	-1.27184500

C	4.21244200	-0.83288000	0.52249700
C	3.46367600	-1.50091500	-0.62910300
H	3.77224900	-2.55460900	-0.65684800
H	2.38659500	-1.50545200	-0.43156600
C	3.81345600	-0.91702400	1.79672200
H	2.90423900	-1.44262100	2.08264700
H	4.39122800	-0.46910700	2.60267100
H	6.07787500	0.19455100	0.95373600
C	6.21324700	2.06240700	-1.11401000
H	6.31334000	2.70243800	-0.22813000
H	6.00560800	2.71452400	-1.97226900
H	7.18246600	1.58599400	-1.28939100
C	3.77274300	1.83808800	-0.68912600
H	3.54748600	2.47264600	-1.55649800
H	3.88105800	2.49910000	0.18081600
H	2.90480900	1.19926100	-0.50723900
H	5.40068700	0.20127200	-3.01643200
H	3.81918700	-1.65690700	-2.77501500
H	2.94989800	-0.19984000	-2.32323800

Coordinate II (cis) – TS (-1858.04)

Fe	-1.22974600	-0.00575100	0.20468000
N	-2.17660100	0.44487700	-1.51507300
C	-2.32926000	1.69751800	-2.05524500
C	-3.01308600	1.61971600	-3.32583700
C	-3.27276500	0.30224100	-3.54891100
C	-2.74098000	-0.41999000	-2.41653700
C	-2.78107900	-1.80419900	-2.26811000
C	-2.25804800	-2.51929200	-1.19349300
C	-2.29040500	-3.95778000	-1.06812300
C	-1.65942600	-4.26013900	0.09973900

C	-1.24974300	-3.00494300	0.68615000
N	-1.62718100	-1.95581000	-0.11483800
C	-0.58111500	-2.88552500	1.90060200
C	-0.21071800	-1.69417500	2.52468500
C	0.40592900	-1.61468300	3.82835400
C	0.55907600	-0.28951700	4.10524200
C	0.04487800	0.42997300	2.96396100
N	-0.41624800	-0.44176000	2.01187100
C	0.03149100	1.81795900	2.84133400
C	-0.42893000	2.52971600	1.73529200
C	-0.39407300	3.96741900	1.61103200
C	-0.91863600	4.26351600	0.38863400
C	-1.28060400	3.00457800	-0.21990200
C	-1.90003300	2.88140500	-1.46376400
H	-2.08722000	3.80025300	-2.01115100
N	-0.96902200	1.96200700	0.61040300
H	-1.06312500	5.23380200	-0.06825000
H	-0.01446600	4.64357300	2.36586500
H	0.42817400	2.39376200	3.67184000
H	0.97803600	0.17279500	4.98947100
H	0.67015600	-2.46802300	4.43915100
H	-0.34947600	-3.80717700	2.42580200
H	-1.48197400	-5.23251800	0.54036800
H	-2.73801400	-4.62959300	-1.78881700
H	-3.25387500	-2.37521400	-3.06117300
H	-3.77220800	-0.15527400	-4.39292400
H	-3.25602200	2.47122800	-3.94793000
S	-3.39840400	0.15722000	1.36634400
H	-4.16591700	-0.45973600	0.44055000
O	0.28730800	-0.14412900	-0.65417700
C	3.21659000	-0.65596000	0.80597400

C	4.53802200	-0.93935100	0.06927000
C	4.37326300	-0.84744700	-1.49012200
C	4.51900200	0.70781700	-1.27801500
C	5.35602000	0.37773900	0.00022900
H	5.26303600	1.07009000	0.84432900
H	6.41506000	0.22366300	-0.21507100
C	3.23433400	1.34846600	-0.80063900
C	2.53251800	0.62567100	0.29323200
H	2.18058100	1.29065500	1.08575800
C	2.79719500	2.52672100	-1.29028300
H	1.89971200	3.00802800	-0.90802500
H	3.33197900	3.04400800	-2.08292100
H	4.98788800	1.28729700	-2.08101000
C	5.58939000	-1.43020600	-2.23086100
H	5.55747800	-1.15481200	-3.29268000
H	5.57654500	-2.52610800	-2.17247400
H	6.54857100	-1.09010600	-1.82982100
C	3.10884900	-1.39803700	-2.15519400
H	3.10354600	-2.49535400	-2.10588800
H	3.09284300	-1.12226100	-3.21821500
H	2.18026200	-1.04013000	-1.70629900
H	5.02650500	-1.83633500	0.46923200
H	3.42169300	-0.55789300	1.88049600
H	2.53766300	-1.51283800	0.70435500
H	1.36939300	0.27076600	-0.16303200

Coordinate III (cis)

Fe	1.48645100	0.04025400	0.23873400
N	2.68771000	-1.26796800	-0.70686200
C	2.70030300	-2.63620900	-0.55706700
C	3.65847700	-3.21854700	-1.46161800

C	4.21058300	-2.19335800	-2.16963500
C	3.59254700	-0.97936200	-1.70120700
C	3.85825800	0.28160400	-2.21546100
C	3.24242400	1.45045100	-1.79585600
C	3.55092100	2.76008300	-2.31224900
C	2.78443400	3.64958100	-1.62126700
C	2.00413000	2.88757700	-0.67922800
N	2.28269800	1.54744400	-0.81486900
C	1.13306800	3.44463100	0.24409000
C	0.40677200	2.72161900	1.17915300
C	-0.52020100	3.30690800	2.11380900
C	-1.06646900	2.28115500	2.82599900
C	-0.47697100	1.06410600	2.32869600
N	0.43468100	1.35562000	1.33888700
C	-0.81749500	-0.20424500	2.77533600
C	-0.28760900	-1.38627800	2.27893200
C	-0.60576300	-2.69721600	2.78395600
C	0.15733600	-3.58676700	2.08803600
C	0.94568700	-2.82399200	1.15471100
C	1.89675100	-3.36847500	0.30459000
H	2.02472500	-4.44558900	0.31772500
N	0.64046600	-1.48630400	1.26746700
H	0.20290500	-4.66320200	2.19087900
H	-1.31651700	-2.89185600	3.57658100
H	-1.55056900	-0.27640600	3.57151600
H	-1.80834300	2.32430800	3.61283900
H	-0.72048000	4.36746000	2.19397400
H	1.01147300	4.52246900	0.23595700
H	2.74341400	4.72658600	-1.72009100
H	4.27038800	2.95431500	-3.09715200
H	4.60116300	0.35789400	-3.00215700

H	4.96024900	-2.23694500	-2.94903100
H	3.86081900	-4.27894900	-1.53872400
S	3.22371500	0.13624100	1.81719700
H	2.66900500	1.02406600	2.67054800
O	0.18657800	-0.10568700	-1.00449100
H	-0.40924600	-0.82286300	-0.71970600
C	-3.26387700	1.19504900	-1.62028700
C	-4.16603600	0.17793100	-2.34485800
C	-4.38927100	-1.11249900	-1.47321800
C	-5.48364700	-0.25080000	-0.72944600
C	-5.64829200	0.54806000	-2.05825400
H	-5.88832500	1.61273800	-1.96554000
H	-6.35105300	0.08424200	-2.75321300
C	-4.83378900	0.67743900	0.28632500
C	-3.74130900	1.41086100	-0.20787800
H	-3.21428600	2.11469400	0.43312100
C	-5.30555100	0.78239300	1.57923100
H	-4.84314900	1.45806700	2.29442600
H	-6.15427800	0.19835500	1.92275300
H	-6.35077800	-0.77644500	-0.31534200
C	-5.01451600	-2.25892700	-2.28487900
H	-5.36268300	-3.05571100	-1.61533000
H	-4.26844000	-2.69666600	-2.96050000
H	-5.86626500	-1.94394400	-2.89453000
C	-3.21836300	-1.68510600	-0.66892900
H	-2.46735500	-2.11364200	-1.34663000
H	-3.56325500	-2.49530000	-0.01351700
H	-2.72864700	-0.93914300	-0.03728000
H	-3.85423900	0.03342800	-3.38600600
H	-3.26644700	2.14837700	-2.17388600
H	-2.21366800	0.86116100	-1.61953100

Coordinate V

Fe	-1.69311300	0.05888800	0.39213000
N	-0.21677700	-0.55247500	1.63078300
C	0.05827400	-1.85265800	2.01293600
C	1.03268100	-1.86832500	3.06987300
C	1.33768900	-0.56859400	3.34772500
C	0.55129900	0.24441400	2.45969600
C	0.56025700	1.63027000	2.45611200
C	-0.20676900	2.41938000	1.61247100
C	-0.15454600	3.85563700	1.58621000
C	-1.01564800	4.26593200	0.61169200
C	-1.59618100	3.08120000	0.04115800
N	-1.10177800	1.95372300	0.66824000
C	-2.50077900	3.08764800	-1.00934600
C	-3.03598400	1.94935400	-1.59090300
C	-3.97094100	1.96370900	-2.68500000
C	-4.28166500	0.66492300	-2.95584200
C	-3.53641100	-0.14453900	-2.02811500
N	-2.76812400	0.65240100	-1.20388000
C	-3.60260200	-1.52776900	-1.97576900
C	-2.88114900	-2.31669000	-1.09296400
C	-2.92312100	-3.75355800	-1.07627400
C	-2.05734900	-4.16398800	-0.10590200
C	-1.48486200	-2.97849400	0.47177500
C	-0.53005700	-2.98740400	1.47745600
H	-0.21756200	-3.95005000	1.86775500
N	-2.00320700	-1.85070100	-0.13405100
H	-1.81048500	-5.17359200	0.19582700
H	-3.53350800	-4.35666600	-1.73574800
H	-4.25421600	-2.02935800	-2.68332400

H	-4.95126900	0.27334900	-3.71057500
H	-4.33201200	2.86077700	-3.17101900
H	-2.80125100	4.04965500	-1.41059100
H	-1.24014400	5.27451400	0.28976300
H	0.47386900	4.45782100	2.22939100
H	1.21843000	2.13283300	3.15677900
H	2.02759100	-0.17969300	4.08528700
H	1.42067000	-2.76648400	3.53246600
S	-3.54260400	0.06566400	2.04851100
H	-3.49070700	1.38926400	2.31720300
C	3.12433500	1.15058700	-1.48698900
C	4.66330800	1.21510600	-1.45465900
C	5.24664100	0.32329900	-0.30056300
C	5.05613600	-0.87614000	-1.31746300
C	5.23916600	0.17353100	-2.45826800
H	4.70176500	0.01024300	-3.39766600
H	6.29043000	0.34718700	-2.69248200
C	3.61986100	-1.34380200	-1.28290000
C	2.62811400	-0.29247500	-1.78487100
H	2.57584000	-0.40161100	-2.87468000
C	3.23524100	-2.55173900	-0.85948400
H	2.18676900	-2.83436400	-0.82884700
H	3.95901500	-3.29349800	-0.52983900
H	5.76214600	-1.71087500	-1.24598600
C	6.73161800	0.61934600	-0.03164500
H	7.17036000	-0.16701800	0.59522100
H	6.83462100	1.56938400	0.50807900
H	7.33333500	0.69141100	-0.94204700
C	4.52501700	0.26677800	1.04730300
H	4.57954200	1.24007900	1.55192800
H	5.00393500	-0.47155900	1.70315000

H	3.47196500	-0.01129100	0.96911900
H	5.02314900	2.24908700	-1.51299800
H	2.72949800	1.82381300	-2.25728800
H	2.70607100	1.48968700	-0.53089200
O	1.28242800	-0.51423500	-1.37044600
H	1.24509600	-0.51114800	-0.39832200

Coordinate I (trans)

Fe	1.50148200	-0.00305600	0.12354000
N	1.52142100	-1.96813400	0.58106100
C	1.02598600	-2.55437800	1.71881500
C	1.13854600	-3.99054700	1.63803700
C	1.70154100	-4.27008700	0.43036700
C	1.93323500	-3.00320900	-0.21988600
C	2.50829200	-2.85645400	-1.47833300
C	2.77119800	-1.64599300	-2.11141300
C	3.40142400	-1.51890000	-3.40308200
C	3.48582000	-0.18567400	-3.66547700
C	2.90487300	0.49573300	-2.53393200
N	2.48093300	-0.40911200	-1.59321200
C	2.78794300	1.87718200	-2.42359100
C	2.23472200	2.56310200	-1.34654900
C	2.13619700	4.00002000	-1.25975000
C	1.54130900	4.27778300	-0.06679300
C	1.28020300	3.00905300	0.56859800
N	1.71073900	1.97585600	-0.22376600
C	0.68625800	2.86138000	1.81822300
C	0.44643800	1.65144000	2.46237300
C	-0.14513800	1.52736000	3.77251700
C	-0.19806700	0.19512100	4.05039800

C	0.36029900	-0.48647700	2.90779900
C	0.48431600	-1.86836000	2.80062000
H	0.12828600	-2.46331400	3.63580100
N	0.75038000	0.41572100	1.95185700
H	-0.57249500	-0.29711900	4.93826800
H	-0.46528300	2.36023400	4.38451900
H	0.39392100	3.76748600	2.33940300
H	1.29801400	5.24157500	0.36073500
H	2.48376500	4.68766000	-2.01938600
H	3.16386600	2.47287400	-3.24946500
H	3.89848400	0.30854800	-4.53514600
H	3.73111100	-2.35118800	-4.01086500
H	2.78750900	-3.76305500	-2.00574500
H	1.94729000	-5.23398000	0.00454400
H	0.82332800	-4.67667100	2.41302200
S	3.78701500	0.12455200	1.20665200
H	4.30560200	-1.00989800	0.68719500
O	0.02635200	-0.07796000	-0.58453100
C	-4.00819500	1.45258900	-1.54116700
C	-4.67359400	0.16208100	-2.04934800
C	-3.81944000	-1.06062800	-1.60874300
C	-4.58712200	-0.98303000	-0.25237700
C	-5.78448200	-0.33235800	-1.05620900
C	-3.94137800	0.06038000	0.62563900
C	-3.35006000	1.23663200	-0.14638200
H	-3.39258200	2.15122900	0.45605200
C	-3.88051100	-0.04338000	1.95861800
H	-3.43414900	0.73420700	2.57544400
H	-4.27173000	-0.91496900	2.47941100
H	-4.79711200	-1.91026800	0.29304800
H	-4.95680400	0.24578800	-3.10587500

H	-4.74955400	2.25954800	-1.50181500
H	-3.23976700	1.77384400	-2.25542600
H	-2.28421800	1.02338900	-0.29933900
H	-4.05269900	-1.96965900	-2.16647300
H	-2.73191900	-0.93620000	-1.57915600
C	-6.66651700	0.69768500	-0.34539500
H	-7.32657400	1.19986900	-1.06479300
H	-7.30619100	0.20239300	0.39668200
H	-6.09544500	1.46682600	0.18070500
C	-6.69678400	-1.39601000	-1.68744800
H	-7.30254500	-1.88914100	-0.91653100
H	-7.38646400	-0.92814800	-2.40192100
H	-6.14541900	-2.17420400	-2.22347700

Coordinate II (trans) – TS (-1823.33)

Fe	-1.28190100	-0.00908700	0.19898600
N	-2.16931200	1.02573700	-1.28449900
C	-2.16472500	2.38949800	-1.44042700
C	-2.85579600	2.76130100	-2.65367600
C	-3.27877100	1.60381600	-3.23195900
C	-2.83956700	0.52936800	-2.37236500
C	-3.05575200	-0.82402800	-2.62033000
C	-2.62970500	-1.87424300	-1.81081400
C	-2.85077800	-3.27348100	-2.09278000
C	-2.26657600	-3.97057100	-1.07979500
C	-1.69549300	-2.99408200	-0.18091900
N	-1.93061200	-1.72276200	-0.64144500
C	-1.01498600	-3.30785100	0.99166000
C	-0.48791600	-2.39831900	1.90865400
C	0.14368800	-2.76959400	3.15335700
C	0.47443700	-1.60747900	3.78269600

C	0.05141200	-0.53532500	2.91345000
N	-0.52929400	-1.03616100	1.77724400
C	0.21695800	0.82065900	3.18816700
C	-0.15670700	1.86912100	2.34986500
C	0.05670600	3.26752100	2.63821900
C	-0.43395100	3.96175800	1.57312100
C	-0.95285200	2.98222200	0.64718400
C	-1.58822600	3.29467000	-0.55465400
H	-1.66159600	4.34722400	-0.81109600
N	-0.76911800	1.71572200	1.13303600
H	-0.45985600	5.03239400	1.41763700
H	0.52071600	3.64867100	3.53851000
H	0.69038500	1.08234200	4.12950600
H	0.95838000	-1.47118000	4.74094300
H	0.29667200	-3.78723300	3.48819500
H	-0.90505200	-4.36189900	1.22785600
H	-2.21912300	-5.04209900	-0.93568000
H	-3.38220700	-3.65198700	-2.95615000
H	-3.59850700	-1.08255300	-3.52433600
H	-3.83354600	1.47040300	-4.15144700
H	-2.99152700	3.77804500	-2.99851800
S	-3.41326300	0.09544900	1.43754600
H	-4.24984000	-0.20232900	0.41861500
O	0.20195300	-0.09436300	-0.71857200
C	3.12057900	-1.11815900	0.35781500
C	3.98135400	-1.42950400	-0.87868000
C	3.28505000	-0.83276400	-2.13427900
C	3.98570900	0.51003900	-1.76155900
C	5.11368800	-0.35848700	-1.07121500
C	3.17152500	1.21027900	-0.69772400
C	2.52645600	0.29859600	0.27535300

H	2.31483000	0.75921700	1.24317600
C	3.03864200	2.55249900	-0.64502900
H	2.46450600	3.03868500	0.14063900
H	3.50343300	3.19796300	-1.38628100
H	4.29248300	1.19107900	-2.56304200
H	4.28433900	-2.48332200	-0.89930700
H	3.71848700	-1.22281900	1.27378300
H	2.31160400	-1.85567100	0.43739000
H	1.32547400	0.13440800	-0.17375600
H	3.65731300	-1.25034400	-3.07174000
H	2.19094300	-0.83637000	-2.14878300
C	5.82594900	0.20740800	0.16035000
H	6.44355700	-0.56672600	0.63409100
H	6.49426100	1.02837700	-0.13014100
H	5.13970600	0.59782800	0.91629800
C	6.18062900	-0.81641600	-2.07800600
H	6.82079000	0.02626200	-2.36846400
H	6.82477300	-1.58131100	-1.62544800
H	5.75709100	-1.24151500	-2.99267800

Coordinate III (trans)

Fe	-1.45890100	0.02709200	0.15179600
N	-1.67982300	1.93069700	-0.45795800
C	-1.26014800	3.06406000	0.20157700
C	-1.53681700	4.22880800	-0.60033900
C	-2.10705200	3.78830100	-1.75659100
C	-2.18354800	2.35227700	-1.66627300
C	-2.67283700	1.53248000	-2.67275300
C	-2.72638000	0.14868600	-2.60589000
C	-3.27433400	-0.69349200	-3.63944000
C	-3.19832000	-1.97624800	-3.18689500

C	-2.60231300	-1.92375700	-1.87551000
N	-2.30545000	-0.62122900	-1.54742000
C	-2.39554300	-3.03116400	-1.06769400
C	-1.84696000	-2.98866600	0.20627300
C	-1.59867500	-4.15016300	1.02176300
C	-1.00984400	-3.71060800	2.16942900
C	-0.89679100	-2.27841200	2.06127500
N	-1.42790800	-1.85632700	0.86398600
C	-0.30973000	-1.46782000	3.02189500
C	-0.17016700	-0.09146600	2.91803300
C	0.38056200	0.74974100	3.94965600
C	0.28422000	2.03575800	3.50691600
C	-0.32232000	1.98538500	2.20115400
C	-0.63474600	3.10154300	1.43874000
H	-0.37077200	4.07450000	1.83915800
N	-0.57000600	0.67865700	1.84954200
H	0.58125000	2.94716700	4.00942100
H	0.77383600	0.38662700	4.89033200
H	0.06025400	-1.94415600	3.92347400
H	-0.66828600	-4.28862500	3.01831000
H	-1.84056800	-5.16445000	0.73173500
H	-2.68115100	-4.00132500	-1.46002100
H	-3.51706200	-2.88641800	-3.67827900
H	-3.66877500	-0.33027500	-4.57961900
H	-3.04218100	2.00836400	-3.57480800
H	-2.44279400	4.36615700	-2.60788700
H	-1.30605600	5.24391800	-0.30412500
S	-3.55716600	0.15054700	1.21660700
H	-3.86520000	1.42762000	0.90312300
O	0.15255200	-0.13704200	-0.62672000
C	3.53926400	-1.66816700	-0.07947400

C	4.28570300	-1.32858300	-1.38361500
C	3.54654000	-0.15042600	-2.07751800
C	4.36686100	0.78363400	-1.13638500
C	5.47893300	-0.33844000	-1.11794300
C	3.64897700	0.83788000	0.20365900
C	3.21762300	-0.41097200	0.68786700
H	2.71147500	-0.48558800	1.64880400
C	3.45104100	2.02855800	0.87511300
H	2.93465800	2.05743800	1.83100600
H	3.80453300	2.97176800	0.46926800
H	4.65209000	1.77911200	-1.49293500
H	4.50050900	-2.23032400	-1.96892300
H	4.13456700	-2.35239300	0.54590900
H	2.61377100	-2.21721600	-0.31523500
H	0.85450300	0.08872000	0.01445300
H	3.81410900	-0.03579300	-3.12969100
H	2.45678700	-0.11678600	-1.97764100
C	6.33654000	-0.51915700	0.13783800
H	6.93575400	-1.43572300	0.06102300
H	7.03531800	0.32060500	0.24597200
H	5.74754500	-0.57867200	1.05648100
C	6.42135900	-0.23079400	-2.32796400
H	7.10338400	0.62030400	-2.20629900
H	7.03477100	-1.13702300	-2.41231900
H	5.89509500	-0.09957700	-3.27781400

Coordinate V (trans)

Fe	1.47893300	-0.04784500	0.22564300
N	0.87630600	-1.60018500	1.35522800
C	0.44222300	-1.55333200	2.66232500
C	0.22090600	-2.88559900	3.16268900

C	0.53318200	-3.74426400	2.15146200
C	0.94730800	-2.93771100	1.03224900
C	1.37240300	-3.45003000	-0.18467200
C	1.78030500	-2.68045500	-1.26472300
C	2.18952200	-3.22157000	-2.53331700
C	2.47943000	-2.16174900	-3.34110500
C	2.24653500	-0.97075400	-2.56835700
N	1.83012100	-1.30213400	-1.29534300
C	2.39796300	0.31739300	-3.06192300
C	2.13591700	1.47439300	-2.34356900
C	2.30693300	2.80729100	-2.85919700
C	1.98024100	3.66652600	-1.85222300
C	1.60980100	2.86060300	-0.71871500
N	1.69376300	1.52049200	-1.03725900
C	1.24124600	3.36999000	0.51771600
C	0.90081300	2.59832600	1.62040400
C	0.49293500	3.14003000	2.88906600
C	0.21766600	2.08063000	3.70292000
C	0.45538900	0.88918600	2.93321800
C	0.25265200	-0.39903400	3.40753500
H	-0.08939300	-0.51018200	4.43109400
N	0.88087300	1.21893600	1.66210700
H	-0.12459600	2.08966400	4.72965500
H	0.42361800	4.19708800	3.11092500
H	1.21448400	4.44859600	0.63112100
H	1.98717000	4.74883500	-1.85953200
H	2.63715300	3.03911800	-3.86354600
H	2.73825900	0.42620900	-4.08631700
H	2.81094500	-2.16919100	-4.37138700
H	2.23461900	-4.27809300	-2.76381500
H	1.38161500	-4.52842200	-0.30284600

H	0.49434200	-4.82591500	2.14748900
H	-0.12767100	-3.11609800	4.16111600
S	3.85294000	-0.03812300	1.05586200
H	4.28988600	-1.03556000	0.25536200
C	-2.80352200	-1.06486100	0.26505700
C	-3.99687500	-1.33326200	-0.66747000
C	-5.11445700	-0.24453600	-0.49020500
C	-4.23048500	0.62430100	-1.47500600
C	-3.70190400	-0.71983500	-2.06485800
H	-2.66593300	-0.73845400	-2.41187600
H	-4.35108000	-1.11063800	-2.85052800
C	-3.12804300	1.29175700	-0.69597500
C	-2.18291400	0.33477300	0.03358200
C	-2.94977900	2.61634900	-0.62707700
H	-2.16498600	3.05799300	-0.01538300
H	-3.58232200	3.30671600	-1.18047200
H	-4.75417700	1.32769200	-2.13188100
C	-6.44429300	-0.66495000	-1.13511700
H	-7.12469300	0.19303900	-1.20547100
H	-6.93690800	-1.42956000	-0.52085600
H	-6.32491800	-1.07709600	-2.14133100
C	-5.41103500	0.30696100	0.90713400
H	-5.87174000	-0.46702100	1.53444700
H	-6.11914700	1.14307600	0.84294000
H	-4.52337200	0.67489900	1.42887900
H	-4.31256900	-2.38189100	-0.61253100
H	-2.01619100	-1.80726300	0.09514600
H	-3.11083400	-1.16578700	1.31206300
H	-1.87378600	0.77819700	0.98730400
O	-0.98841900	0.13980900	-0.77221300
H	-0.73753500	1.00632100	-1.13679300

Delta Site – Sextet state

Coordinate I (cis)

Fe	-1.41563300	0.01315200	0.11997500
N	-0.80922400	-0.82611200	1.92806100
C	-0.64446700	-2.15569800	2.19524600
C	-0.11328300	-2.31794100	3.53301900
C	0.03425500	-1.06440400	4.05329900
C	-0.40653700	-0.13359500	3.03513600
C	-0.42558700	1.26435700	3.15844600
C	-0.85700200	2.21022900	2.21655200
C	-0.86758900	3.64673900	2.40326800
C	-1.35992000	4.19151800	1.25288100
C	-1.65224600	3.08963500	0.35922700
N	-1.33869000	1.90961700	0.97311500
C	-2.18930400	3.19760500	-0.93267300
C	-2.51638000	2.15880700	-1.81794700
C	-3.09484900	2.31823000	-3.13593800
C	-3.25915000	1.06431500	-3.64947500
C	-2.78116500	0.13448300	-2.64723600
N	-2.34407200	0.82867200	-1.55374500
C	-2.76350800	-1.26332300	-2.76925300
C	-2.31947300	-2.20798500	-1.83146200
C	-2.34692000	-3.64621700	-2.00309400
C	-1.83845000	-4.18990300	-0.85921500
C	-1.49887600	-3.08496200	0.01393900
C	-0.95971800	-3.19396500	1.30494900
H	-0.77391500	-4.20277300	1.66221800
N	-1.80124600	-1.90512000	-0.60405300
H	-1.70554200	-5.23626000	-0.61671900

H	-2.71276900	-4.15920000	-2.88295300
H	-3.14734200	-1.66397700	-3.70322300
H	-3.66503600	0.78318100	-4.61249700
H	-3.34036100	3.26702500	-3.59496700
H	-2.38634100	4.20571600	-1.28581200
H	-1.51786800	5.23686100	1.02131100
H	-0.54175000	4.15809000	3.29966700
H	-0.06864500	1.66296200	4.10388400
H	0.40154600	-0.78472800	5.03209300
H	0.10821100	-3.26777000	4.00202900
O	0.09868300	0.09294500	-0.51778700
S	-3.71437500	-0.11392600	1.10714500
H	-4.17680000	1.06867700	0.64645200
C	3.77994500	-0.63085400	-2.19214700
C	5.11503500	0.10602700	-1.99355600
C	5.06412400	1.04508700	-0.73651300
C	5.39073800	-0.26485700	0.08804500
C	6.08789300	-0.80847500	-1.19779500
H	6.03617200	-1.88677000	-1.38274300
H	7.13067600	-0.49359100	-1.27058700
C	4.12091900	-1.04148800	0.33196500
C	3.40834900	-1.48602500	-0.94390200
H	3.69858400	-2.52714100	-1.13916100
H	2.32417500	-1.49541200	-0.79021300
C	3.66845600	-1.33144100	1.55719600
H	2.73902300	-1.87567700	1.71383700
H	4.21953100	-1.04110700	2.44934800
H	5.98346700	-0.14479900	1.00215600
C	6.23165500	2.04428200	-0.70971300
H	6.30664200	2.52095400	0.27599000
H	6.06833700	2.83809300	-1.45012100

H	7.19945500	1.58345000	-0.92919500
C	3.77235900	1.80466100	-0.42113300
H	3.59023500	2.57956300	-1.17755900
H	3.85699400	2.30916000	0.55041900
H	2.88887100	1.16258100	-0.37924600
H	5.46941700	0.55255500	-2.93087200
H	3.85153100	-1.28497800	-3.06988700
H	2.98728800	0.09416700	-2.41134300

Coordinate (cis) – TS (-1896.64)

Fe	1.21189900	0.02333000	0.20323800
N	0.33919400	0.21406400	2.08228300
C	0.06834500	1.38781200	2.72532800
C	-0.54323200	1.10618600	4.00860400
C	-0.61902200	-0.25286600	4.11978600
C	-0.05733300	-0.80387200	2.90301400
C	0.07203000	-2.17344900	2.60840000
C	0.62097200	-2.76565800	1.45883800
C	0.73271700	-4.18778400	1.19715500
C	1.33431000	-4.31671000	-0.02226000
C	1.59422000	-2.97287500	-0.50379400
N	1.14686000	-2.06766700	0.41094400
C	2.23184900	-2.63242600	-1.71047000
C	2.54578500	-1.35379000	-2.19949100
C	3.22295700	-1.07238300	-3.45056400
C	3.32811500	0.28463700	-3.54772500
C	2.71495600	0.83962900	-2.35687900
N	2.25770500	-0.17627200	-1.56569200
C	2.60402000	2.20743800	-2.05265600
C	2.03030800	2.79900500	-0.91494800

C	1.94652400	4.22044100	-0.63715100
C	1.31681100	4.34971200	0.56701200
C	1.01468700	3.00765700	1.02844400
C	0.36822100	2.66948100	2.22740300
H	0.07583700	3.50212000	2.86150400
N	1.46250100	2.10112900	0.11041600
H	1.07565800	5.26031700	1.10017000
H	2.32275900	5.00386300	-1.28235700
H	3.01725400	2.89155200	-2.78870100
H	3.77657500	0.86568600	-4.34315400
H	3.56769900	-1.82163300	-4.15150900
H	2.53245900	-3.46506100	-2.34072400
H	1.58685900	-5.22771600	-0.54937700
H	0.39487600	-4.97218200	1.86210700
H	-0.29651700	-2.85774500	3.36771700
H	-1.01122200	-0.83600900	4.94294000
H	-0.86029600	1.85432100	4.72363200
S	3.34257800	-0.04851900	1.40041100
H	4.11277800	0.54492800	0.46176600
O	-0.29207700	0.09659100	-0.69120900
C	-3.26118400	0.42753000	0.83886800
C	-4.58583300	0.74413400	0.12144700
C	-4.40320000	0.83155400	-1.43602600
C	-4.49467700	-0.74204000	-1.39922800
C	-5.35500200	-0.58478600	-0.10347000
H	-5.24505000	-1.36291800	0.65999100
H	-6.41673800	-0.44444200	-0.31444400
C	-3.19309900	-1.38688200	-0.97688800
C	-2.52733000	-0.76289800	0.19474400
H	-2.14544700	-1.49482900	0.91108900
C	-2.70868200	-2.48968300	-1.58516600

H	-1.79745600	-2.97673100	-1.24512700
H	-3.21630800	-2.93649200	-2.43624000
H	-4.93456400	-1.24490400	-2.26751500
C	-5.63252600	1.44895500	-2.12521800
H	-5.57788400	1.29648500	-3.21059800
H	-5.66255900	2.53094000	-1.94384900
H	-6.58235900	1.03033200	-1.78011000
C	-3.15357300	1.49822800	-2.01775800
H	-3.19370500	2.58341900	-1.85291600
H	-3.11215800	1.33733900	-3.10338000
H	-2.21710200	1.13132600	-1.59318000
H	-5.10976300	1.57346800	0.61209600
H	-3.47138600	0.20087400	1.89283300
H	-2.61495400	1.31522800	0.84445000
H	-1.36793000	-0.32912100	-0.21996000

Coordinate III (cis)

Fe	1.17240200	0.04266800	-0.03596400
N	0.99719100	-1.75620200	1.01870500
C	0.12944400	-2.00780700	2.05921400
C	0.08490100	-3.42594700	2.32244900
C	0.93364200	-4.02323300	1.43525700
C	1.50666000	-2.97346500	0.62755300
C	2.45801400	-3.16499400	-0.37697700
C	3.09174000	-2.16873400	-1.11533000
C	4.08862400	-2.39128100	-2.13412700
C	4.44151000	-1.16707600	-2.62473600
C	3.66172500	-0.18397500	-1.91228500
N	2.85735500	-0.81381100	-0.98415500
C	3.70639000	1.19191900	-2.12798200
C	2.96559900	2.16167200	-1.44694100

C	3.08265600	3.58662000	-1.64754000
C	2.21937200	4.18345900	-0.77425800
C	1.56940000	3.12638700	-0.03657600
N	2.03492000	1.90513100	-0.46837900
C	0.63605000	3.31638200	0.98270900
C	0.03493100	2.31929100	1.75297300
C	-0.88187600	2.55353800	2.84364600
C	-1.22153400	1.33037900	3.34731800
C	-0.51478700	0.34309600	2.56597100
C	-0.57788500	-1.03716500	2.76765900
H	-1.22250000	-1.38689100	3.56815900
N	0.24216000	0.96753500	1.60305600
H	-1.88066700	1.10691100	4.17639700
H	-1.20649600	3.53010600	3.17943800
H	0.37107500	4.34255300	1.21836000
H	2.03931200	5.24076100	-0.62771000
H	3.74942800	4.05839000	-2.35790400
H	4.39608800	1.54689200	-2.88763600
H	5.16266700	-0.93965300	-3.39937600
H	4.46458600	-3.36291500	-2.42814900
H	2.75122500	-4.18982900	-0.58330500
H	1.16835900	-5.07545500	1.33789600
H	-0.51353400	-3.89246900	3.09445600
S	4.54762700	-0.29752900	1.57590900
H	4.95573400	0.65656800	0.70993700
O	-0.13824900	-0.07180800	-1.33158900
H	-0.80070100	-0.76620600	-1.17815700
C	-3.60890200	1.32372500	-1.36546200
C	-4.56781900	0.43059100	-2.17539700
C	-4.69844700	-0.99977200	-1.53356100
C	-5.72434400	-0.31133200	-0.55008000

C	-6.01901900	0.70775900	-1.69263000
H	-6.25684800	1.73271300	-1.38823400
H	-6.78075700	0.35877300	-2.39260200
C	-4.98768600	0.43538900	0.55258300
C	-3.95067200	1.26966300	0.10095600
H	-3.36909200	1.85781300	0.80805500
C	-5.33645600	0.29610800	1.88090200
H	-4.81237500	0.84258400	2.66097500
H	-6.14547800	-0.35947200	2.18933100
H	-6.54476600	-0.92314300	-0.15990800
C	-5.38798700	-1.99773900	-2.47826900
H	-5.66615800	-2.90958200	-1.93467600
H	-4.70503500	-2.28936100	-3.28651600
H	-6.29523900	-1.59938800	-2.94141800
C	-3.45322100	-1.67850100	-0.95453800
H	-2.76516700	-1.95947800	-1.76374600
H	-3.72906500	-2.60220800	-0.42964500
H	-2.91525200	-1.04592800	-0.24300300
H	-4.35457500	0.48160400	-3.24963800
H	-3.67370300	2.36064400	-1.73426700
H	-2.56106800	1.02291800	-1.52348100

Coordinate V (cis)

Fe	1.77992000	-0.06959400	0.52495000
N	0.13589700	0.57139600	1.66172000
C	-0.12500600	1.87541100	2.03005600
C	-1.09190500	1.89467300	3.09964300
C	-1.39977900	0.59482900	3.38467300
C	-0.62503200	-0.22917700	2.48957200
C	-0.63996300	-1.62339700	2.46352700
C	0.10694100	-2.44242000	1.61592200

C	0.02310200	-3.88117300	1.56763700
C	0.87792100	-4.29846300	0.58725400
C	1.49120700	-3.11773100	0.03128700
N	1.01570500	-1.99823800	0.67946100
C	2.40378700	-3.10910900	-1.02388300
C	2.97192600	-1.98079600	-1.61397300
C	3.89440300	-1.99855800	-2.72462500
C	4.21240100	-0.70005100	-2.99922200
C	3.48561500	0.11989500	-2.05889100
N	2.73466900	-0.68081200	-1.22777100
C	3.53052900	1.51277700	-2.00715300
C	2.81335800	2.33133700	-1.13451500
C	2.83060600	3.77340500	-1.14723100
C	1.96735200	4.19214300	-0.17507300
C	1.41626900	3.00875600	0.43796300
C	0.46558500	3.00239000	1.45904400
H	0.14747500	3.96826900	1.83891800
N	1.95320600	1.88595000	-0.15405700
H	1.71179800	5.20699500	0.10145400
H	3.42113300	4.37795300	-1.82360700
H	4.16845200	2.00977600	-2.73146500
H	4.87198200	-0.32005000	-3.76885100
H	4.24167000	-2.89371200	-3.22448400
H	2.68397500	-4.07302700	-1.43722600
H	1.07444100	-5.31028900	0.25658200
H	-0.61873000	-4.48392100	2.19726600
H	-1.30621500	-2.11948900	3.16240600
H	-2.08788600	0.21803200	4.13045700
H	-1.47808500	2.79165400	3.56652900
S	3.56005200	-0.06158400	2.12978100
H	3.48802200	-1.38337700	2.40282200

C	-2.98317900	-1.13666400	-1.48053600
C	-4.52219900	-1.21297800	-1.48028700
C	-5.13702900	-0.31608600	-0.34658600
C	-4.93358800	0.87619900	-1.36930900
C	-5.08536000	-0.18454500	-2.50454600
H	-4.53074000	-0.02521900	-3.43451800
H	-6.13036300	-0.36852700	-2.75809700
C	-3.50144500	1.35456700	-1.30825800
C	-2.49275400	0.30705900	-1.78449600
H	-2.43003600	0.40553500	-2.87495200
C	-3.13437400	2.56834700	-0.88624700
H	-2.08860800	2.85777100	-0.83562000
H	-3.86997800	3.30750800	-0.57740500
H	-5.64702100	1.70627500	-1.31977400
C	-6.62509200	-0.62175900	-0.10681600
H	-7.08327600	0.16643800	0.50365100
H	-6.73208100	-1.56784400	0.43909200
H	-7.20686400	-0.70666300	-1.02901000
C	-4.44481900	-0.24235000	1.01556800
H	-4.49997600	-1.21243500	1.52635000
H	-4.94513400	0.49567000	1.65567800
H	-3.39348600	0.04621700	0.95729400
H	-4.87288500	-2.25022500	-1.53718900
H	-2.56681800	-1.81498600	-2.23492900
H	-2.58323600	-1.46239300	-0.51208300
O	-1.15440000	0.54430700	-1.35952000
H	-1.11429400	0.52713800	-0.38678700

Coordinate I (trans)

Fe	1.46514200	-0.00013600	0.11637700
N	1.77738300	-2.05255500	0.25108300

C	1.37905100	-2.86048900	1.27975100
C	1.66431600	-4.24058200	0.94749600
C	2.23141400	-4.23944900	-0.29419400
C	2.29525000	-2.85862600	-0.72464600
C	2.81273500	-2.40347100	-1.94759600
C	2.91205000	-1.07911800	-2.39975700
C	3.48670200	-0.65021000	-3.65874900
C	3.39620700	0.71100700	-3.69051300
C	2.76579200	1.11884900	-2.45128800
N	2.48875600	0.01337800	-1.69684400
C	2.48485100	2.43902700	-2.06961800
C	1.89736000	2.88942900	-0.87687700
C	1.64813200	4.27258000	-0.52837800
C	1.07145200	4.27079100	0.70904500
C	0.96561600	2.88646000	1.11976900
N	1.47422700	2.08052100	0.14046700
C	0.43321900	2.42861400	2.33522500
C	0.32652700	1.10265100	2.78226500
C	-0.20065000	0.67949400	4.06393900
C	-0.10102000	-0.68099600	4.10116800
C	0.48770600	-1.09199100	2.84268300
C	0.78378100	-2.41149500	2.46876300
H	0.52726900	-3.18056600	3.19178300
N	0.73007800	0.00888900	2.07117500
H	-0.39020200	-1.35191300	4.89956000
H	-0.58686500	1.34358000	4.82617500
H	0.06910000	3.19371800	3.01501700
H	0.74748600	5.11906900	1.29782500
H	1.88936900	5.12278100	-1.15299300
H	2.76426900	3.20994500	-2.78213000
H	3.72426300	1.38543800	-4.47073500

H	3.90368300	-1.31088200	-4.40761000
H	3.19129000	-3.16851400	-2.61953200
H	2.57868800	-5.08583800	-0.87231400
H	1.45527500	-5.08827500	1.58693400
S	3.72993900	0.25462800	1.15093600
H	4.27962100	-0.88620900	0.68069600
O	-0.01909100	-0.13359400	-0.58165700
C	-3.91981300	1.22412300	-1.74531700
C	-4.56694800	-0.13384100	-2.06763900
C	-3.76268900	-1.26275700	-1.36213600
C	-4.62174900	-0.94880300	-0.09771200
C	-5.75096300	-0.44562500	-1.08498900
C	-4.02500100	0.23110100	0.62928500
C	-3.36244200	1.25191900	-0.29156300
H	-3.42975800	2.25775300	0.13849400
C	-4.06148400	0.36309900	1.96058300
H	-3.65170000	1.23777300	2.46210700
H	-4.50099100	-0.40275900	2.59629700
H	-4.88250900	-1.76630200	0.58423500
H	-4.77503200	-0.23468600	-3.14003300
H	-4.65043100	2.02731800	-1.89852400
H	-3.09928800	1.41461400	-2.44839400
H	-2.29279100	1.00859900	-0.33167100
H	-3.96975200	-2.25459900	-1.76882500
H	-2.67819200	-1.13665600	-1.27798800
C	-6.66548200	0.69402400	-0.62799100
H	-7.26742000	1.06386700	-1.46840000
H	-7.36150700	0.33703100	0.14225400
H	-6.12128700	1.54233700	-0.20519400
C	-6.63211500	-1.60123200	-1.58523400
H	-7.29669000	-1.95189600	-0.78534300

H	-7.26404400	-1.26340800	-2.41685600
H	-6.05580000	-2.46162100	-1.93816900

Coordinate II (trans) – TS (-1860.77)

Fe	-1.27238900	-0.02234300	0.18745200
N	-1.48352100	2.06439800	-0.04448600
C	-1.03455100	3.02295700	0.81572300
C	-1.32295600	4.33619700	0.26958200
C	-1.94753000	4.13478800	-0.92768700
C	-2.04198900	2.69827100	-1.11257000
C	-2.62492400	2.03366600	-2.20494100
C	-2.76065200	0.65008900	-2.40951200
C	-3.39657900	0.03116600	-3.55556700
C	-3.32340700	-1.31834800	-3.36894200
C	-2.64188800	-1.53257900	-2.10788900
N	-2.31752900	-0.32328900	-1.55563400
C	-2.36158900	-2.78624100	-1.53821300
C	-1.72362200	-3.06210000	-0.31722800
C	-1.46610000	-4.37543300	0.24440600
C	-0.83188000	-4.17521000	1.43666500
C	-0.70161600	-2.73955100	1.60446900
N	-1.25248300	-2.10475400	0.52950500
C	-0.11183400	-2.07610200	2.69097100
C	0.02197900	-0.68985500	2.89677100
C	0.61585600	-0.07228800	4.06374700
C	0.53089100	1.27909500	3.88267600
C	-0.11387000	1.49080100	2.60369500
C	-0.40257900	2.74686300	2.03775300
H	-0.10169300	3.60899000	2.62669900
N	-0.40119700	0.28179100	2.03282500
H	0.86502300	2.06547600	4.54698600

H	1.03234900	-0.60933000	4.90604800
H	0.28501500	-2.70940800	3.47972400
H	-0.48267400	-4.91939600	2.14083900
H	-1.73808400	-5.31549700	-0.21815000
H	-2.68649200	-3.64873900	-2.11365900
H	-3.69410700	-2.10445700	-4.01391500
H	-3.83858300	0.56915000	-4.38418500
H	-3.03255300	2.66774000	-2.98755800
H	-2.31397500	4.87853600	-1.62351000
H	-1.07749300	5.27699200	0.74526900
S	-3.39147400	0.02766300	1.41338500
H	-4.22359900	-0.29757400	0.39937200
O	0.22745200	-0.06570700	-0.71561300
C	3.15106800	-1.03980600	0.40910100
C	4.02084400	-1.37960600	-0.81345400
C	3.32307100	-0.83142700	-2.09020600
C	4.00554900	0.53104000	-1.75741700
C	5.14084100	-0.30114400	-1.03498600
C	3.17762100	1.25380900	-0.71972600
C	2.54297400	0.36688200	0.28081600
H	2.32146800	0.85535600	1.23264700
C	3.02332700	2.59511200	-0.71229900
H	2.43657000	3.09690700	0.05388400
H	3.48029700	3.22345800	-1.47272200
H	4.30734400	1.19035300	-2.57862100
H	4.33667300	-2.42968200	-0.79935700
H	3.74559300	-1.10876700	1.33070900
H	2.35044400	-1.78424700	0.50970500
H	1.34005000	0.17710700	-0.16898500
C	5.84052200	0.31227600	0.18098500
H	6.46468300	-0.43905300	0.68201600

H	6.50072200	1.13122300	-0.13274900
H	5.14635900	0.71871600	0.92112100
C	6.21795300	-0.77717700	-2.02235800
H	6.86966200	-1.51896500	-1.54302000
H	5.80399400	-1.23642100	-2.92481200
H	6.84861400	0.06406300	-2.33672600
H	3.70482200	-1.27380800	-3.01229600
H	2.22918400	-0.84863000	-2.10940000

Coordinate III (trans)

Fe	-1.12068200	-0.07210800	-0.00282900
N	-2.60871100	0.89292600	-1.15943700
C	-2.71755800	2.25831600	-1.33356700
C	-3.55703500	2.53596900	-2.47373700
C	-3.93494700	1.33266700	-2.99635800
C	-3.33246300	0.30738500	-2.17920600
C	-3.45558300	-1.06480200	-2.38633700
C	-2.90093200	-2.07446600	-1.59566500
C	-3.10555800	-3.48980100	-1.79265600
C	-2.42731600	-4.13231300	-0.79678300
C	-1.80083900	-3.11346100	0.01128400
N	-2.10111900	-1.86910600	-0.49573400
C	-1.02677300	-3.35321100	1.14690500
C	-0.43850700	-2.38962100	1.96810600
C	0.31423600	-2.67015500	3.16819800
C	0.70097100	-1.46731100	3.68527900
C	0.18683400	-0.44604700	2.80304400
N	-0.50184100	-1.03049900	1.76689900
C	0.35115800	0.92891800	2.98342700
C	-0.17426100	1.93639900	2.17452900
C	-0.03564100	3.35275900	2.41598400

C	-0.72102300	3.99511200	1.42455600
C	-1.28561100	2.97541100	0.57339500
C	-2.09688000	3.21815500	-0.53730800
H	-2.27846400	4.25795400	-0.79167600
N	-0.93157300	1.73110200	1.04222200
H	-0.85156600	5.06025700	1.28241000
H	0.50643600	3.78810500	3.24576400
H	0.92224900	1.24498900	3.85098800
H	1.27335600	-1.27709000	4.58421100
H	0.50647700	-3.66086100	3.55976000
H	-0.88453200	-4.39125700	1.43167700
H	-2.35742000	-5.19678300	-0.61273700
H	-3.70198700	-3.92387400	-2.58495400
H	-4.06043500	-1.38117700	-3.23062600
H	-4.56548900	1.14568900	-3.85623700
H	-3.81610200	3.52745100	-2.82259400
S	-4.70710700	0.44140400	1.10885800
H	-4.18234300	-0.79537700	0.95882100
O	0.32968500	-0.13705800	-1.12514700
C	4.60535100	-0.76271100	0.91506300
C	5.13424600	-1.24391400	-0.44883000
C	3.94970700	-1.26348100	-1.45502100
C	4.23372200	0.25558500	-1.66244100
C	5.73501600	-0.05054300	-1.28006700
C	3.53866100	1.00527300	-0.53610300
C	3.72988900	0.45002600	0.74313000
H	3.25895900	0.89992000	1.61431400
C	2.76887100	2.12850300	-0.77360300
H	2.28185200	2.66078500	0.03979200
H	2.66240200	2.53919500	-1.77348400
H	4.03719200	0.70461000	-2.64174800

H	5.74898700	-2.14560700	-0.34595300
H	5.43709500	-0.53832600	1.60190400
H	4.03773900	-1.57283100	1.40087200
H	1.05450300	0.49319600	-0.94646400
H	4.15153400	-1.86108200	-2.34593300
H	2.96051500	-1.52490900	-1.06497400
C	6.55631900	1.02419300	-0.56203200
H	7.51114300	0.60681100	-0.21697600
H	6.78845700	1.84811400	-1.24928700
H	6.04353700	1.45086800	0.30373200
C	6.54480500	-0.54049700	-2.49175200
H	6.75901500	0.29441000	-3.17090800
H	7.50635800	-0.95424300	-2.16199100
H	6.03403600	-1.31510400	-3.07094500

Coordinate V (trans)

Fe	-1.94003500	0.06607200	0.29143800
N	-2.30161400	-0.37599000	-1.71699300
C	-2.58026700	0.54076600	-2.70623500
C	-3.06442200	-0.14261400	-3.88209800
C	-3.07719700	-1.47596500	-3.59107300
C	-2.60151700	-1.61592600	-2.23522300
C	-2.46063400	-2.82455700	-1.55471500
C	-1.97643200	-2.98789600	-0.25681600
C	-1.78677800	-4.25591000	0.40343300
C	-1.26216400	-3.98992600	1.63605400
C	-1.12610000	-2.55783000	1.73614400
N	-1.57731100	-1.96524600	0.57626500
C	-0.58907300	-1.87919600	2.83024600
C	-0.38725200	-0.50335000	2.93036600
C	0.17062100	0.17360000	4.07499400

C	0.18852900	1.50707000	3.78146300
C	-0.35768400	1.65498600	2.45475200
N	-0.69248700	0.41656200	1.94752900
C	-0.52470200	2.86663500	1.78484800
C	-1.05159500	3.03443400	0.50414200
C	-1.15888000	4.29355500	-0.19048900
C	-1.69020700	4.02737400	-1.42024000
C	-1.91218100	2.60400500	-1.48501100
C	-2.41063600	1.92028800	-2.59372300
H	-2.67137200	2.51860600	-3.46126300
N	-1.52493000	2.01847900	-0.29836000
H	-1.90554200	4.72317700	-2.22100100
H	-0.85385900	5.25014600	0.21398700
H	-0.20228200	3.76350100	2.30470700
H	0.53932200	2.32393200	4.39904900
H	0.50420400	-0.31723100	4.98029300
H	-0.28442400	-2.48236300	3.67990000
H	-0.97838500	-4.69289700	2.40870800
H	-2.01760300	-5.21956600	-0.03220000
H	-2.73506600	-3.72647900	-2.09305500
H	-3.37403900	-2.29827800	-4.22940100
H	-3.34827100	0.34430600	-4.80630300
S	-4.13489800	0.37093400	1.19245000
H	-4.41608700	-0.94810100	1.28103000
C	3.31963200	-0.17265400	-1.98607200
C	4.55865400	-1.02454700	-1.65998600
C	5.64707200	-0.18008800	-0.90665000
C	4.79054300	-0.49022500	0.38823200
C	4.31639100	-1.79148400	-0.32951000
H	3.29773900	-2.12775800	-0.12462800
H	5.00894400	-2.62229200	-0.18191300

C	3.64280100	0.48308300	0.46735300
C	2.66958300	0.42859700	-0.71432200
C	3.46266400	1.34564900	1.47393100
H	2.64812500	2.06737400	1.47153000
H	4.12235100	1.35998600	2.33893800
H	5.33030700	-0.56961700	1.33874400
C	7.01181600	-0.88586900	-0.87368800
H	7.67891800	-0.39772700	-0.15196900
H	7.49109000	-0.82851200	-1.85948100
H	6.94491500	-1.94308900	-0.60090600
C	5.87237800	1.28134400	-1.30392800
H	6.31678900	1.34180400	-2.30584400
H	6.56964200	1.76156900	-0.60532200
H	4.95652200	1.87830300	-1.30728500
H	4.89084600	-1.59130800	-2.53811300
H	2.55854900	-0.78665700	-2.48111200
H	3.58465200	0.62761100	-2.68625000
O	1.54750000	-0.42112800	-0.38923000
H	2.29401100	1.44008800	-0.92273000
H	1.14588800	-0.08905000	0.43297900

Table S14: Coordinates Beta-Pinene

Alpha site – Doublet state

Coordinate 1

Fe	1.45571400	0.01158500	0.17518400
N	1.90242500	1.91962000	-0.30792600
C	2.75612700	2.34091300	-1.29634500
C	2.73440400	3.77923900	-1.40491400
C	1.84654300	4.22696200	-0.47475400
C	1.33157600	3.06016600	0.19916600
C	0.39212600	3.09372700	1.22481200
C	-0.10145900	1.98604700	1.90657100
C	-1.05138700	2.04949900	2.99103400
C	-1.26799700	0.76671200	3.39406800
C	-0.45087000	-0.07377100	2.55229200
N	0.25742000	0.68600800	1.65587200
C	-0.39276700	-1.46019200	2.64503400
C	0.39010400	-2.29568400	1.85447500
C	0.43390100	-3.73172000	1.98321900
C	1.30211500	-4.18133900	1.03535700
C	1.78432000	-3.01725400	0.33307700
N	1.22224700	-1.87680500	0.84759900
C	2.69794000	-3.05294800	-0.71595300
C	3.19388500	-1.94457700	-1.39512400
C	4.16326200	-2.00693700	-2.46231000
C	4.39922100	-0.72281900	-2.84831600
C	3.57231900	0.11633900	-2.01490600
C	3.53049800	1.50462200	-2.09387400
H	4.15975600	1.97716900	-2.84144500

N	2.84644700	-0.64428000	-1.13493100
H	5.06684200	-0.35847500	-3.61797600
H	4.59661200	-2.92062300	-2.84722000
H	3.06155700	-4.02800000	-1.02448400
H	1.60164000	-5.19836600	0.81916600
H	-0.13041000	-4.30171900	2.70947300
H	-1.01010900	-1.93191400	3.40296600
H	-1.91094500	0.40498600	4.18563500
H	-1.47870800	2.96343400	3.38195600
H	0.02170200	4.06834000	1.52647100
H	1.55649200	5.24489700	-0.25011000
H	3.32688400	4.35193900	-2.10617300
O	0.23481000	-0.09143900	-0.91030400
S	3.35180400	0.16476500	1.87892800
H	3.20019800	1.46428500	2.21678600
C	-6.33194400	-0.74861600	-0.80700900
C	-5.55464300	0.30665800	-0.00092100
C	-4.37526800	-0.38387500	0.74022300
C	-3.57367500	-0.22365800	-0.58920200
C	-4.48745900	1.03143000	-0.89566800
C	-4.82553500	1.35056800	-2.35440800
H	-5.60334300	2.12360100	-2.40853400
H	-3.93956300	1.73942200	-2.87314800
H	-5.17537100	0.48186900	-2.91788400
C	-3.94527800	2.30904800	-0.23531700
H	-4.69882000	3.10648100	-0.27450400
H	-3.66657500	2.16891400	0.81331800
H	-3.05546900	2.66774600	-0.76861700
C	-3.93247500	-1.34957400	-1.52723800
C	-5.37608300	-1.82809200	-1.39574900
H	-5.74681300	-2.20262800	-2.35628400

H	-5.37698300	-2.69307000	-0.71900200
C	-3.05989900	-1.88618400	-2.38795500
H	-3.34609200	-2.67866800	-3.07700000
H	-2.02513000	-1.55223600	-2.42617200
H	-2.49214500	-0.05595600	-0.54253100
H	-4.53673100	-1.40056700	1.11447500
H	-3.96131500	0.22096900	1.54924000
H	-6.23205000	0.94617800	0.57853900
H	-6.90112000	-0.25800700	-1.60544800
H	-7.06926200	-1.23588700	-0.15719100

Coordinate II – TS (-1655.11)

Fe	-1.08984500	0.02570200	0.34743800
N	-0.64993500	-1.81826200	1.01892300
C	-1.12433600	-3.00239600	0.50720900
C	-0.56675600	-4.11945600	1.23267200
C	0.24515300	-3.59772800	2.19419000
C	0.18664800	-2.16290400	2.04879000
C	0.88279700	-1.25425600	2.84193400
C	0.84574400	0.12852100	2.69931700
C	1.56915400	1.05817000	3.53349000
C	1.27267900	2.30549200	3.07540400
C	0.36203000	2.13619100	1.96750600
N	0.12051300	0.80101400	1.74405500
C	-0.20599400	3.18694500	1.25503700
C	-1.14585600	3.06916100	0.23369900
C	-1.74312000	4.18556800	-0.46195900
C	-2.61620100	3.65828100	-1.36471100
C	-2.54352600	2.22270300	-1.21993600
N	-1.64640600	1.88653400	-0.23909200
C	-3.27500400	1.31607100	-1.98211600

C	-3.19720100	-0.07024000	-1.88706300
C	-3.93910500	-0.99604900	-2.71328300
C	-3.57795700	-2.24701700	-2.31555300
C	-2.62155600	-2.07960000	-1.24388900
C	-2.02444500	-3.12632700	-0.54604100
H	-2.29791800	-4.13492100	-0.84088900
N	-2.40339200	-0.75025100	-1.00099500
H	-3.91788200	-3.20170800	-2.69570900
H	-4.63641000	-0.70807600	-3.48936400
H	-3.95174600	1.72615200	-2.72548000
H	-3.25295000	4.17518500	-2.07096800
H	-1.51401700	5.22585600	-0.27023100
H	0.09239500	4.19178600	1.53781600
H	1.61984600	3.26144000	3.44565400
H	2.21181500	0.77531100	4.35708100
H	1.50413300	-1.65770400	3.63534900
H	0.83467500	-4.11887400	2.93725200
H	-0.78310000	-5.15894800	1.02256300
O	0.07385700	-0.03434300	-0.96990600
S	-2.96511200	0.03854100	1.91399000
H	-2.26354200	-0.14671900	3.05461800
C	5.31383900	0.21360500	-1.03746000
C	4.39691900	-0.97984400	-0.71731900
C	3.38211000	-0.56697300	0.39439500
C	2.57131200	0.00152500	-0.78136600
C	3.18550300	-1.08571800	-1.73060200
C	3.39802100	-0.73385900	-3.20177900
H	4.02630400	-1.49285100	-3.68450900
H	2.43592200	-0.71866300	-3.72878800
H	3.86694200	0.24127800	-3.35075100
C	2.49414200	-2.45455700	-1.64244800

H	3.11889500	-3.21725800	-2.12335900
H	2.30517900	-2.77885600	-0.61558500
H	1.53229200	-2.42723600	-2.16620900
C	3.03525600	1.37368100	-1.16347800
C	4.53727900	1.55542600	-0.92141200
H	4.93846200	2.31219200	-1.60366200
H	4.67371800	1.95332400	0.09228300
C	2.25314000	2.32166100	-1.69179500
H	2.65966500	3.28311100	-1.99757200
H	1.18650900	2.16490100	-1.81661000
H	3.71307900	0.13164300	1.16613400
H	2.91289600	-1.42845800	0.87078200
H	4.95527300	-1.91125700	-0.55972500
H	5.73997000	0.09532900	-2.04000200
H	6.15762600	0.21991700	-0.33769400
H	1.26939900	-0.03576200	-0.72924100

Coordinate III

Fe	-1.23600300	0.04435300	0.30908100
N	-0.65529200	-1.74535500	1.05959000
C	-1.02106900	-2.97850600	0.57509600
C	-0.37642300	-4.02360000	1.33477700
C	0.38255400	-3.41115800	2.28454500
C	0.21110600	-1.98993200	2.09863100
C	0.85003400	-1.00587700	2.84303000
C	0.72778000	0.36632300	2.63860300
C	1.41074700	1.37565900	3.41200200
C	1.02396600	2.57967200	2.90723500
C	0.10189400	2.30101500	1.83190000
N	-0.05950000	0.94860200	1.67778200
C	-0.55258600	3.27711700	1.08635000

C	-1.48791000	3.05286900	0.08146200
C	-2.16319500	4.09920200	-0.65223100
C	-3.00161100	3.47996600	-1.52713400
C	-2.82945000	2.05937600	-1.32603100
N	-1.91028100	1.82323000	-0.34420100
C	-3.50115300	1.07428600	-2.04714800
C	-3.32259700	-0.29487600	-1.90127700
C	-3.98948300	-1.30456700	-2.69341400
C	-3.52025700	-2.50592400	-2.26365100
C	-2.57602900	-2.22707300	-1.20300600
C	-1.89163300	-3.20145300	-0.48437300
H	-2.07638200	-4.23620800	-0.75830100
N	-2.47308800	-0.87854400	-0.99947000
H	-3.78053900	-3.49667900	-2.61395200
H	-4.71337600	-1.10128200	-3.47224700
H	-4.20902200	1.40701400	-2.80073200
H	-3.67573700	3.92554300	-2.24751000
H	-2.00595200	5.15986400	-0.50292900
H	-0.32179800	4.31208500	1.32187400
H	1.31898100	3.57113700	3.22660000
H	2.08914400	1.17345500	4.23118100
H	1.50638700	-1.33350300	3.64410400
H	1.00712600	-3.86422700	3.04407400
H	-0.50361000	-5.08358900	1.15502400
O	0.05148100	0.01060000	-0.95656500
S	-3.08625200	-0.21895300	1.86404700
H	-2.37212500	-0.36833200	3.00296700
C	5.67120100	0.25558100	-1.16935400
C	4.80096900	-0.91748300	-0.66956400
C	3.93597100	-0.42746000	0.53754900
C	3.02912700	0.07893500	-0.57953800

C	3.46285000	-1.07375400	-1.51652600
C	3.48060300	-0.81793000	-3.02159200
H	4.00077900	-1.63239200	-3.54166300
H	2.45245700	-0.78038700	-3.40005800
H	3.95911800	0.12520100	-3.29436600
C	2.76177100	-2.41589400	-1.24830000
H	3.27790400	-3.22323300	-1.78306100
H	2.72824000	-2.68534500	-0.18915100
H	1.72871100	-2.36823100	-1.60879200
C	3.39870000	1.41881800	-1.09053700
C	4.92072100	1.61478600	-1.02829100
H	5.24436000	2.33139300	-1.79108100
H	5.17873600	2.06100400	-0.05894700
C	2.54013800	2.30479600	-1.60703000
H	2.88514200	3.24986400	-2.02160900
H	1.47291600	2.10376700	-1.62028600
H	4.36709600	0.30899900	1.22021600
H	3.51216800	-1.25144300	1.11450200
H	5.37895400	-1.84245500	-0.53127200
H	5.96283400	0.08073200	-2.21119900
H	6.60081800	0.28880900	-0.58828200
H	0.91765500	-0.05932900	-0.50244700

Coordinate V

Fe	-1.12882000	0.03680500	0.37771900
N	-1.94098600	-0.25740500	-1.44811800
C	-2.36510200	0.69135300	-2.35340800
C	-2.97964600	0.05679000	-3.49261400
C	-2.93908900	-1.28594300	-3.26374600
C	-2.29822200	-1.47391800	-1.98493300
C	-2.08506200	-2.71263300	-1.39400800

C	-1.47958100	-2.92414700	-0.16158000
C	-1.25680100	-4.22907500	0.41561600
C	-0.63512500	-4.03057800	1.61111000
C	-0.47882400	-2.60434000	1.76528900
N	-0.99617700	-1.94566800	0.67437500
C	0.10817900	-1.99151000	2.86484800
C	0.25640300	-0.61913200	3.01496600
C	0.85441000	0.01545400	4.16360100
C	0.79879200	1.35858400	3.94306900
C	0.16480700	1.54575800	2.65980800
N	-0.14842000	0.32805000	2.10158100
C	-0.08834200	2.78755400	2.09153900
C	-0.70044900	2.99981500	0.86306300
C	-0.94636300	4.30466900	0.29646200
C	-1.54807300	4.10380700	-0.90873000
C	-1.67075000	2.67626800	-1.07845200
C	-2.23770900	2.06452500	-2.18913800
H	-2.61403500	2.70448300	-2.98076000
N	-1.14573400	2.01793900	0.01043000
H	-1.88132700	4.83951400	-1.62955300
H	-0.68209400	5.24070600	0.77195200
H	0.21702800	3.66402600	2.65444800
H	1.14774200	2.16262300	4.57851200
H	1.25881200	-0.51345700	5.01720300
H	0.47243200	-2.62915800	3.66400800
H	-0.30428100	-4.76718400	2.33211800
H	-1.54159400	-5.16366800	-0.05077900
H	-2.41781300	-3.58875100	-1.94179100
H	-3.30468600	-2.08876600	-3.89135800
H	-3.38517200	0.58494300	-4.34614000
O	0.82827700	-0.11641700	-0.84216000

S	-3.16823700	0.14288900	1.37484000
H	-2.90794600	-0.65101200	2.43710900
C	5.05459500	0.17618600	-0.48952800
C	4.08836200	-1.00700900	-0.33154700
C	2.87535800	-0.58792500	0.55042900
C	2.25175600	-0.03454600	-0.75008600
C	3.09996100	-1.11318600	-1.54751300
C	3.59222500	-0.76100900	-2.95426100
H	4.31922400	-1.50690800	-3.29935700
H	2.75500900	-0.76826300	-3.66413100
H	4.06380600	0.22246500	-3.01721900
C	2.41076400	-2.48504600	-1.60304200
H	3.11658000	-3.23023300	-1.99022900
H	2.05996400	-2.83533800	-0.62901100
H	1.54764300	-2.46102100	-2.27823900
C	2.82199400	1.34382400	-1.03962100
C	4.26941500	1.51325500	-0.58216200
H	4.78580800	2.23472900	-1.22409400
H	4.24188800	1.96695600	0.41730400
C	2.13863100	2.32562900	-1.63493000
H	2.60948300	3.28067300	-1.85607000
H	1.09104600	2.23375500	-1.90369300
H	0.53699800	0.14715600	-1.73133400
H	3.04754200	0.11761600	1.36816700
H	2.31450200	-1.44155200	0.92983100
H	4.61181400	-1.92967900	-0.05880000
H	5.67960500	0.02784900	-1.37763200
H	5.73703000	0.21441700	0.36779800

Alpha site – Quartet state

Coordinate I

Fe	1.46165200	0.01802400	0.19463400
N	1.85111000	2.01056800	-0.23522400
C	2.67895600	2.46206700	-1.21949500
C	2.59124900	3.92593900	-1.28640800
C	1.70214600	4.31031900	-0.34060100
C	1.23086900	3.08849200	0.32255700
C	0.29686600	3.04296400	1.36629700
C	-0.17096500	1.89419700	2.01848400
C	-1.14367900	1.87914900	3.11852900
C	-1.31905500	0.58299300	3.46941400
C	-0.45733400	-0.21686100	2.58932000
N	0.21079200	0.61612400	1.74280100
C	-0.34423600	-1.61298100	2.62167000
C	0.46105800	-2.41011100	1.79727300
C	0.54414300	-3.87435600	1.86056700
C	1.41892100	-4.26028700	0.90213900
C	1.88668100	-3.03866000	0.23595000
N	1.28660400	-1.95883900	0.81084200
C	2.80864200	-2.99442800	-0.81844200
C	3.27624600	-1.84542700	-1.46971700
C	4.25449700	-1.82994000	-2.56482300
C	4.44366400	-0.53305800	-2.90327600
C	3.58388800	0.26685300	-2.02127500
C	3.48360800	1.66427500	-2.04364300
H	4.09025100	2.18320800	-2.77857000
N	2.90730600	-0.56622300	-1.18212200
H	5.09247600	-0.12459900	-3.66699100
H	4.71541900	-2.71117300	-2.99149100
H	3.20183700	-3.94509900	-1.16322300
H	1.73768000	-5.26230500	0.64641700

H	-0.00673400	-4.49291900	2.55689300
H	-0.94586800	-2.13212400	3.36055300
H	-1.95706600	0.17592400	4.24287600
H	-1.60707500	2.75992400	3.54340100
H	-0.10393900	3.99301100	1.70388800
H	1.37584500	5.31157200	-0.09148400
H	3.14794900	4.54518100	-1.97747600
O	0.22993000	-0.08721600	-0.90079300
S	3.33506500	0.14847000	1.84911000
H	3.13268400	1.41633200	2.27087000
C	-6.34563800	-0.71580200	-0.91454500
C	-5.58015200	0.31576700	-0.06741200
C	-4.44838700	-0.40639400	0.71676700
C	-3.58828000	-0.25394300	-0.57651400
C	-4.46066100	1.02339000	-0.91037800
C	-4.72973800	1.36107600	-2.37925100
H	-5.48926700	2.14985100	-2.45948000
H	-3.81497300	1.73648400	-2.85626400
H	-5.07199900	0.50405900	-2.96485200
C	-3.91927100	2.28369500	-0.21687400
H	-4.65077700	3.09941300	-0.28570900
H	-3.69413800	2.13080900	0.84280500
H	-2.99687300	2.62374800	-0.70507700
C	-3.93170500	-1.36450300	-1.53826000
C	-5.38965100	-1.81195000	-1.47137900
H	-5.72766100	-2.17127000	-2.44964000
H	-5.43803900	-2.68154000	-0.80229300
C	-3.03519500	-1.91389400	-2.36563900
H	-3.30906800	-2.69484300	-3.07265600
H	-1.99284600	-1.60216500	-2.35709900
H	-2.50636200	-0.11023800	-0.48405500

H	-4.64754300	-1.42197400	1.07549800
H	-4.05599500	0.18320300	1.54758000
H	-6.26725800	0.96574300	0.48850900
H	-6.86902300	-0.20633200	-1.73226900
H	-7.12068500	-1.19120000	-0.30085400

Coordinate II – TS (-1756.48)

Fe	-1.12266300	0.02568200	0.30660800
N	-2.19933700	-1.36748900	-0.67905200
C	-3.14005900	-1.16871000	-1.65483100
C	-3.64632500	-2.43281200	-2.13812800
C	-2.99194800	-3.40360400	-1.44335100
C	-2.09446700	-2.72741800	-0.53428900
C	-1.26015700	-3.37256800	0.37415500
C	-0.42272600	-2.75894400	1.30549100
C	0.36831300	-3.46988400	2.28243000
C	1.00476200	-2.52485000	3.03023900
C	0.60778700	-1.24348400	2.49730000
N	-0.26149800	-1.40602200	1.44939100
C	1.05917300	-0.01298900	2.97021500
C	0.70996900	1.23163000	2.45251000
C	1.20180300	2.49574200	2.94689000
C	0.62423400	3.46247100	2.18050300
C	-0.22256600	2.78264700	1.22801200
N	-0.15413400	1.42672800	1.40488400
C	-1.02379200	3.42858700	0.28613400
C	-1.90369300	2.81918600	-0.60361100
C	-2.75516900	3.53227500	-1.52832300
C	-3.47461700	2.59145200	-2.19980700
C	-3.05469100	1.30807300	-1.68584600
C	-3.54325900	0.07916500	-2.12460500

H	-4.29796300	0.09535300	-2.90489600
N	-2.10150900	1.46620700	-0.71532600
H	-4.21950700	2.73359000	-2.97188400
H	-2.78694800	4.60905000	-1.63246000
H	-0.97324500	4.51294300	0.26098100
H	0.74210400	4.53630400	2.24511600
H	1.89448800	2.60955100	3.77054300
H	1.74980300	-0.02645000	3.80774400
H	1.68154100	-2.66500300	3.86299100
H	0.41118800	-4.54716700	2.37604900
H	-1.28281200	-4.45816100	0.37651200
H	-3.09698700	-4.47794000	-1.52104100
H	-4.40012600	-2.54265700	-2.90686300
O	0.11834200	-0.04650700	-0.95358500
S	-2.94899400	0.10887200	1.96590500
H	-2.20179200	0.15793700	3.09061800
C	5.39913900	0.16058800	-1.01501200
C	4.46242200	-1.01881600	-0.69846100
C	3.45912200	-0.59408600	0.41606700
C	2.64222000	-0.00036800	-0.75105800
C	3.24615300	-1.09357400	-1.70449700
C	3.46555000	-0.74327300	-3.17616800
H	4.08211100	-1.51083600	-3.66113700
H	2.50424600	-0.71133000	-3.70437300
H	3.95006900	0.22453200	-3.32376000
C	2.53076900	-2.45166100	-1.62512000
H	3.14318200	-3.22349600	-2.10830300
H	2.33441100	-2.77723500	-0.60001700
H	1.56971000	-2.40714200	-2.14929000
C	3.14036000	1.36221000	-1.13152000
C	4.64545700	1.51503200	-0.89146300

H	5.06303800	2.26926500	-1.56702600
H	4.78982700	1.90276400	0.12542900
C	2.38495800	2.32935400	-1.66454100
H	2.81640100	3.28035200	-1.96999600
H	1.31594300	2.20033900	-1.80118700
H	3.81071900	0.09147900	1.19177500
H	2.97678500	-1.44862400	0.89074200
H	5.01175200	-1.95790200	-0.55074800
H	5.82161200	0.04048700	-2.01917800
H	6.24544100	0.15032400	-0.31783700
H	1.27443600	-0.02214700	-0.70128000

Coordinate III

Fe	-1.28387100	0.02142100	0.29029600
N	-1.18842500	-1.96223800	0.62654600
C	-1.81605600	-2.94739700	-0.09825700
C	-1.44325300	-4.24331000	0.40948400
C	-0.57506100	-4.03156300	1.43834700
C	-0.41116400	-2.60522100	1.56226600
C	0.43509700	-1.99366000	2.47617800
C	0.63671400	-0.62449900	2.57543700
C	1.48683000	0.00329400	3.55532300
C	1.38674700	1.34891500	3.36324800
C	0.47199700	1.54877300	2.26781100
N	0.04366200	0.33373500	1.78730100
C	0.05841100	2.79151200	1.81005300
C	-0.87500100	2.99409100	0.80429000
C	-1.27895900	4.29157300	0.32455700
C	-2.17525400	4.08188700	-0.67989800
C	-2.32134000	2.65504600	-0.82055100
N	-1.53638200	2.00952200	0.10547600

C	-3.11576700	2.04268600	-1.77927100
C	-3.22705300	0.67149100	-1.95260200
C	-4.06835700	0.04337600	-2.94045600
C	-3.93805100	-1.30250200	-2.77373900
C	-3.01857300	-1.50267300	-1.68168500
C	-2.66677700	-2.74472200	-1.17590200
H	-3.09155000	-3.62151200	-1.65301400
N	-2.58385900	-0.28737000	-1.20634400
H	-4.41778300	-2.10141200	-3.32431600
H	-4.67663700	0.58021900	-3.65690300
H	-3.68535800	2.68282100	-2.44451800
H	-2.69150600	4.81268700	-1.28893400
H	-0.90640400	5.23061800	0.71302700
H	0.48935000	3.66939400	2.27952100
H	1.87062600	2.14753600	3.91053700
H	2.07093500	-0.53264200	4.29222900
H	0.97907700	-2.63328300	3.16302400
H	-0.07380400	-4.76146700	2.06086100
H	-1.80364700	-5.18325700	0.01195300
O	0.05344200	-0.01216600	-0.90066800
H	0.93818700	0.02309900	-0.46747100
S	-3.03231900	-0.02665900	1.88483100
H	-3.29882500	1.29672700	1.92830000
C	5.78378000	0.18119500	-1.01242900
C	4.85907100	-0.97658800	-0.58609000
C	3.93237500	-0.49024100	0.57414100
C	3.09260200	0.05556400	-0.58331800
C	3.57666400	-1.08657900	-1.51710300
C	3.70708100	-0.80694000	-3.01325700
H	4.27262800	-1.61039000	-3.50145500
H	2.71377900	-0.77344200	-3.47799900

H	4.20213800	0.14126700	-3.23459800
C	2.84534500	-2.42521900	-1.32284900
H	3.41010100	-3.23379100	-1.80305200
H	2.70593900	-2.69319700	-0.27208300
H	1.85451400	-2.38232400	-1.78977400
C	3.53253600	1.39810100	-1.03990100
C	5.05483800	1.55305800	-0.90436200
H	5.43162200	2.26717000	-1.64423400
H	5.26876800	1.98875600	0.07995900
C	2.73076100	2.34066100	-1.54963300
H	3.12595700	3.28740600	-1.91136400
H	1.65579000	2.19567000	-1.61144700
H	4.33700500	0.22633900	1.29298500
H	3.46512300	-1.31565600	1.11298500
H	5.40523600	-1.91698500	-0.43102100
H	6.14188400	0.01137900	-2.03399400
H	6.67098800	0.18900800	-0.36871100

Coordinate V

Fe	1.35681500	-0.02094000	0.21469500
N	1.52175100	0.37172700	-1.81878900
C	1.72256200	-0.55209700	-2.81604300
C	1.95320400	0.12249500	-4.07004300
C	1.91988400	1.46232600	-3.81069800
C	1.66830200	1.60892100	-2.39779800
C	1.63923700	2.81409300	-1.70385900
C	1.43372300	2.95541600	-0.33516600
C	1.34093700	4.22771700	0.33935500
C	1.04425800	3.96977600	1.64314200
C	0.95314100	2.53630600	1.78251900
N	1.21517300	1.93723200	0.56880300

C	0.58265700	1.88948800	2.95735600
C	0.42722700	0.51546300	3.10795200
C	0.06337600	-0.15650400	4.33240400
C	0.09673200	-1.49579600	4.07283200
C	0.48112500	-1.64403200	2.68952500
N	0.65267800	-0.40796300	2.11806000
C	0.70273000	-2.85378000	2.04027500
C	1.09123100	-2.99901500	0.71234400
C	1.25704700	-4.27211400	0.05365400
C	1.55222700	-4.01322400	-1.25025700
C	1.57028100	-2.57860200	-1.40474500
C	1.75679000	-1.92913700	-2.62121100
H	1.93838400	-2.55318100	-3.49025500
N	1.30499200	-1.98008600	-0.19102400
H	1.72478900	-4.71613100	-2.05497300
H	1.13748300	-5.23143500	0.54031600
H	0.55243100	-3.76381600	2.61198800
H	-0.10731400	-2.31692900	4.74802700
H	-0.17390500	0.34223300	5.26338400
H	0.39109900	2.51363200	3.82415200
H	0.87726700	4.67333400	2.44847700
H	1.46751300	5.18663500	-0.14636400
H	1.78098800	3.72487700	-2.27656600
H	2.06280200	2.28387000	-4.50089400
H	2.12855500	-0.37491200	-5.01530300
O	-1.18524100	0.14962000	-0.59793700
S	3.70808900	-0.13210000	0.69809600
H	3.91162800	1.16854100	1.00444800
C	-5.30484000	-0.42788300	0.23707000
C	-4.35919300	0.75597600	0.48947500
C	-3.02320700	0.24378900	1.10405100

C	-2.57208100	-0.02077900	-0.35244900
C	-3.55744600	1.13703300	-0.80510000
C	-4.23526900	1.02678000	-2.17406500
H	-5.02138900	1.78608800	-2.27281400
H	-3.50662700	1.20572700	-2.97536900
H	-4.68758700	0.04921900	-2.35818500
C	-2.91283400	2.52710700	-0.69166900
H	-3.68155600	3.29862100	-0.82375200
H	-2.42783000	2.70360100	0.27204000
H	-2.15800700	2.67159900	-1.47345200
C	-3.13536400	-1.35567700	-0.81048100
C	-4.50870200	-1.68022200	-0.22470400
H	-5.08952100	-2.28055800	-0.93312900
H	-4.34360300	-2.32545100	0.64814000
C	-2.48891100	-2.18742400	-1.63289500
H	-2.94188100	-3.12057600	-1.95993400
H	-1.48541300	-1.98588500	-1.99619700
H	-1.01553800	0.12148800	-1.55437800
H	-3.06209700	-0.61163700	1.78549900
H	-2.43410500	1.03852300	1.56369500
H	-4.86575900	1.58084400	1.00235000
H	-6.05686500	-0.14668400	-0.50915400
H	-5.85372900	-0.66534200	1.15611300

Delta Site – Doublet state

Coordinate I (cis)

Fe	-1.46281500	0.00757500	0.13266300
N	-0.83911300	-1.16040200	1.65742000
C	-0.75057300	-2.52864000	1.67289400

C	-0.17121800	-2.98520900	2.91307400
C	0.09182800	-1.87184000	3.65224400
C	-0.32600500	-0.74190000	2.85805400
C	-0.22005600	0.58487800	3.26338200
C	-0.61518500	1.69462500	2.52411500
C	-0.48586800	3.06014100	2.97153700
C	-0.96150000	3.84609500	1.96673000
C	-1.38267300	2.95681200	0.91154900
N	-1.17080700	1.64944400	1.27020300
C	-1.93960100	3.37222700	-0.29392800
C	-2.38742900	2.53362900	-1.30967000
C	-2.99709500	2.98772000	-2.53612900
C	-3.30413300	1.87258700	-3.25427200
C	-2.87754900	0.74262700	-2.46476400
N	-2.32757100	1.16321900	-1.28008900
C	-3.00384800	-0.58473600	-2.86019000
C	-2.59914300	-1.69441100	-2.12474900
C	-2.74697100	-3.06057700	-2.56435100
C	-2.22724600	-3.84454800	-1.57975500
C	-1.76667600	-2.95285700	-0.54331400
C	-1.17586000	-3.36662100	0.64659200
H	-1.04634600	-4.43452500	0.79137300
N	-2.00425600	-1.64751300	-0.89001100
H	-2.15720600	-4.92355300	-1.53923200
H	-3.19277800	-3.35966800	-3.50379300
H	-3.45819900	-0.77154000	-3.82808400
H	-3.77427500	1.80124900	-4.22626600
H	-3.16275100	4.02562400	-2.79333000
H	-2.04744500	4.44119100	-0.44852100
H	-1.03207100	4.92507300	1.92665000
H	-0.08214600	3.35740200	3.93038600

H	0.20927400	0.77024400	4.24289000
H	0.52367200	-1.80246200	4.64196000
H	-0.00256000	-4.02304000	3.16858600
S	-3.77575300	-0.11545600	1.20173300
H	-4.07627100	1.20086900	1.14935000
O	0.01799800	0.08992100	-0.55949500
C	3.75567400	-0.67043700	-2.16271200
C	5.10037900	0.06516400	-2.03748100
C	5.10187100	1.04035300	-0.80719000
C	5.45523200	-0.24671600	0.04188900
C	6.10001900	-0.83061200	-1.25358300
H	6.03742700	-1.91356100	-1.40492000
H	7.14028400	-0.52273300	-1.37584800
C	4.19278600	-1.00961900	0.35716500
C	3.43044300	-1.48799600	-0.87697600
H	3.71030800	-2.53531800	-1.05318300
C	3.78622300	-1.26073500	1.60679700
H	2.86159200	-1.79561900	1.81565000
H	4.37238500	-0.94648400	2.46798500
H	6.08324400	-0.10310300	0.92861300
C	6.27329700	2.03422900	-0.85432600
H	6.38758500	2.53935900	0.11326700
H	6.08504500	2.80658500	-1.61128600
H	7.23020600	1.56218600	-1.09643900
C	3.82639600	1.81503200	-0.46388100
H	3.61976100	2.57167000	-1.23230300
H	3.94864200	2.34275900	0.49109100
H	2.94225300	1.17892700	-0.37385000
H	5.41983400	0.48261300	-3.00036500
H	3.79033900	-1.34972600	-3.02338300
H	2.95762300	0.05177500	-2.37152300

H	2.35292400	-1.49045400	-0.68165000

Coordinate II (cis) – TS (-1697.84)

Fe	-1.22461100	-0.00001900	0.22356500
N	-2.16691100	0.37210900	-1.53619000
C	-2.32467400	1.59953500	-2.12213700
C	-2.97629000	1.46815900	-3.40562000
C	-3.21008200	0.13939000	-3.58838800
C	-2.69411000	-0.53457700	-2.41904800
C	-2.71541000	-1.91296200	-2.22409800
C	-2.18903400	-2.58346800	-1.12307200
C	-2.18367600	-4.01894200	-0.96014700
C	-1.55768600	-4.27360500	0.22199000
C	-1.19016100	-2.99170100	0.77845800
N	-1.58749400	-1.97703000	-0.05078800
C	-0.53652400	-2.81803500	1.99635500
C	-0.19960400	-1.60112300	2.58365800
C	0.40043400	-1.46985100	3.89071200
C	0.53171500	-0.13531900	4.12813600
C	0.02097500	0.54262900	2.96074400
N	-0.41656000	-0.36485600	2.02830900
C	-0.01186700	1.92450800	2.79697200
C	-0.48048300	2.58903900	1.66620600
C	-0.48024800	4.02181300	1.49478600
C	-1.00275600	4.26550000	0.26056000
C	-1.33000800	2.98066600	-0.31208700
C	-1.92730700	2.80955700	-1.55819400
H	-2.12191200	3.70838400	-2.13541700
N	-0.99803300	1.97072600	0.55641700
H	-1.16691200	5.21706200	-0.22808500
H	-0.12282600	4.73104600	2.22982800

H	0.36693900	2.53293600	3.61226400
H	0.93345000	0.36004200	5.00241700
H	0.66974000	-2.30010200	4.53048300
H	-0.28896900	-3.71642500	2.55359900
H	-1.35748400	-5.22887100	0.68951300
H	-2.60328300	-4.72150100	-1.66832500
H	-3.16545600	-2.51796400	-3.00517200
H	-3.68087100	-0.35535500	-4.42792800
H	-3.21641800	2.29391300	-4.06260100
S	-3.41852800	0.14982300	1.31974200
H	-4.15079100	-0.51676000	0.40060400
O	0.25317500	-0.17851900	-0.68134300
C	3.17492300	-0.62188000	0.81470900
C	4.51988900	-0.89423600	0.11824700
C	4.39155900	-0.84229800	-1.44620000
C	4.50232300	0.72035400	-1.26830100
C	5.31254400	0.43754200	0.03808800
H	5.18488400	1.14738400	0.86291300
H	6.37938400	0.30046700	-0.14764600
C	3.19250300	1.34373700	-0.84144300
C	2.47779400	0.63367700	0.25662000
H	2.10334000	1.31261400	1.02753200
C	2.73760100	2.49610200	-1.37187100
H	1.81674700	2.96097200	-1.02702500
H	3.27987800	3.00810200	-2.16277700
H	4.97969600	1.29024400	-2.07298600
C	5.63577400	-1.41834600	-2.14397800
H	5.62378400	-1.16907000	-3.21258000
H	5.64254000	-2.51250100	-2.05898000
H	6.57828100	-1.04969900	-1.72887800
C	3.15337200	-1.43227400	-2.12711100

H	3.16893400	-2.52810600	-2.05514600
H	3.15482100	-1.17802600	-3.19546100
H	2.20815800	-1.08481100	-1.70556400
H	5.01559500	-1.77126900	0.55177900
H	3.34814900	-0.49407500	1.89155400
H	2.51581100	-1.49438100	0.71631000
H	1.34980800	0.25905200	-0.20353900

Coordinate III (cis)

Fe	-1.44376200	0.04864600	0.25151800
N	-0.03826300	0.19745500	1.69678800
C	0.51316900	-0.80563100	2.45369300
C	1.47889200	-0.26827700	3.38223500
C	1.50635100	1.07880700	3.17694900
C	0.55083400	1.35719000	2.12995600
C	0.25066700	2.63555400	1.66008200
C	-0.70866700	2.96134700	0.70343800
C	-1.00578500	4.30823100	0.27075300
C	-1.99491900	4.20820700	-0.65978400
C	-2.29227400	2.80046500	-0.79372300
N	-1.50516000	2.06004400	0.04758000
C	-3.23604200	2.26785800	-1.67010900
C	-3.51455300	0.91272700	-1.83465700
C	-4.47614700	0.37694600	-2.77102400
C	-4.42969000	-0.97764900	-2.64090100
C	-3.44385700	-1.26069700	-1.62176400
N	-2.90251100	-0.09803500	-1.14188800
C	-3.10673800	-2.54104800	-1.18636700
C	-2.19055500	-2.86064400	-0.18568800
C	-1.92866900	-4.20303400	0.27893200
C	-1.01634100	-4.09701100	1.28496800

C	-0.71786200	-2.69135100	1.42264100
C	0.18768900	-2.15568900	2.33667700
H	0.69278300	-2.84917700	3.00194600
N	-1.43960300	-1.95442000	0.51809200
H	-0.57543300	-4.88345400	1.88347800
H	-2.39516400	-5.09499200	-0.11869100
H	-3.62414400	-3.37102800	-1.65813300
H	-5.00041700	-1.72780500	-3.17268600
H	-5.09138400	0.97219600	-3.43316100
H	-3.79277300	2.96782700	-2.28563400
H	-2.48412200	4.99831700	-1.21443400
H	-0.51371300	5.19792200	0.64168800
H	0.80422100	3.46024800	2.09880800
H	2.10155600	1.82714500	3.68386000
H	2.04904500	-0.85598300	4.08981000
S	-3.15391400	0.20780100	1.96136900
H	-3.98314400	1.07817000	1.34390300
O	-0.19374600	-0.03921100	-1.07240500
H	0.52653500	-0.61510000	-0.75807300
C	5.23545100	-0.29682400	-2.70762400
C	5.72214800	0.23441700	-1.34654700
C	4.52777100	0.81539300	-0.50334100
C	4.24476200	-0.69202600	-0.12452200
C	5.76618900	-0.95394100	-0.34583300
H	6.04433800	-1.93517900	-0.74532500
H	6.36787200	-0.74813900	0.54113700
C	3.49848400	-1.39184400	-1.24879100
C	4.03740900	-1.18669600	-2.52960100
H	3.58429100	-1.65717600	-3.39950000
C	2.38947600	-2.18194400	-1.01865100
H	1.89357500	-2.70497800	-1.83216000

H	2.02019300	-2.35991300	-0.01310800
H	3.80527900	-0.89799200	0.85691700
C	5.01472200	1.62589400	0.70891600
H	4.18313300	1.81264500	1.39992200
H	5.40143000	2.59967100	0.38260700
H	5.80848900	1.12838700	1.27355000
C	3.43857400	1.62579200	-1.21146500
H	3.84563000	2.58253000	-1.56343100
H	2.61964400	1.85220000	-0.51676800
H	3.00519200	1.10880400	-2.07160000
H	6.61451600	0.86096400	-1.45643400
H	6.04594100	-0.85471700	-3.20391000
H	4.99287600	0.53023400	-3.39379000

Coordinate V (cis)

Fe	1.31044600	0.06450600	0.27348000
N	1.31633200	2.02810600	-0.19860600
C	1.78428200	2.62529000	-1.34765900
C	1.67701200	4.06002600	-1.24418800
C	1.15009300	4.32719600	-0.01654400
C	0.93361600	3.05580200	0.63197000
C	0.41597700	2.91177200	1.91322300
C	0.20016100	1.70403800	2.56608100
C	-0.35315600	1.59103900	3.89497300
C	-0.42236500	0.26090400	4.18246500
C	0.09033200	-0.44028900	3.03070800
N	0.45983300	0.45507600	2.05319700
C	0.19628600	-1.82216300	2.93220800
C	0.69930000	-2.49701900	1.82673800
C	0.82484300	-3.93101900	1.73004000
C	1.36954400	-4.19550300	0.50976800

C	1.57736100	-2.92329000	-0.14098000
N	1.15603800	-1.89978000	0.67420800
C	2.12361600	-2.77846000	-1.41070000
C	2.33689400	-1.57155500	-2.06565800
C	2.90240900	-1.45818100	-3.39003500
C	2.95099000	-0.12949800	-3.68550300
C	2.41509600	0.56990400	-2.54223500
C	2.29665200	1.95102300	-2.44876000
H	2.63106300	2.54486000	-3.29336000
N	2.04288600	-0.32523800	-1.56723800
H	3.30933300	0.34994200	-4.58759600
H	3.21245300	-2.29753800	-3.99956200
H	2.40664900	-3.68540500	-1.93583100
H	1.61351200	-5.15575400	0.07328100
H	0.52905200	-4.62814100	2.50365000
H	-0.13159200	-2.41532600	3.77984700
H	-0.78428400	-0.21777200	5.08351100
H	-0.64776700	2.43110100	4.51112000
H	0.15659400	3.81926100	2.44952100
H	0.92207000	5.28894100	0.42523900
H	1.97127300	4.75626400	-2.01919500
S	3.41313000	0.18200300	1.13728400
H	3.20531800	-0.53187100	2.26583200
C	-2.71913000	0.74673500	0.43518600
C	-4.23467300	0.48160700	0.37900200
C	-4.73752100	0.34190100	-1.10165900
C	-4.21980600	-1.15066100	-0.99096800
C	-4.49014600	-1.04453300	0.54231400
H	-3.83708600	-1.60570700	1.21857400
H	-5.52537900	-1.27744800	0.79523700
C	-2.73144900	-1.18715300	-1.24359300

C	-1.92417600	-0.43260600	-0.18567700
H	-1.67532500	-1.13777900	0.61100600
C	-2.16442800	-1.81180200	-2.27979600
H	-1.09123400	-1.78837800	-2.44337600
H	-2.76489400	-2.36307900	-2.99938300
H	-4.75109400	-1.91167800	-1.57239000
C	-6.27021800	0.41348100	-1.20203300
H	-6.60462000	0.07440600	-2.19034100
H	-6.60771300	1.44994200	-1.07498100
H	-6.78385500	-0.19412800	-0.45161700
C	-4.14889500	1.24635200	-2.18711800
H	-4.43340400	2.29155500	-2.01096900
H	-4.53893700	0.95957700	-3.17188900
H	-3.05888600	1.20168100	-2.25177100
H	-4.78181200	1.17527800	1.02769800
H	-2.39435400	0.88419500	1.47128300
H	-2.47629100	1.67665200	-0.09431200
O	-0.62884500	-0.03284700	-0.67390500
H	-0.71634000	0.69274300	-1.31536500

Coordinate I (trans)

Fe	1.45284200	0.05421500	0.17468700
N	2.64324500	0.13839700	-1.45499200
C	3.18701700	-0.92294700	-2.13402400
C	3.89734000	-0.46490300	-3.30359000
C	3.77022800	0.89010500	-3.33114800
C	2.98401100	1.25427900	-2.17735200
C	2.63470700	2.55780500	-1.84100300
C	1.90802800	2.93678700	-0.71657000
C	1.58859500	4.30081700	-0.37157600
C	0.88222500	4.25257300	0.79149500

C	0.77078500	2.85940700	1.14949500
N	1.40766900	2.07188400	0.22362700
C	0.10005700	2.38822400	2.27321500
C	-0.03165600	1.05269100	2.64134900
C	-0.73374100	0.59671100	3.81649500
C	-0.64013500	-0.76179100	3.82304000
C	0.12099800	-1.12752900	2.65272000
N	0.48418500	-0.01036700	1.94561100
C	0.45363000	-2.43379100	2.30546400
C	1.20953000	-2.80928400	1.19953400
C	1.56412000	-4.17007600	0.87700400
C	2.30458500	-4.11876300	-0.26450300
C	2.39761800	-2.72712100	-0.63367900
C	3.07100200	-2.25609300	-1.75646400
H	3.54996400	-2.99473000	-2.39147900
N	1.72952900	-1.94300300	0.27194100
H	2.75514800	-4.93284500	-0.81697500
H	1.27841800	-5.03504400	1.46092600
H	0.10310000	-3.22658800	2.95864600
H	-1.03649100	-1.46317300	4.54522000
H	-1.22464600	1.24476600	4.53051900
H	-0.36098300	3.12803900	2.91987700
H	0.46740000	5.07010000	1.36642000
H	1.87826200	5.16653800	-0.95264100
H	2.96832200	3.34827900	-2.50556600
H	4.16700900	1.59168700	-4.05293500
H	4.41875200	-1.10976700	-3.99858700
S	3.61269400	0.21488900	1.52618900
H	4.14418600	1.28607000	0.89656600
O	0.07910100	-0.06101400	-0.70896700
C	-3.97807900	-0.76262500	0.84782700

C	-4.56547100	0.57274600	0.35815200
C	-3.61517800	1.18755900	-0.70893000
C	-4.34866100	0.27787300	-1.74335500
C	-5.61070700	0.34599600	-0.79161300
C	-3.75591100	-1.10904300	-1.69588400
C	-3.26442300	-1.52020200	-0.31073700
H	-3.34131800	-2.60584200	-0.18143000
C	-3.65886400	-1.89926200	-2.77178500
H	-3.25636300	-2.90860500	-2.70869400
H	-3.97557200	-1.56061100	-3.75603100
H	-4.47778700	0.63775400	-2.77061500
H	-4.88304200	1.20325100	1.19792700
H	-4.77271500	-1.37933800	1.28476900
H	-3.25644000	-0.57274200	1.65225300
H	-2.19315200	-1.28357000	-0.26586500
C	-6.54374200	-0.86584000	-0.72792800
H	-7.25810300	-0.75756800	0.09876000
H	-7.12513100	-0.94697600	-1.65557000
H	-6.01457500	-1.81251300	-0.59368700
C	-6.46273000	1.59733200	-1.05760800
H	-7.20427100	1.72759000	-0.25875300
H	-5.87342300	2.51748100	-1.11286400
H	-7.00960100	1.49599400	-2.00379200
H	-2.53834600	1.03581400	-0.58114800
H	-3.80187800	2.24864000	-0.88595900

Coordinate II (trans) – TS (-1661.71)

Fe	-1.27880700	0.00789600	0.20876200
N	-0.72563000	1.67336100	1.19205200
C	-0.15826500	1.77370300	2.43799100
C	0.09007200	3.15542100	2.77230000

C	-0.33186500	3.89426600	1.70837100
C	-0.84534800	2.96089300	0.73332800
C	-1.40861800	3.33356200	-0.48492100
C	-1.98724600	2.47919100	-1.42018900
C	-2.59874600	2.91090000	-2.65645200
C	-3.05520500	1.78819900	-3.27691500
C	-2.71384500	0.67593400	-2.42031400
N	-2.07211700	1.11816100	-1.29283400
C	-2.98502500	-0.65924400	-2.70873800
C	-2.62977300	-1.74726600	-1.91612300
C	-2.88350400	-3.12894000	-2.25363600
C	-2.35897100	-3.87793400	-1.24473400
C	-1.79362200	-2.94924800	-0.29253100
N	-1.97350100	-1.65958500	-0.71545200
C	-1.16567700	-3.31996600	0.89467400
C	-0.64263200	-2.45693800	1.85412800
C	-0.07529100	-2.88814500	3.11026400
C	0.27764400	-1.76043800	3.78711600
C	-0.06571000	-0.64584100	2.93722400
C	0.14524500	0.69211200	3.25974200
H	0.59118800	0.90847800	4.22549700
N	-0.62209000	-1.08728700	1.76345800
H	0.72852100	-1.67255300	4.76692600
H	0.02250700	-3.92059700	3.41957600
H	-1.09919600	-4.38339100	1.10366700
H	-2.34830100	-4.95471000	-1.13677400
H	-3.39216700	-3.46139600	-3.14913100
H	-3.50214200	-0.87101200	-3.63958200
H	-3.57119700	1.70135400	-4.22419100
H	-2.66280900	3.93957300	-2.98649900
H	-1.42012200	4.39467100	-0.71505800

H	-0.31411200	4.96891200	1.58207200
H	0.52910400	3.49576600	3.70101600
S	-3.45914100	0.17152100	1.33862800
H	-4.25646600	-0.09025400	0.27981100
O	0.17868600	-0.13614100	-0.72949700
C	3.06985900	-1.29417000	0.23158200
C	3.96283600	-1.46287000	-1.00974400
C	3.31093600	-0.70484400	-2.20054700
C	4.01607900	0.57056000	-1.64512300
C	5.11268100	-0.39374400	-1.03607000
C	3.17971900	1.14662900	-0.52633000
C	2.49286600	0.13058800	0.31019300
H	2.26426300	0.47083600	1.32362900
C	3.06342700	2.47239500	-0.30751700
H	2.47147700	2.86641800	0.51534100
H	3.56256000	3.19737200	-0.94579300
H	4.35427900	1.34075000	-2.34687300
H	4.25361500	-2.51052100	-1.15229600
H	3.63930800	-1.51749100	1.14423800
H	2.25059400	-2.02379800	0.19841300
H	1.32065200	0.04147600	-0.16778700
C	5.79470700	0.00508500	0.27546800
H	6.39040400	-0.83027400	0.66578100
H	6.47933800	0.84652200	0.10770800
H	5.09155600	0.30807300	1.05563900
C	6.20367600	-0.73928600	-2.06179600
H	6.82492600	-1.56424000	-1.69005900
H	5.80272400	-1.04079200	-3.03392600
H	6.86186500	0.12315500	-2.22660100
H	2.21789200	-0.68996800	-2.24791000
H	3.70642300	-1.00876800	-3.17148100

Coordinate III (trans)

Fe	-1.42204900	-0.01498900	0.23093400
N	-2.64649800	-0.63187200	-1.25349300
C	-3.29808300	0.17498000	-2.14794600
C	-4.06156900	-0.62133800	-3.08310200
C	-3.85914900	-1.92376200	-2.74083400
C	-2.97147100	-1.91609900	-1.59990300
C	-2.50752100	-3.05776600	-0.94914900
C	-1.64456900	-3.06969400	0.14320900
C	-1.15873300	-4.26428500	0.79603600
C	-0.33660400	-3.85048600	1.79967400
C	-0.32683400	-2.40476300	1.75635400
N	-1.13015500	-1.95159100	0.74599500
C	0.39601200	-1.59766400	2.63569400
C	0.42023400	-0.20387000	2.64571700
C	1.13961800	0.59257700	3.61314300
C	0.90443300	1.89824200	3.29836200
C	0.04523500	1.89380800	2.13755000
N	-0.23551700	0.60636100	1.75406100
C	-0.42836800	3.03401900	1.49247000
C	-1.26654600	3.04175100	0.37819600
C	-1.73929500	4.23417100	-0.28564700
C	-2.52575500	3.81805100	-1.31718700
C	-2.53276100	2.37362700	-1.27646000
C	-3.24257700	1.56722600	-2.16323400
H	-3.81382000	2.07287700	-2.93599600
N	-1.75928000	1.92173600	-0.23810000
H	-3.05940100	4.41678300	-2.04401200
H	-1.49217300	5.24531500	0.01089300
H	-0.12118500	3.99592000	1.89121000

H	1.26844800	2.78869500	3.79441900
H	1.73692500	0.19058400	4.42133100
H	0.97688000	-2.10354400	3.40106400
H	0.21713100	-4.45014000	2.51055400
H	-1.42072000	-5.27464600	0.50967000
H	-2.84203500	-4.01795400	-1.32989600
H	-4.26095100	-2.81536500	-3.20467800
H	-4.66477500	-0.21933500	-3.88694300
S	-3.30404200	0.05917100	1.74539600
H	-3.91505200	-1.08832600	1.37523500
O	0.00046400	-0.09842200	-0.92159400
C	3.65681500	-0.75767800	0.67179900
C	4.26153300	-1.36017800	-0.60980900
C	3.30969200	-1.02866500	-1.79326100
C	4.04163000	0.34770100	-1.86452800
C	5.30816600	-0.38017800	-1.25871300
C	3.41393900	1.25102300	-0.81809400
C	3.22028100	0.65146900	0.44250800
H	2.79431800	1.22111300	1.26428000
C	3.06469100	2.56123000	-1.05291900
H	2.62061000	3.17229000	-0.27209200
H	3.22131500	3.02554700	-2.02184000
H	4.14993400	0.84791200	-2.83194200
H	4.56924600	-2.40004200	-0.45567300
H	4.36263700	-0.79101400	1.51720400
H	2.79041400	-1.35457800	1.01047100
H	0.57737400	0.65192100	-0.70146200
C	6.26095800	0.40723200	-0.35451500
H	6.98785100	-0.26980300	0.11179700
H	6.82521900	1.14078500	-0.94434700
H	5.75338300	0.95247900	0.44597300

C	6.14418100	-1.06844300	-2.34946200
H	6.87903700	-1.74266500	-1.89196800
H	5.54449700	-1.65853700	-3.04804000
H	6.69686400	-0.32214000	-2.93355700
H	2.23657100	-1.00318800	-1.58003600
H	3.49033800	-1.64646400	-2.67441400

Coordinate V (trans)

Fe	-1.29276100	0.06239000	0.26233100
N	-0.54993800	1.06684400	1.83823100
C	-0.10644100	0.55537600	3.03601000
C	0.27011800	1.62542900	3.92791800
C	0.04082800	2.79084500	3.26119600
C	-0.47581000	2.43368000	1.96089300
C	-0.83457800	3.35485900	0.98415400
C	-1.33403600	3.04140300	-0.27411800
C	-1.68554600	4.02299700	-1.27363800
C	-2.12376700	3.33528300	-2.36444900
C	-2.04058600	1.93296500	-2.03278600
N	-1.55533600	1.77367000	-0.75603000
C	-2.40819300	0.90119700	-2.88754500
C	-2.33741500	-0.44959700	-2.57244500
C	-2.74906600	-1.51833900	-3.44785900
C	-2.54658100	-2.68345500	-2.77025100
C	-2.00981600	-2.32969500	-1.47889000
N	-1.87883400	-0.96191600	-1.37807900
C	-1.67241200	-3.24795700	-0.49229100
C	-1.15919100	-2.93333600	0.76084700
C	-0.81890400	-3.91422200	1.76438700
C	-0.35797600	-3.22787300	2.84721700
C	-0.41550300	-1.82742700	2.50676400
C	-0.03901700	-0.79460000	3.35624700

H	0.32868900	-1.06128600	4.34192500
N	-0.89919800	-1.66610300	1.22811900
H	-0.00684000	-3.61686500	3.79449300
H	-0.92472500	-4.98423900	1.63819700
H	-1.82332700	-4.29881200	-0.71831700
H	-2.73639000	-3.69709300	-3.09978900
H	-3.13973800	-1.37845900	-4.44770600
H	-2.78540900	1.17036200	-3.86895100
H	-2.47132000	3.72319500	-3.31351300
H	-1.59814200	5.09384000	-1.14050900
H	-0.71375200	4.40669400	1.22361300
H	0.20160100	3.80616300	3.60089200
H	0.65663800	1.48432000	4.92921000
S	-3.39979200	0.14708800	1.12170700
H	-4.05941600	0.44851800	-0.01870200
C	2.66287400	0.90798700	0.36872100
C	3.80295800	1.26814300	-0.59826500
C	4.85431700	0.10685000	-0.69976200
C	3.84647500	-0.52830500	-1.74165700
C	3.36740400	0.92410400	-2.04944900
H	2.31287300	1.06148500	-2.30412000
H	3.98180400	1.40985900	-2.80949700
C	2.75784500	-1.26172300	-1.00397700
C	1.94789800	-0.40624600	-0.02943400
C	2.47976600	-2.56118000	-1.16647100
H	1.70713000	-3.06024900	-0.58525200
H	3.01821900	-3.17039300	-1.88851300
H	4.27193600	-1.13361700	-2.54929900
C	6.15987700	0.56048400	-1.37119000
H	6.77420200	-0.30769000	-1.64082900
H	6.74487600	1.17909600	-0.67871600

H	5.99711100	1.14706000	-2.28005000
C	5.21171400	-0.69473700	0.55494800
H	5.76671500	-0.06763000	1.26437200
H	5.85560500	-1.54374700	0.29228400
H	4.34121300	-1.09960000	1.07866300
H	4.19006400	2.27293600	-0.39241000
H	1.91451900	1.70629800	0.38139800
H	3.04540200	0.81984000	1.39174100
O	0.68101400	-0.04487100	-0.68062100
H	1.68706000	-0.99064800	0.85584100
H	0.48346000	-0.72082400	-1.35405200

Delta Site – Quartet state

Coordinate I (cis)

Fe	-1.46470400	0.00608200	0.13290700
N	-1.25763400	1.70588900	1.20316500
C	-0.72061700	1.83791300	2.45812600
C	-0.67310900	3.22692800	2.84552700
C	-1.18681200	3.93858400	1.80450700
C	-1.54546100	2.98034100	0.78715300
C	-2.10649800	3.31273500	-0.44221000
C	-2.47400900	2.41388300	-1.43787600
C	-3.05965300	2.79126500	-2.70157700
C	-3.26446200	1.63828200	-3.39591000
C	-2.80453800	0.56155900	-2.55254600
N	-2.33102700	1.05118300	-1.36150700
C	-2.84941600	-0.78529700	-2.89836100
C	-2.43902400	-1.84198300	-2.09152300
C	-2.52114000	-3.23376200	-2.46278200
C	-2.02706700	-3.94644500	-1.41320600

C	-1.64378300	-2.98690500	-0.40618100
N	-1.90869400	-1.70960800	-0.83282300
C	-1.07750100	-3.31867600	0.82015100
C	-0.69960900	-2.41868700	1.81188500
C	-0.12585800	-2.79775400	3.08054400
C	0.09757900	-1.64273800	3.76692600
C	-0.34026800	-0.56495500	2.91290400
C	-0.28778300	0.78282300	3.25535700
H	0.11951600	1.03218300	4.23009000
N	-0.82448500	-1.05579700	1.72790700
H	0.51281600	-1.51355300	4.75768300
H	0.06799200	-3.81677600	3.38855900
H	-0.91950900	-4.37330400	1.02274600
H	-1.92400700	-5.01872900	-1.31145700
H	-2.91024400	-3.59719500	-3.40475700
H	-3.24936400	-1.03394100	-3.87632800
H	-3.68761100	1.50867100	-4.38326500
H	-3.27820600	3.80852700	-2.99871100
H	-2.27589200	4.36650900	-0.63991300
H	-1.32034500	5.00867700	1.71600200
H	-0.29620700	3.58895900	3.79299900
O	0.03121100	0.11091000	-0.52498100
S	-3.77511900	-0.14948600	1.15539800
H	-4.44635700	-0.53732800	0.04870000
C	3.76688500	-0.86435100	-2.01836600
C	5.09199800	-0.08375700	-2.00304900
C	5.06792200	1.05538400	-0.92309500
C	5.45415000	-0.08914800	0.09828700
C	6.11443900	-0.83354200	-1.10378900
H	6.08019000	-1.92824600	-1.10028600
H	7.14633300	-0.51923100	-1.27184500

C	4.21244200	-0.83288000	0.52249700
C	3.46367600	-1.50091500	-0.62910300
H	3.77224900	-2.55460900	-0.65684800
H	2.38659500	-1.50545200	-0.43156600
C	3.81345600	-0.91702400	1.79672200
H	2.90423900	-1.44262100	2.08264700
H	4.39122800	-0.46910700	2.60267100
H	6.07787500	0.19455100	0.95373600
C	6.21324700	2.06240700	-1.11401000
H	6.31334000	2.70243800	-0.22813000
H	6.00560800	2.71452400	-1.97226900
H	7.18246600	1.58599400	-1.28939100
C	3.77274300	1.83808800	-0.68912600
H	3.54748600	2.47264600	-1.55649800
H	3.88105800	2.49910000	0.18081600
H	2.90480900	1.19926100	-0.50723900
H	5.40068700	0.20127200	-3.01643200
H	3.81918700	-1.65690700	-2.77501500
H	2.94989800	-0.19984000	-2.32323800

Coordinate II (cis) – TS (-1858.04)

Fe	-1.22974600	-0.00575100	0.20468000
N	-2.17660100	0.44487700	-1.51507300
C	-2.32926000	1.69751800	-2.05524500
C	-3.01308600	1.61971600	-3.32583700
C	-3.27276500	0.30224100	-3.54891100
C	-2.74098000	-0.41999000	-2.41653700
C	-2.78107900	-1.80419900	-2.26811000
C	-2.25804800	-2.51929200	-1.19349300
C	-2.29040500	-3.95778000	-1.06812300
C	-1.65942600	-4.26013900	0.09973900

C	-1.24974300	-3.00494300	0.68615000
N	-1.62718100	-1.95581000	-0.11483800
C	-0.58111500	-2.88552500	1.90060200
C	-0.21071800	-1.69417500	2.52468500
C	0.40592900	-1.61468300	3.82835400
C	0.55907600	-0.28951700	4.10524200
C	0.04487800	0.42997300	2.96396100
N	-0.41624800	-0.44176000	2.01187100
C	0.03149100	1.81795900	2.84133400
C	-0.42893000	2.52971600	1.73529200
C	-0.39407300	3.96741900	1.61103200
C	-0.91863600	4.26351600	0.38863400
C	-1.28060400	3.00457800	-0.21990200
C	-1.90003300	2.88140500	-1.46376400
H	-2.08722000	3.80025300	-2.01115100
N	-0.96902200	1.96200700	0.61040300
H	-1.06312500	5.23380200	-0.06825000
H	-0.01446600	4.64357300	2.36586500
H	0.42817400	2.39376200	3.67184000
H	0.97803600	0.17279500	4.98947100
H	0.67015600	-2.46802300	4.43915100
H	-0.34947600	-3.80717700	2.42580200
H	-1.48197400	-5.23251800	0.54036800
H	-2.73801400	-4.62959300	-1.78881700
H	-3.25387500	-2.37521400	-3.06117300
H	-3.77220800	-0.15527400	-4.39292400
H	-3.25602200	2.47122800	-3.94793000
S	-3.39840400	0.15722000	1.36634400
H	-4.16591700	-0.45973600	0.44055000
O	0.28730800	-0.14412900	-0.65417700
C	3.21659000	-0.65596000	0.80597400

C	4.53802200	-0.93935100	0.06927000
C	4.37326300	-0.84744700	-1.49012200
C	4.51900200	0.70781700	-1.27801500
C	5.35602000	0.37773900	0.00022900
H	5.26303600	1.07009000	0.84432900
H	6.41506000	0.22366300	-0.21507100
C	3.23433400	1.34846600	-0.80063900
C	2.53251800	0.62567100	0.29323200
H	2.18058100	1.29065500	1.08575800
C	2.79719500	2.52672100	-1.29028300
H	1.89971200	3.00802800	-0.90802500
H	3.33197900	3.04400800	-2.08292100
H	4.98788800	1.28729700	-2.08101000
C	5.58939000	-1.43020600	-2.23086100
H	5.55747800	-1.15481200	-3.29268000
H	5.57654500	-2.52610800	-2.17247400
H	6.54857100	-1.09010600	-1.82982100
C	3.10884900	-1.39803700	-2.15519400
H	3.10354600	-2.49535400	-2.10588800
H	3.09284300	-1.12226100	-3.21821500
H	2.18026200	-1.04013000	-1.70629900
H	5.02650500	-1.83633500	0.46923200
H	3.42169300	-0.55789300	1.88049600
H	2.53766300	-1.51283800	0.70435500
H	1.36939300	0.27076600	-0.16303200

Coordinate III (cis)

Fe	1.48645100	0.04025400	0.23873400
N	2.68771000	-1.26796800	-0.70686200
C	2.70030300	-2.63620900	-0.55706700
C	3.65847700	-3.21854700	-1.46161800

C	4.21058300	-2.19335800	-2.16963500
C	3.59254700	-0.97936200	-1.70120700
C	3.85825800	0.28160400	-2.21546100
C	3.24242400	1.45045100	-1.79585600
C	3.55092100	2.76008300	-2.31224900
C	2.78443400	3.64958100	-1.62126700
C	2.00413000	2.88757700	-0.67922800
N	2.28269800	1.54744400	-0.81486900
C	1.13306800	3.44463100	0.24409000
C	0.40677200	2.72161900	1.17915300
C	-0.52020100	3.30690800	2.11380900
C	-1.06646900	2.28115500	2.82599900
C	-0.47697100	1.06410600	2.32869600
N	0.43468100	1.35562000	1.33888700
C	-0.81749500	-0.20424500	2.77533600
C	-0.28760900	-1.38627800	2.27893200
C	-0.60576300	-2.69721600	2.78395600
C	0.15733600	-3.58676700	2.08803600
C	0.94568700	-2.82399200	1.15471100
C	1.89675100	-3.36847500	0.30459000
H	2.02472500	-4.44558900	0.31772500
N	0.64046600	-1.48630400	1.26746700
H	0.20290500	-4.66320200	2.19087900
H	-1.31651700	-2.89185600	3.57658100
H	-1.55056900	-0.27640600	3.57151600
H	-1.80834300	2.32430800	3.61283900
H	-0.72048000	4.36746000	2.19397400
H	1.01147300	4.52246900	0.23595700
H	2.74341400	4.72658600	-1.72009100
H	4.27038800	2.95431500	-3.09715200
H	4.60116300	0.35789400	-3.00215700

H	4.96024900	-2.23694500	-2.94903100
H	3.86081900	-4.27894900	-1.53872400
S	3.22371500	0.13624100	1.81719700
H	2.66900500	1.02406600	2.67054800
O	0.18657800	-0.10568700	-1.00449100
H	-0.40924600	-0.82286300	-0.71970600
C	-3.26387700	1.19504900	-1.62028700
C	-4.16603600	0.17793100	-2.34485800
C	-4.38927100	-1.11249900	-1.47321800
C	-5.48364700	-0.25080000	-0.72944600
C	-5.64829200	0.54806000	-2.05825400
H	-5.88832500	1.61273800	-1.96554000
H	-6.35105300	0.08424200	-2.75321300
C	-4.83378900	0.67743900	0.28632500
C	-3.74130900	1.41086100	-0.20787800
H	-3.21428600	2.11469400	0.43312100
C	-5.30555100	0.78239300	1.57923100
H	-4.84314900	1.45806700	2.29442600
H	-6.15427800	0.19835500	1.92275300
H	-6.35077800	-0.77644500	-0.31534200
C	-5.01451600	-2.25892700	-2.28487900
H	-5.36268300	-3.05571100	-1.61533000
H	-4.26844000	-2.69666600	-2.96050000
H	-5.86626500	-1.94394400	-2.89453000
C	-3.21836300	-1.68510600	-0.66892900
H	-2.46735500	-2.11364200	-1.34663000
H	-3.56325500	-2.49530000	-0.01351700
H	-2.72864700	-0.93914300	-0.03728000
H	-3.85423900	0.03342800	-3.38600600
H	-3.26644700	2.14837700	-2.17388600
H	-2.21366800	0.86116100	-1.61953100

Coordinate V

Fe	-1.69311300	0.05888800	0.39213000
N	-0.21677700	-0.55247500	1.63078300
C	0.05827400	-1.85265800	2.01293600
C	1.03268100	-1.86832500	3.06987300
C	1.33768900	-0.56859400	3.34772500
C	0.55129900	0.24441400	2.45969600
C	0.56025700	1.63027000	2.45611200
C	-0.20676900	2.41938000	1.61247100
C	-0.15454600	3.85563700	1.58621000
C	-1.01564800	4.26593200	0.61169200
C	-1.59618100	3.08120000	0.04115800
N	-1.10177800	1.95372300	0.66824000
C	-2.50077900	3.08764800	-1.00934600
C	-3.03598400	1.94935400	-1.59090300
C	-3.97094100	1.96370900	-2.68500000
C	-4.28166500	0.66492300	-2.95584200
C	-3.53641100	-0.14453900	-2.02811500
N	-2.76812400	0.65240100	-1.20388000
C	-3.60260200	-1.52776900	-1.97576900
C	-2.88114900	-2.31669000	-1.09296400
C	-2.92312100	-3.75355800	-1.07627400
C	-2.05734900	-4.16398800	-0.10590200
C	-1.48486200	-2.97849400	0.47177500
C	-0.53005700	-2.98740400	1.47745600
H	-0.21756200	-3.95005000	1.86775500
N	-2.00320700	-1.85070100	-0.13405100
H	-1.81048500	-5.17359200	0.19582700
H	-3.53350800	-4.35666600	-1.73574800
H	-4.25421600	-2.02935800	-2.68332400

H	-4.95126900	0.27334900	-3.71057500
H	-4.33201200	2.86077700	-3.17101900
H	-2.80125100	4.04965500	-1.41059100
H	-1.24014400	5.27451400	0.28976300
H	0.47386900	4.45782100	2.22939100
H	1.21843000	2.13283300	3.15677900
H	2.02759100	-0.17969300	4.08528700
H	1.42067000	-2.76648400	3.53246600
S	-3.54260400	0.06566400	2.04851100
H	-3.49070700	1.38926400	2.31720300
C	3.12433500	1.15058700	-1.48698900
C	4.66330800	1.21510600	-1.45465900
C	5.24664100	0.32329900	-0.30056300
C	5.05613600	-0.87614000	-1.31746300
C	5.23916600	0.17353100	-2.45826800
H	4.70176500	0.01024300	-3.39766600
H	6.29043000	0.34718700	-2.69248200
C	3.61986100	-1.34380200	-1.28290000
C	2.62811400	-0.29247500	-1.78487100
H	2.57584000	-0.40161100	-2.87468000
C	3.23524100	-2.55173900	-0.85948400
H	2.18676900	-2.83436400	-0.82884700
H	3.95901500	-3.29349800	-0.52983900
H	5.76214600	-1.71087500	-1.24598600
C	6.73161800	0.61934600	-0.03164500
H	7.17036000	-0.16701800	0.59522100
H	6.83462100	1.56938400	0.50807900
H	7.33333500	0.69141100	-0.94204700
C	4.52501700	0.26677800	1.04730300
H	4.57954200	1.24007900	1.55192800
H	5.00393500	-0.47155900	1.70315000

H	3.47196500	-0.01129100	0.96911900
H	5.02314900	2.24908700	-1.51299800
H	2.72949800	1.82381300	-2.25728800
H	2.70607100	1.48968700	-0.53089200
O	1.28242800	-0.51423500	-1.37044600
H	1.24509600	-0.51114800	-0.39832200

Coordinate I (trans)

Fe	1.50148200	-0.00305600	0.12354000
N	1.52142100	-1.96813400	0.58106100
C	1.02598600	-2.55437800	1.71881500
C	1.13854600	-3.99054700	1.63803700
C	1.70154100	-4.27008700	0.43036700
C	1.93323500	-3.00320900	-0.21988600
C	2.50829200	-2.85645400	-1.47833300
C	2.77119800	-1.64599300	-2.11141300
C	3.40142400	-1.51890000	-3.40308200
C	3.48582000	-0.18567400	-3.66547700
C	2.90487300	0.49573300	-2.53393200
N	2.48093300	-0.40911200	-1.59321200
C	2.78794300	1.87718200	-2.42359100
C	2.23472200	2.56310200	-1.34654900
C	2.13619700	4.00002000	-1.25975000
C	1.54130900	4.27778300	-0.06679300
C	1.28020300	3.00905300	0.56859800
N	1.71073900	1.97585600	-0.22376600
C	0.68625800	2.86138000	1.81822300
C	0.44643800	1.65144000	2.46237300
C	-0.14513800	1.52736000	3.77251700
C	-0.19806700	0.19512100	4.05039800

C	0.36029900	-0.48647700	2.90779900
C	0.48431600	-1.86836000	2.80062000
H	0.12828600	-2.46331400	3.63580100
N	0.75038000	0.41572100	1.95185700
H	-0.57249500	-0.29711900	4.93826800
H	-0.46528300	2.36023400	4.38451900
H	0.39392100	3.76748600	2.33940300
H	1.29801400	5.24157500	0.36073500
H	2.48376500	4.68766000	-2.01938600
H	3.16386600	2.47287400	-3.24946500
H	3.89848400	0.30854800	-4.53514600
H	3.73111100	-2.35118800	-4.01086500
H	2.78750900	-3.76305500	-2.00574500
H	1.94729000	-5.23398000	0.00454400
H	0.82332800	-4.67667100	2.41302200
S	3.78701500	0.12455200	1.20665200
H	4.30560200	-1.00989800	0.68719500
O	0.02635200	-0.07796000	-0.58453100
C	-4.00819500	1.45258900	-1.54116700
C	-4.67359400	0.16208100	-2.04934800
C	-3.81944000	-1.06062800	-1.60874300
C	-4.58712200	-0.98303000	-0.25237700
C	-5.78448200	-0.33235800	-1.05620900
C	-3.94137800	0.06038000	0.62563900
C	-3.35006000	1.23663200	-0.14638200
H	-3.39258200	2.15122900	0.45605200
C	-3.88051100	-0.04338000	1.95861800
H	-3.43414900	0.73420700	2.57544400
H	-4.27173000	-0.91496900	2.47941100
H	-4.79711200	-1.91026800	0.29304800
H	-4.95680400	0.24578800	-3.10587500

H	-4.74955400	2.25954800	-1.50181500
H	-3.23976700	1.77384400	-2.25542600
H	-2.28421800	1.02338900	-0.29933900
H	-4.05269900	-1.96965900	-2.16647300
H	-2.73191900	-0.93620000	-1.57915600
C	-6.66651700	0.69768500	-0.34539500
H	-7.32657400	1.19986900	-1.06479300
H	-7.30619100	0.20239300	0.39668200
H	-6.09544500	1.46682600	0.18070500
C	-6.69678400	-1.39601000	-1.68744800
H	-7.30254500	-1.88914100	-0.91653100
H	-7.38646400	-0.92814800	-2.40192100
H	-6.14541900	-2.17420400	-2.22347700

Coordinate II (trans) – TS (-1823.33)

Fe	-1.28190100	-0.00908700	0.19898600
N	-2.16931200	1.02573700	-1.28449900
C	-2.16472500	2.38949800	-1.44042700
C	-2.85579600	2.76130100	-2.65367600
C	-3.27877100	1.60381600	-3.23195900
C	-2.83956700	0.52936800	-2.37236500
C	-3.05575200	-0.82402800	-2.62033000
C	-2.62970500	-1.87424300	-1.81081400
C	-2.85077800	-3.27348100	-2.09278000
C	-2.26657600	-3.97057100	-1.07979500
C	-1.69549300	-2.99408200	-0.18091900
N	-1.93061200	-1.72276200	-0.64144500
C	-1.01498600	-3.30785100	0.99166000
C	-0.48791600	-2.39831900	1.90865400
C	0.14368800	-2.76959400	3.15335700
C	0.47443700	-1.60747900	3.78269600

C	0.05141200	-0.53532500	2.91345000
N	-0.52929400	-1.03616100	1.77724400
C	0.21695800	0.82065900	3.18816700
C	-0.15670700	1.86912100	2.34986500
C	0.05670600	3.26752100	2.63821900
C	-0.43395100	3.96175800	1.57312100
C	-0.95285200	2.98222200	0.64718400
C	-1.58822600	3.29467000	-0.55465400
H	-1.66159600	4.34722400	-0.81109600
N	-0.76911800	1.71572200	1.13303600
H	-0.45985600	5.03239400	1.41763700
H	0.52071600	3.64867100	3.53851000
H	0.69038500	1.08234200	4.12950600
H	0.95838000	-1.47118000	4.74094300
H	0.29667200	-3.78723300	3.48819500
H	-0.90505200	-4.36189900	1.22785600
H	-2.21912300	-5.04209900	-0.93568000
H	-3.38220700	-3.65198700	-2.95615000
H	-3.59850700	-1.08255300	-3.52433600
H	-3.83354600	1.47040300	-4.15144700
H	-2.99152700	3.77804500	-2.99851800
S	-3.41326300	0.09544900	1.43754600
H	-4.24984000	-0.20232900	0.41861500
O	0.20195300	-0.09436300	-0.71857200
C	3.12057900	-1.11815900	0.35781500
C	3.98135400	-1.42950400	-0.87868000
C	3.28505000	-0.83276400	-2.13427900
C	3.98570900	0.51003900	-1.76155900
C	5.11368800	-0.35848700	-1.07121500
C	3.17152500	1.21027900	-0.69772400
C	2.52645600	0.29859600	0.27535300

H	2.31483000	0.75921700	1.24317600
C	3.03864200	2.55249900	-0.64502900
H	2.46450600	3.03868500	0.14063900
H	3.50343300	3.19796300	-1.38628100
H	4.29248300	1.19107900	-2.56304200
H	4.28433900	-2.48332200	-0.89930700
H	3.71848700	-1.22281900	1.27378300
H	2.31160400	-1.85567100	0.43739000
H	1.32547400	0.13440800	-0.17375600
H	3.65731300	-1.25034400	-3.07174000
H	2.19094300	-0.83637000	-2.14878300
C	5.82594900	0.20740800	0.16035000
H	6.44355700	-0.56672600	0.63409100
H	6.49426100	1.02837700	-0.13014100
H	5.13970600	0.59782800	0.91629800
C	6.18062900	-0.81641600	-2.07800600
H	6.82079000	0.02626200	-2.36846400
H	6.82477300	-1.58131100	-1.62544800
H	5.75709100	-1.24151500	-2.99267800

Coordinate III (trans)

Fe	-1.45890100	0.02709200	0.15179600
N	-1.67982300	1.93069700	-0.45795800
C	-1.26014800	3.06406000	0.20157700
C	-1.53681700	4.22880800	-0.60033900
C	-2.10705200	3.78830100	-1.75659100
C	-2.18354800	2.35227700	-1.66627300
C	-2.67283700	1.53248000	-2.67275300
C	-2.72638000	0.14868600	-2.60589000
C	-3.27433400	-0.69349200	-3.63944000
C	-3.19832000	-1.97624800	-3.18689500

C	-2.60231300	-1.92375700	-1.87551000
N	-2.30545000	-0.62122900	-1.54742000
C	-2.39554300	-3.03116400	-1.06769400
C	-1.84696000	-2.98866600	0.20627300
C	-1.59867500	-4.15016300	1.02176300
C	-1.00984400	-3.71060800	2.16942900
C	-0.89679100	-2.27841200	2.06127500
N	-1.42790800	-1.85632700	0.86398600
C	-0.30973000	-1.46782000	3.02189500
C	-0.17016700	-0.09146600	2.91803300
C	0.38056200	0.74974100	3.94965600
C	0.28422000	2.03575800	3.50691600
C	-0.32232000	1.98538500	2.20115400
C	-0.63474600	3.10154300	1.43874000
H	-0.37077200	4.07450000	1.83915800
N	-0.57000600	0.67865700	1.84954200
H	0.58125000	2.94716700	4.00942100
H	0.77383600	0.38662700	4.89033200
H	0.06025400	-1.94415600	3.92347400
H	-0.66828600	-4.28862500	3.01831000
H	-1.84056800	-5.16445000	0.73173500
H	-2.68115100	-4.00132500	-1.46002100
H	-3.51706200	-2.88641800	-3.67827900
H	-3.66877500	-0.33027500	-4.57961900
H	-3.04218100	2.00836400	-3.57480800
H	-2.44279400	4.36615700	-2.60788700
H	-1.30605600	5.24391800	-0.30412500
S	-3.55716600	0.15054700	1.21660700
H	-3.86520000	1.42762000	0.90312300
O	0.15255200	-0.13704200	-0.62672000
C	3.53926400	-1.66816700	-0.07947400

C	4.28570300	-1.32858300	-1.38361500
C	3.54654000	-0.15042600	-2.07751800
C	4.36686100	0.78363400	-1.13638500
C	5.47893300	-0.33844000	-1.11794300
C	3.64897700	0.83788000	0.20365900
C	3.21762300	-0.41097200	0.68786700
H	2.71147500	-0.48558800	1.64880400
C	3.45104100	2.02855800	0.87511300
H	2.93465800	2.05743800	1.83100600
H	3.80453300	2.97176800	0.46926800
H	4.65209000	1.77911200	-1.49293500
H	4.50050900	-2.23032400	-1.96892300
H	4.13456700	-2.35239300	0.54590900
H	2.61377100	-2.21721600	-0.31523500
H	0.85450300	0.08872000	0.01445300
H	3.81410900	-0.03579300	-3.12969100
H	2.45678700	-0.11678600	-1.97764100
C	6.33654000	-0.51915700	0.13783800
H	6.93575400	-1.43572300	0.06102300
H	7.03531800	0.32060500	0.24597200
H	5.74754500	-0.57867200	1.05648100
C	6.42135900	-0.23079400	-2.32796400
H	7.10338400	0.62030400	-2.20629900
H	7.03477100	-1.13702300	-2.41231900
H	5.89509500	-0.09957700	-3.27781400

Coordinate V (trans)

Fe	1.47893300	-0.04784500	0.22564300
N	0.87630600	-1.60018500	1.35522800
C	0.44222300	-1.55333200	2.66232500
C	0.22090600	-2.88559900	3.16268900

C	0.53318200	-3.74426400	2.15146200
C	0.94730800	-2.93771100	1.03224900
C	1.37240300	-3.45003000	-0.18467200
C	1.78030500	-2.68045500	-1.26472300
C	2.18952200	-3.22157000	-2.53331700
C	2.47943000	-2.16174900	-3.34110500
C	2.24653500	-0.97075400	-2.56835700
N	1.83012100	-1.30213400	-1.29534300
C	2.39796300	0.31739300	-3.06192300
C	2.13591700	1.47439300	-2.34356900
C	2.30693300	2.80729100	-2.85919700
C	1.98024100	3.66652600	-1.85222300
C	1.60980100	2.86060300	-0.71871500
N	1.69376300	1.52049200	-1.03725900
C	1.24124600	3.36999000	0.51771600
C	0.90081300	2.59832600	1.62040400
C	0.49293500	3.14003000	2.88906600
C	0.21766600	2.08063000	3.70292000
C	0.45538900	0.88918600	2.93321800
C	0.25265200	-0.39903400	3.40753500
H	-0.08939300	-0.51018200	4.43109400
N	0.88087300	1.21893600	1.66210700
H	-0.12459600	2.08966400	4.72965500
H	0.42361800	4.19708800	3.11092500
H	1.21448400	4.44859600	0.63112100
H	1.98717000	4.74883500	-1.85953200
H	2.63715300	3.03911800	-3.86354600
H	2.73825900	0.42620900	-4.08631700
H	2.81094500	-2.16919100	-4.37138700
H	2.23461900	-4.27809300	-2.76381500
H	1.38161500	-4.52842200	-0.30284600

H	0.49434200	-4.82591500	2.14748900
H	-0.12767100	-3.11609800	4.16111600
S	3.85294000	-0.03812300	1.05586200
H	4.28988600	-1.03556000	0.25536200
C	-2.80352200	-1.06486100	0.26505700
C	-3.99687500	-1.33326200	-0.66747000
C	-5.11445700	-0.24453600	-0.49020500
C	-4.23048500	0.62430100	-1.47500600
C	-3.70190400	-0.71983500	-2.06485800
H	-2.66593300	-0.73845400	-2.41187600
H	-4.35108000	-1.11063800	-2.85052800
C	-3.12804300	1.29175700	-0.69597500
C	-2.18291400	0.33477300	0.03358200
C	-2.94977900	2.61634900	-0.62707700
H	-2.16498600	3.05799300	-0.01538300
H	-3.58232200	3.30671600	-1.18047200
H	-4.75417700	1.32769200	-2.13188100
C	-6.44429300	-0.66495000	-1.13511700
H	-7.12469300	0.19303900	-1.20547100
H	-6.93690800	-1.42956000	-0.52085600
H	-6.32491800	-1.07709600	-2.14133100
C	-5.41103500	0.30696100	0.90713400
H	-5.87174000	-0.46702100	1.53444700
H	-6.11914700	1.14307600	0.84294000
H	-4.52337200	0.67489900	1.42887900
H	-4.31256900	-2.38189100	-0.61253100
H	-2.01619100	-1.80726300	0.09514600
H	-3.11083400	-1.16578700	1.31206300
H	-1.87378600	0.77819700	0.98730400
O	-0.98841900	0.13980900	-0.77221300
H	-0.73753500	1.00632100	-1.13679300

Delta Site – Sextet state

Coordinate I (cis)

Fe	-1.41563300	0.01315200	0.11997500
N	-0.80922400	-0.82611200	1.92806100
C	-0.64446700	-2.15569800	2.19524600
C	-0.11328300	-2.31794100	3.53301900
C	0.03425500	-1.06440400	4.05329900
C	-0.40653700	-0.13359500	3.03513600
C	-0.42558700	1.26435700	3.15844600
C	-0.85700200	2.21022900	2.21655200
C	-0.86758900	3.64673900	2.40326800
C	-1.35992000	4.19151800	1.25288100
C	-1.65224600	3.08963500	0.35922700
N	-1.33869000	1.90961700	0.97311500
C	-2.18930400	3.19760500	-0.93267300
C	-2.51638000	2.15880700	-1.81794700
C	-3.09484900	2.31823000	-3.13593800
C	-3.25915000	1.06431500	-3.64947500
C	-2.78116500	0.13448300	-2.64723600
N	-2.34407200	0.82867200	-1.55374500
C	-2.76350800	-1.26332300	-2.76925300
C	-2.31947300	-2.20798500	-1.83146200
C	-2.34692000	-3.64621700	-2.00309400
C	-1.83845000	-4.18990300	-0.85921500
C	-1.49887600	-3.08496200	0.01393900
C	-0.95971800	-3.19396500	1.30494900
H	-0.77391500	-4.20277300	1.66221800
N	-1.80124600	-1.90512000	-0.60405300
H	-1.70554200	-5.23626000	-0.61671900

H	-2.71276900	-4.15920000	-2.88295300
H	-3.14734200	-1.66397700	-3.70322300
H	-3.66503600	0.78318100	-4.61249700
H	-3.34036100	3.26702500	-3.59496700
H	-2.38634100	4.20571600	-1.28581200
H	-1.51786800	5.23686100	1.02131100
H	-0.54175000	4.15809000	3.29966700
H	-0.06864500	1.66296200	4.10388400
H	0.40154600	-0.78472800	5.03209300
H	0.10821100	-3.26777000	4.00202900
O	0.09868300	0.09294500	-0.51778700
S	-3.71437500	-0.11392600	1.10714500
H	-4.17680000	1.06867700	0.64645200
C	3.77994500	-0.63085400	-2.19214700
C	5.11503500	0.10602700	-1.99355600
C	5.06412400	1.04508700	-0.73651300
C	5.39073800	-0.26485700	0.08804500
C	6.08789300	-0.80847500	-1.19779500
H	6.03617200	-1.88677000	-1.38274300
H	7.13067600	-0.49359100	-1.27058700
C	4.12091900	-1.04148800	0.33196500
C	3.40834900	-1.48602500	-0.94390200
H	3.69858400	-2.52714100	-1.13916100
H	2.32417500	-1.49541200	-0.79021300
C	3.66845600	-1.33144100	1.55719600
H	2.73902300	-1.87567700	1.71383700
H	4.21953100	-1.04110700	2.44934800
H	5.98346700	-0.14479900	1.00215600
C	6.23165500	2.04428200	-0.70971300
H	6.30664200	2.52095400	0.27599000
H	6.06833700	2.83809300	-1.45012100

H	7.19945500	1.58345000	-0.92919500
C	3.77235900	1.80466100	-0.42113300
H	3.59023500	2.57956300	-1.17755900
H	3.85699400	2.30916000	0.55041900
H	2.88887100	1.16258100	-0.37924600
H	5.46941700	0.55255500	-2.93087200
H	3.85153100	-1.28497800	-3.06988700
H	2.98728800	0.09416700	-2.41134300

Coordinate (cis) – TS (-1896.64)

Fe	1.21189900	0.02333000	0.20323800
N	0.33919400	0.21406400	2.08228300
C	0.06834500	1.38781200	2.72532800
C	-0.54323200	1.10618600	4.00860400
C	-0.61902200	-0.25286600	4.11978600
C	-0.05733300	-0.80387200	2.90301400
C	0.07203000	-2.17344900	2.60840000
C	0.62097200	-2.76565800	1.45883800
C	0.73271700	-4.18778400	1.19715500
C	1.33431000	-4.31671000	-0.02226000
C	1.59422000	-2.97287500	-0.50379400
N	1.14686000	-2.06766700	0.41094400
C	2.23184900	-2.63242600	-1.71047000
C	2.54578500	-1.35379000	-2.19949100
C	3.22295700	-1.07238300	-3.45056400
C	3.32811500	0.28463700	-3.54772500
C	2.71495600	0.83962900	-2.35687900
N	2.25770500	-0.17627200	-1.56569200
C	2.60402000	2.20743800	-2.05265600
C	2.03030800	2.79900500	-0.91494800

C	1.94652400	4.22044100	-0.63715100
C	1.31681100	4.34971200	0.56701200
C	1.01468700	3.00765700	1.02844400
C	0.36822100	2.66948100	2.22740300
H	0.07583700	3.50212000	2.86150400
N	1.46250100	2.10112900	0.11041600
H	1.07565800	5.26031700	1.10017000
H	2.32275900	5.00386300	-1.28235700
H	3.01725400	2.89155200	-2.78870100
H	3.77657500	0.86568600	-4.34315400
H	3.56769900	-1.82163300	-4.15150900
H	2.53245900	-3.46506100	-2.34072400
H	1.58685900	-5.22771600	-0.54937700
H	0.39487600	-4.97218200	1.86210700
H	-0.29651700	-2.85774500	3.36771700
H	-1.01122200	-0.83600900	4.94294000
H	-0.86029600	1.85432100	4.72363200
S	3.34257800	-0.04851900	1.40041100
H	4.11277800	0.54492800	0.46176600
O	-0.29207700	0.09659100	-0.69120900
C	-3.26118400	0.42753000	0.83886800
C	-4.58583300	0.74413400	0.12144700
C	-4.40320000	0.83155400	-1.43602600
C	-4.49467700	-0.74204000	-1.39922800
C	-5.35500200	-0.58478600	-0.10347000
H	-5.24505000	-1.36291800	0.65999100
H	-6.41673800	-0.44444200	-0.31444400
C	-3.19309900	-1.38688200	-0.97688800
C	-2.52733000	-0.76289800	0.19474400
H	-2.14544700	-1.49482900	0.91108900
C	-2.70868200	-2.48968300	-1.58516600

H	-1.79745600	-2.97673100	-1.24512700
H	-3.21630800	-2.93649200	-2.43624000
H	-4.93456400	-1.24490400	-2.26751500
C	-5.63252600	1.44895500	-2.12521800
H	-5.57788400	1.29648500	-3.21059800
H	-5.66255900	2.53094000	-1.94384900
H	-6.58235900	1.03033200	-1.78011000
C	-3.15357300	1.49822800	-2.01775800
H	-3.19370500	2.58341900	-1.85291600
H	-3.11215800	1.33733900	-3.10338000
H	-2.21710200	1.13132600	-1.59318000
H	-5.10976300	1.57346800	0.61209600
H	-3.47138600	0.20087400	1.89283300
H	-2.61495400	1.31522800	0.84445000
H	-1.36793000	-0.32912100	-0.21996000

Coordinate III (cis)

Fe	1.17240200	0.04266800	-0.03596400
N	0.99719100	-1.75620200	1.01870500
C	0.12944400	-2.00780700	2.05921400
C	0.08490100	-3.42594700	2.32244900
C	0.93364200	-4.02323300	1.43525700
C	1.50666000	-2.97346500	0.62755300
C	2.45801400	-3.16499400	-0.37697700
C	3.09174000	-2.16873400	-1.11533000
C	4.08862400	-2.39128100	-2.13412700
C	4.44151000	-1.16707600	-2.62473600
C	3.66172500	-0.18397500	-1.91228500
N	2.85735500	-0.81381100	-0.98415500
C	3.70639000	1.19191900	-2.12798200
C	2.96559900	2.16167200	-1.44694100

C	3.08265600	3.58662000	-1.64754000
C	2.21937200	4.18345900	-0.77425800
C	1.56940000	3.12638700	-0.03657600
N	2.03492000	1.90513100	-0.46837900
C	0.63605000	3.31638200	0.98270900
C	0.03493100	2.31929100	1.75297300
C	-0.88187600	2.55353800	2.84364600
C	-1.22153400	1.33037900	3.34731800
C	-0.51478700	0.34309600	2.56597100
C	-0.57788500	-1.03716500	2.76765900
H	-1.22250000	-1.38689100	3.56815900
N	0.24216000	0.96753500	1.60305600
H	-1.88066700	1.10691100	4.17639700
H	-1.20649600	3.53010600	3.17943800
H	0.37107500	4.34255300	1.21836000
H	2.03931200	5.24076100	-0.62771000
H	3.74942800	4.05839000	-2.35790400
H	4.39608800	1.54689200	-2.88763600
H	5.16266700	-0.93965300	-3.39937600
H	4.46458600	-3.36291500	-2.42814900
H	2.75122500	-4.18982900	-0.58330500
H	1.16835900	-5.07545500	1.33789600
H	-0.51353400	-3.89246900	3.09445600
S	4.54762700	-0.29752900	1.57590900
H	4.95573400	0.65656800	0.70993700
O	-0.13824900	-0.07180800	-1.33158900
H	-0.80070100	-0.76620600	-1.17815700
C	-3.60890200	1.32372500	-1.36546200
C	-4.56781900	0.43059100	-2.17539700
C	-4.69844700	-0.99977200	-1.53356100
C	-5.72434400	-0.31133200	-0.55008000

C	-6.01901900	0.70775900	-1.69263000
H	-6.25684800	1.73271300	-1.38823400
H	-6.78075700	0.35877300	-2.39260200
C	-4.98768600	0.43538900	0.55258300
C	-3.95067200	1.26966300	0.10095600
H	-3.36909200	1.85781300	0.80805500
C	-5.33645600	0.29610800	1.88090200
H	-4.81237500	0.84258400	2.66097500
H	-6.14547800	-0.35947200	2.18933100
H	-6.54476600	-0.92314300	-0.15990800
C	-5.38798700	-1.99773900	-2.47826900
H	-5.66615800	-2.90958200	-1.93467600
H	-4.70503500	-2.28936100	-3.28651600
H	-6.29523900	-1.59938800	-2.94141800
C	-3.45322100	-1.67850100	-0.95453800
H	-2.76516700	-1.95947800	-1.76374600
H	-3.72906500	-2.60220800	-0.42964500
H	-2.91525200	-1.04592800	-0.24300300
H	-4.35457500	0.48160400	-3.24963800
H	-3.67370300	2.36064400	-1.73426700
H	-2.56106800	1.02291800	-1.52348100

Coordinate V (cis)

Fe	1.77992000	-0.06959400	0.52495000
N	0.13589700	0.57139600	1.66172000
C	-0.12500600	1.87541100	2.03005600
C	-1.09190500	1.89467300	3.09964300
C	-1.39977900	0.59482900	3.38467300
C	-0.62503200	-0.22917700	2.48957200
C	-0.63996300	-1.62339700	2.46352700
C	0.10694100	-2.44242000	1.61592200

C	0.02310200	-3.88117300	1.56763700
C	0.87792100	-4.29846300	0.58725400
C	1.49120700	-3.11773100	0.03128700
N	1.01570500	-1.99823800	0.67946100
C	2.40378700	-3.10910900	-1.02388300
C	2.97192600	-1.98079600	-1.61397300
C	3.89440300	-1.99855800	-2.72462500
C	4.21240100	-0.70005100	-2.99922200
C	3.48561500	0.11989500	-2.05889100
N	2.73466900	-0.68081200	-1.22777100
C	3.53052900	1.51277700	-2.00715300
C	2.81335800	2.33133700	-1.13451500
C	2.83060600	3.77340500	-1.14723100
C	1.96735200	4.19214300	-0.17507300
C	1.41626900	3.00875600	0.43796300
C	0.46558500	3.00239000	1.45904400
H	0.14747500	3.96826900	1.83891800
N	1.95320600	1.88595000	-0.15405700
H	1.71179800	5.20699500	0.10145400
H	3.42113300	4.37795300	-1.82360700
H	4.16845200	2.00977600	-2.73146500
H	4.87198200	-0.32005000	-3.76885100
H	4.24167000	-2.89371200	-3.22448400
H	2.68397500	-4.07302700	-1.43722600
H	1.07444100	-5.31028900	0.25658200
H	-0.61873000	-4.48392100	2.19726600
H	-1.30621500	-2.11948900	3.16240600
H	-2.08788600	0.21803200	4.13045700
H	-1.47808500	2.79165400	3.56652900
S	3.56005200	-0.06158400	2.12978100
H	3.48802200	-1.38337700	2.40282200

C	-2.98317900	-1.13666400	-1.48053600
C	-4.52219900	-1.21297800	-1.48028700
C	-5.13702900	-0.31608600	-0.34658600
C	-4.93358800	0.87619900	-1.36930900
C	-5.08536000	-0.18454500	-2.50454600
H	-4.53074000	-0.02521900	-3.43451800
H	-6.13036300	-0.36852700	-2.75809700
C	-3.50144500	1.35456700	-1.30825800
C	-2.49275400	0.30705900	-1.78449600
H	-2.43003600	0.40553500	-2.87495200
C	-3.13437400	2.56834700	-0.88624700
H	-2.08860800	2.85777100	-0.83562000
H	-3.86997800	3.30750800	-0.57740500
H	-5.64702100	1.70627500	-1.31977400
C	-6.62509200	-0.62175900	-0.10681600
H	-7.08327600	0.16643800	0.50365100
H	-6.73208100	-1.56784400	0.43909200
H	-7.20686400	-0.70666300	-1.02901000
C	-4.44481900	-0.24235000	1.01556800
H	-4.49997600	-1.21243500	1.52635000
H	-4.94513400	0.49567000	1.65567800
H	-3.39348600	0.04621700	0.95729400
H	-4.87288500	-2.25022500	-1.53718900
H	-2.56681800	-1.81498600	-2.23492900
H	-2.58323600	-1.46239300	-0.51208300
O	-1.15440000	0.54430700	-1.35952000
H	-1.11429400	0.52713800	-0.38678700

Coordinate I (trans)

Fe	1.46514200	-0.00013600	0.11637700
N	1.77738300	-2.05255500	0.25108300

C	1.37905100	-2.86048900	1.27975100
C	1.66431600	-4.24058200	0.94749600
C	2.23141400	-4.23944900	-0.29419400
C	2.29525000	-2.85862600	-0.72464600
C	2.81273500	-2.40347100	-1.94759600
C	2.91205000	-1.07911800	-2.39975700
C	3.48670200	-0.65021000	-3.65874900
C	3.39620700	0.71100700	-3.69051300
C	2.76579200	1.11884900	-2.45128800
N	2.48875600	0.01337800	-1.69684400
C	2.48485100	2.43902700	-2.06961800
C	1.89736000	2.88942900	-0.87687700
C	1.64813200	4.27258000	-0.52837800
C	1.07145200	4.27079100	0.70904500
C	0.96561600	2.88646000	1.11976900
N	1.47422700	2.08052100	0.14046700
C	0.43321900	2.42861400	2.33522500
C	0.32652700	1.10265100	2.78226500
C	-0.20065000	0.67949400	4.06393900
C	-0.10102000	-0.68099600	4.10116800
C	0.48770600	-1.09199100	2.84268300
C	0.78378100	-2.41149500	2.46876300
H	0.52726900	-3.18056600	3.19178300
N	0.73007800	0.00888900	2.07117500
H	-0.39020200	-1.35191300	4.89956000
H	-0.58686500	1.34358000	4.82617500
H	0.06910000	3.19371800	3.01501700
H	0.74748600	5.11906900	1.29782500
H	1.88936900	5.12278100	-1.15299300
H	2.76426900	3.20994500	-2.78213000
H	3.72426300	1.38543800	-4.47073500

H	3.90368300	-1.31088200	-4.40761000
H	3.19129000	-3.16851400	-2.61953200
H	2.57868800	-5.08583800	-0.87231400
H	1.45527500	-5.08827500	1.58693400
S	3.72993900	0.25462800	1.15093600
H	4.27962100	-0.88620900	0.68069600
O	-0.01909100	-0.13359400	-0.58165700
C	-3.91981300	1.22412300	-1.74531700
C	-4.56694800	-0.13384100	-2.06763900
C	-3.76268900	-1.26275700	-1.36213600
C	-4.62174900	-0.94880300	-0.09771200
C	-5.75096300	-0.44562500	-1.08498900
C	-4.02500100	0.23110100	0.62928500
C	-3.36244200	1.25191900	-0.29156300
H	-3.42975800	2.25775300	0.13849400
C	-4.06148400	0.36309900	1.96058300
H	-3.65170000	1.23777300	2.46210700
H	-4.50099100	-0.40275900	2.59629700
H	-4.88250900	-1.76630200	0.58423500
H	-4.77503200	-0.23468600	-3.14003300
H	-4.65043100	2.02731800	-1.89852400
H	-3.09928800	1.41461400	-2.44839400
H	-2.29279100	1.00859900	-0.33167100
H	-3.96975200	-2.25459900	-1.76882500
H	-2.67819200	-1.13665600	-1.27798800
C	-6.66548200	0.69402400	-0.62799100
H	-7.26742000	1.06386700	-1.46840000
H	-7.36150700	0.33703100	0.14225400
H	-6.12128700	1.54233700	-0.20519400
C	-6.63211500	-1.60123200	-1.58523400
H	-7.29669000	-1.95189600	-0.78534300

H	-7.26404400	-1.26340800	-2.41685600
H	-6.05580000	-2.46162100	-1.93816900

Coordinate II (trans) – TS (-1860.77)

Fe	-1.27238900	-0.02234300	0.18745200
N	-1.48352100	2.06439800	-0.04448600
C	-1.03455100	3.02295700	0.81572300
C	-1.32295600	4.33619700	0.26958200
C	-1.94753000	4.13478800	-0.92768700
C	-2.04198900	2.69827100	-1.11257000
C	-2.62492400	2.03366600	-2.20494100
C	-2.76065200	0.65008900	-2.40951200
C	-3.39657900	0.03116600	-3.55556700
C	-3.32340700	-1.31834800	-3.36894200
C	-2.64188800	-1.53257900	-2.10788900
N	-2.31752900	-0.32328900	-1.55563400
C	-2.36158900	-2.78624100	-1.53821300
C	-1.72362200	-3.06210000	-0.31722800
C	-1.46610000	-4.37543300	0.24440600
C	-0.83188000	-4.17521000	1.43666500
C	-0.70161600	-2.73955100	1.60446900
N	-1.25248300	-2.10475400	0.52950500
C	-0.11183400	-2.07610200	2.69097100
C	0.02197900	-0.68985500	2.89677100
C	0.61585600	-0.07228800	4.06374700
C	0.53089100	1.27909500	3.88267600
C	-0.11387000	1.49080100	2.60369500
C	-0.40257900	2.74686300	2.03775300
H	-0.10169300	3.60899000	2.62669900
N	-0.40119700	0.28179100	2.03282500
H	0.86502300	2.06547600	4.54698600

H	1.03234900	-0.60933000	4.90604800
H	0.28501500	-2.70940800	3.47972400
H	-0.48267400	-4.91939600	2.14083900
H	-1.73808400	-5.31549700	-0.21815000
H	-2.68649200	-3.64873900	-2.11365900
H	-3.69410700	-2.10445700	-4.01391500
H	-3.83858300	0.56915000	-4.38418500
H	-3.03255300	2.66774000	-2.98755800
H	-2.31397500	4.87853600	-1.62351000
H	-1.07749300	5.27699200	0.74526900
S	-3.39147400	0.02766300	1.41338500
H	-4.22359900	-0.29757400	0.39937200
O	0.22745200	-0.06570700	-0.71561300
C	3.15106800	-1.03980600	0.40910100
C	4.02084400	-1.37960600	-0.81345400
C	3.32307100	-0.83142700	-2.09020600
C	4.00554900	0.53104000	-1.75741700
C	5.14084100	-0.30114400	-1.03498600
C	3.17762100	1.25380900	-0.71972600
C	2.54297400	0.36688200	0.28081600
H	2.32146800	0.85535600	1.23264700
C	3.02332700	2.59511200	-0.71229900
H	2.43657000	3.09690700	0.05388400
H	3.48029700	3.22345800	-1.47272200
H	4.30734400	1.19035300	-2.57862100
H	4.33667300	-2.42968200	-0.79935700
H	3.74559300	-1.10876700	1.33070900
H	2.35044400	-1.78424700	0.50970500
H	1.34005000	0.17710700	-0.16898500
C	5.84052200	0.31227600	0.18098500
H	6.46468300	-0.43905300	0.68201600

H	6.50072200	1.13122300	-0.13274900
H	5.14635900	0.71871600	0.92112100
C	6.21795300	-0.77717700	-2.02235800
H	6.86966200	-1.51896500	-1.54302000
H	5.80399400	-1.23642100	-2.92481200
H	6.84861400	0.06406300	-2.33672600
H	3.70482200	-1.27380800	-3.01229600
H	2.22918400	-0.84863000	-2.10940000

Coordinate III (trans)

Fe	-1.12068200	-0.07210800	-0.00282900
N	-2.60871100	0.89292600	-1.15943700
C	-2.71755800	2.25831600	-1.33356700
C	-3.55703500	2.53596900	-2.47373700
C	-3.93494700	1.33266700	-2.99635800
C	-3.33246300	0.30738500	-2.17920600
C	-3.45558300	-1.06480200	-2.38633700
C	-2.90093200	-2.07446600	-1.59566500
C	-3.10555800	-3.48980100	-1.79265600
C	-2.42731600	-4.13231300	-0.79678300
C	-1.80083900	-3.11346100	0.01128400
N	-2.10111900	-1.86910600	-0.49573400
C	-1.02677300	-3.35321100	1.14690500
C	-0.43850700	-2.38962100	1.96810600
C	0.31423600	-2.67015500	3.16819800
C	0.70097100	-1.46731100	3.68527900
C	0.18683400	-0.44604700	2.80304400
N	-0.50184100	-1.03049900	1.76689900
C	0.35115800	0.92891800	2.98342700
C	-0.17426100	1.93639900	2.17452900
C	-0.03564100	3.35275900	2.41598400

C	-0.72102300	3.99511200	1.42455600
C	-1.28561100	2.97541100	0.57339500
C	-2.09688000	3.21815500	-0.53730800
H	-2.27846400	4.25795400	-0.79167600
N	-0.93157300	1.73110200	1.04222200
H	-0.85156600	5.06025700	1.28241000
H	0.50643600	3.78810500	3.24576400
H	0.92224900	1.24498900	3.85098800
H	1.27335600	-1.27709000	4.58421100
H	0.50647700	-3.66086100	3.55976000
H	-0.88453200	-4.39125700	1.43167700
H	-2.35742000	-5.19678300	-0.61273700
H	-3.70198700	-3.92387400	-2.58495400
H	-4.06043500	-1.38117700	-3.23062600
H	-4.56548900	1.14568900	-3.85623700
H	-3.81610200	3.52745100	-2.82259400
S	-4.70710700	0.44140400	1.10885800
H	-4.18234300	-0.79537700	0.95882100
O	0.32968500	-0.13705800	-1.12514700
C	4.60535100	-0.76271100	0.91506300
C	5.13424600	-1.24391400	-0.44883000
C	3.94970700	-1.26348100	-1.45502100
C	4.23372200	0.25558500	-1.66244100
C	5.73501600	-0.05054300	-1.28006700
C	3.53866100	1.00527300	-0.53610300
C	3.72988900	0.45002600	0.74313000
H	3.25895900	0.89992000	1.61431400
C	2.76887100	2.12850300	-0.77360300
H	2.28185200	2.66078500	0.03979200
H	2.66240200	2.53919500	-1.77348400
H	4.03719200	0.70461000	-2.64174800

H	5.74898700	-2.14560700	-0.34595300
H	5.43709500	-0.53832600	1.60190400
H	4.03773900	-1.57283100	1.40087200
H	1.05450300	0.49319600	-0.94646400
H	4.15153400	-1.86108200	-2.34593300
H	2.96051500	-1.52490900	-1.06497400
C	6.55631900	1.02419300	-0.56203200
H	7.51114300	0.60681100	-0.21697600
H	6.78845700	1.84811400	-1.24928700
H	6.04353700	1.45086800	0.30373200
C	6.54480500	-0.54049700	-2.49175200
H	6.75901500	0.29441000	-3.17090800
H	7.50635800	-0.95424300	-2.16199100
H	6.03403600	-1.31510400	-3.07094500

Coordinate V (trans)

Fe	-1.94003500	0.06607200	0.29143800
N	-2.30161400	-0.37599000	-1.71699300
C	-2.58026700	0.54076600	-2.70623500
C	-3.06442200	-0.14261400	-3.88209800
C	-3.07719700	-1.47596500	-3.59107300
C	-2.60151700	-1.61592600	-2.23522300
C	-2.46063400	-2.82455700	-1.55471500
C	-1.97643200	-2.98789600	-0.25681600
C	-1.78677800	-4.25591000	0.40343300
C	-1.26216400	-3.98992600	1.63605400
C	-1.12610000	-2.55783000	1.73614400
N	-1.57731100	-1.96524600	0.57626500
C	-0.58907300	-1.87919600	2.83024600
C	-0.38725200	-0.50335000	2.93036600
C	0.17062100	0.17360000	4.07499400

C	0.18852900	1.50707000	3.78146300
C	-0.35768400	1.65498600	2.45475200
N	-0.69248700	0.41656200	1.94752900
C	-0.52470200	2.86663500	1.78484800
C	-1.05159500	3.03443400	0.50414200
C	-1.15888000	4.29355500	-0.19048900
C	-1.69020700	4.02737400	-1.42024000
C	-1.91218100	2.60400500	-1.48501100
C	-2.41063600	1.92028800	-2.59372300
H	-2.67137200	2.51860600	-3.46126300
N	-1.52493000	2.01847900	-0.29836000
H	-1.90554200	4.72317700	-2.22100100
H	-0.85385900	5.25014600	0.21398700
H	-0.20228200	3.76350100	2.30470700
H	0.53932200	2.32393200	4.39904900
H	0.50420400	-0.31723100	4.98029300
H	-0.28442400	-2.48236300	3.67990000
H	-0.97838500	-4.69289700	2.40870800
H	-2.01760300	-5.21956600	-0.03220000
H	-2.73506600	-3.72647900	-2.09305500
H	-3.37403900	-2.29827800	-4.22940100
H	-3.34827100	0.34430600	-4.80630300
S	-4.13489800	0.37093400	1.19245000
H	-4.41608700	-0.94810100	1.28103000
C	3.31963200	-0.17265400	-1.98607200
C	4.55865400	-1.02454700	-1.65998600
C	5.64707200	-0.18008800	-0.90665000
C	4.79054300	-0.49022500	0.38823200
C	4.31639100	-1.79148400	-0.32951000
H	3.29773900	-2.12775800	-0.12462800
H	5.00894400	-2.62229200	-0.18191300

C	3.64280100	0.48308300	0.46735300
C	2.66958300	0.42859700	-0.71432200
C	3.46266400	1.34564900	1.47393100
H	2.64812500	2.06737400	1.47153000
H	4.12235100	1.35998600	2.33893800
H	5.33030700	-0.56961700	1.33874400
C	7.01181600	-0.88586900	-0.87368800
H	7.67891800	-0.39772700	-0.15196900
H	7.49109000	-0.82851200	-1.85948100
H	6.94491500	-1.94308900	-0.60090600
C	5.87237800	1.28134400	-1.30392800
H	6.31678900	1.34180400	-2.30584400
H	6.56964200	1.76156900	-0.60532200
H	4.95652200	1.87830300	-1.30728500
H	4.89084600	-1.59130800	-2.53811300
H	2.55854900	-0.78665700	-2.48111200
H	3.58465200	0.62761100	-2.68625000
O	1.54750000	-0.42112800	-0.38923000
H	2.29401100	1.44008800	-0.92273000
H	1.14588800	-0.08905000	0.43297900
